# P-selectin delineates conserved functional heterogeneity in early hematopoietic stem cell aging in humans and mice

**DOI:** 10.1038/s44318-026-00887-w

**Published:** 2026-08-05

**Authors:** Chong Yang, Tong Wang, Yue Chai, Lisha Wang, Yixuan Wang, Weili Wang, Fang Dong, Toshio Suda

**Affiliations:** 1 State Key Laboratory of Experimental Hematology, National Clinical Research Center for Blood Diseases, Haihe Laboratory of Cell Ecosystem, Institute of Hematology & Blood Diseases Hospital, Chinese Academy of Medical Sciences & Peking Union Medical College, Tianjin, China; 2 Tianjin Institutes of Health Science, Tianjin, China

**Keywords:** Immunology, Molecular Biology of Disease, Stem Cells & Regenerative Medicine

## Abstract

Progressive aging of bone marrow hematopoietic stem cells (HSCs) underlies clonal hematopoiesis and age-associated hematologic disorders. Defining early molecular events driving HSC functional decline is essential for rejuvenation strategies. Here, we identify P-selectin (Selp) as a surface marker that stratifies HSCs into conserved functional and transcriptional states during organismal aging in humans and mice. P-selectin expression increases early during aging and remains elevated in old HSCs. Selp^high^-HSCs exhibit increased DNA damage and bias towards megakaryocytic/myeloid lineage fate, whereas Selp^low^-HSCs maintain metabolic integrity, enhanced antioxidant capacity, and reduced myeloid skewing. Transcriptomic analysis revealed that Selp^high^-HSCs adopt megakaryocytic-primed, pro-inflammatory, and oxidative stress programs, while Selp^low^-HSCs retain lymphoid-associated and redox-balanced signatures consistent with a more preserved stem-cell state. Further ATAC-seq analysis demonstrates distinct chromatin landscapes with Selp^high^-HSCs being enriched for inflammatory and platelet-related regulatory elements and CTCF motifs, but Selp^low^-HSCs displaying accessible ETS-driven networks linked to metabolic fitness and stem cell resilience. Together, these findings uncover conserved heterogeneity during blood stem cell aging and establish P-selectin as an early biomarker and potential therapeutic target to mitigate age-associated hematopoietic decline.

## Synopsis

This study identifies P-selectin (Selp) as an evolutionarily conserved surface marker that captures functional and transcriptomic heterogeneity within the elderly hematopoietic stem cell (HSC) compartment. Early aged HSC pools are stratified by Selp expression levels into functionally divergent populations, driven by separate transcriptional programs and chromatin landscapes.

- P-selectin marks conserved early aging HSC states in both mice and humans.
- P-selectin expression stratifies early aged HSCs into more vulnerable Selp^high^-state and more resilient Selp^low^-state.
- Selp-defined early-aged HSC states are characterised by distinct transcriptional and epigenetic regulatory programs.

## Abstract (web summary)

P-selectin captures distinct functional and transcriptomic states in aged blood stem cells.

## Graphical abstract

**Figure:**
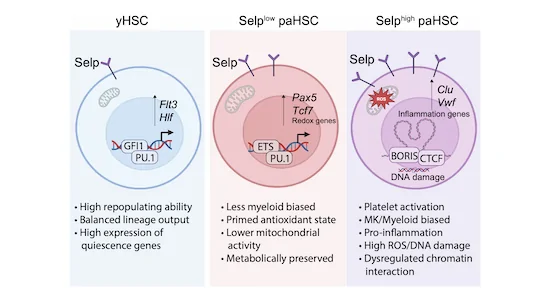

## Introduction

Hematopoietic stem cells (HSCs) maintain life-long blood production through their capacity for self-renewal and multilineage differentiation. However, aging imposes profound functional and molecular alterations on HSCs, leading to diminished regenerative potential, lineage skewing, and an increased risk of hematologic malignancies and clonal hematopoiesis of indeterminate potential (CHIP) (Beerman et al, 2010; Jaiswal et al, 2014; Kasbekar et al, 2023). Aged HSCs typically display elevated levels of reactive oxygen species (ROS), mitochondrial dysfunction, and transcriptional reprogramming that favors myeloid over lymphoid differentiation (Su et al, 2024). These changes collectively compromise hematopoietic homeostasis and contribute to immune senescence and anemia in the elderly (Fujino et al, 2022).

Increasing evidence suggests that the transition from a youthful to an aged HSC state occurs gradually, passing through a pre-aging or early-aging phase in which subtle but coordinated transcriptional, metabolic, and epigenetic changes accumulate before overt functional decline (Colom Díaz et al, 2023; Geiger et al, 2013). Elucidating the cellular and molecular mechanisms of this transitional stage is critical for uncovering early biomarkers and upstream regulators that predict or drive age-related hematologic deterioration, paving the way for targeted rejuvenation strategies.

Importantly, emerging evidence indicates that aged HSCs are not a homogeneous population but instead display functional heterogeneity shaped by distinct molecular programs and microenvironmental cues. Although surface markers such as CD150, CD41, and CD61 have been used to define biased or functionally distinct subsets within the aged HSC pool (Gekas and Graf, 2013; Mann et al, 2018; Skinder et al, 2023; Wang et al, 2025), the mechanisms linking surface phenotype to intrinsic transcriptional and metabolic states remain incompletely understood.

In this study, we focus on P-selectin (Selp, CD62P), which emerges as a candidate pre-aging marker that may link surface expression to functional heterogeneity in aged HSCs. P-selectin is a calcium-dependent adhesion molecule released from activated megakaryocytes and platelets that rapidly appears on the cell surface, and its dysregulated expression can disrupt HSC-stromal interactions and promote TGFβ-driven inflammatory and fibrotic remodeling of the hematopoietic niche (Escopy and Chaikof, 2024; Koupenova et al, 2022; Semenov et al, 1999; Spangrude et al, 2016). Moreover, P-selectin engagement with its primary ligand, P-selectin glycoprotein ligand-1 (PSGL-1), plays a critical role in maintaining HSC homeostasis, where disrupted signaling accelerates aging-associated transcriptional programs and functional decline (Yang et al, 2025). These observations position P-selectin as a central mediator integrating platelet/MK activation and inflammatory stress during HSC aging and highlight it as a potential target for restoring hematopoietic homeostasis. However, its role in the earlier pre-aging phase remains largely unknown, a gap that prompted us to investigate whether P-selectin marks early functional divergence within the aging HSC pool.

Here, we identify P-selectin as an early-aging surface marker that stratifies aged HSCs into functionally and transcriptionally distinct populations. Selp^high^ HSCs display oxidative stress, DNA damage, and myeloid/megakaryocytic bias, whereas Selp^low^ HSCs retain metabolic robustness and are primed for redox homeostasis. These subsets are governed by divergent transcriptional networks that differentially regulate redox homeostasis, chromatin organization, and inflammatory gene programs. Collectively, our findings establish P-selectin as a critical biomarker of HSC heterogeneity during early aging and suggest that selective targeting of Selp^high^ HSCs may offer a promising approach to restore hematopoietic balance and delay age-associated decline.

## Results

### Megakaryocytic lineage priming occurs during human HSC aging

**Figure 1:**
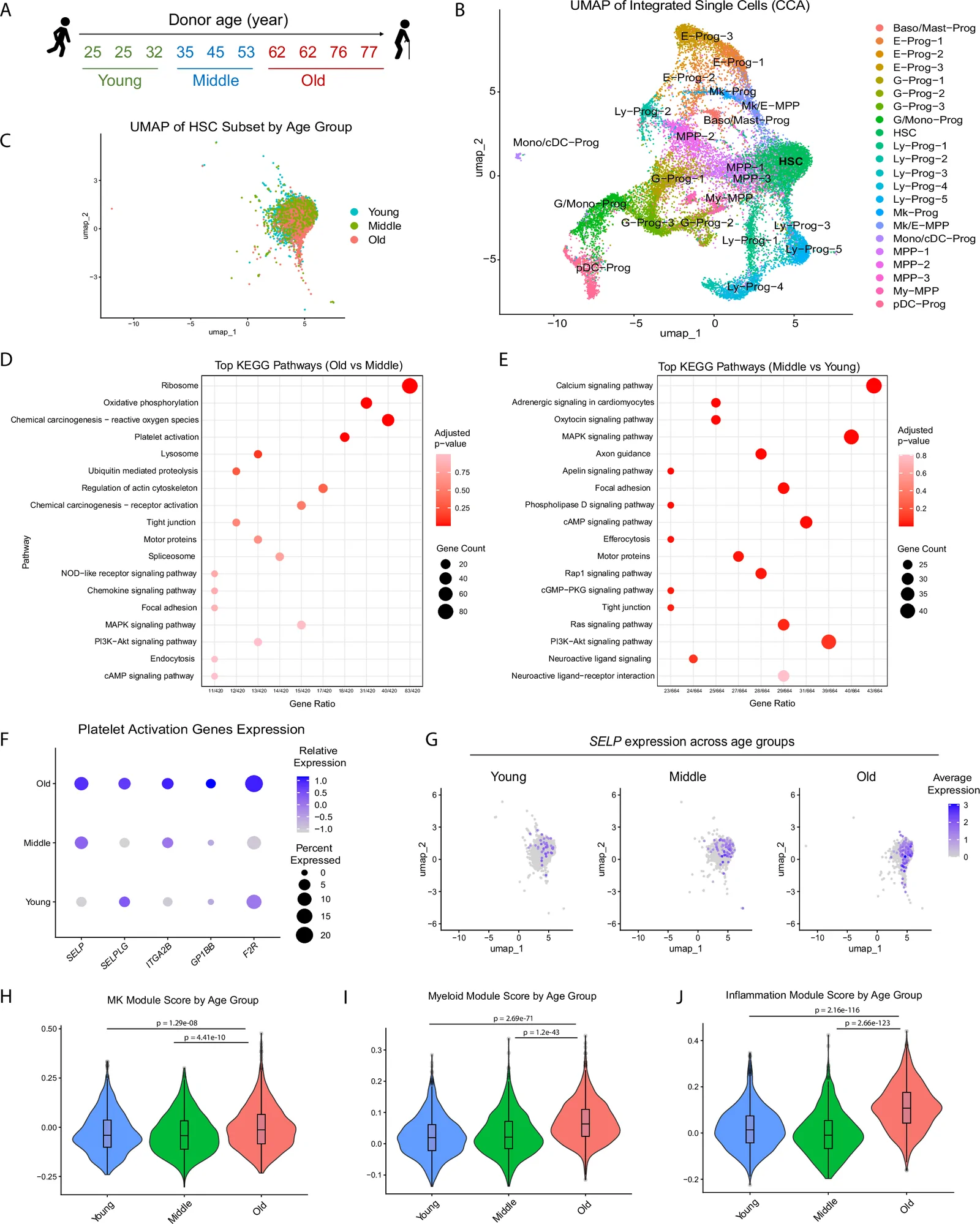
Megakaryocytic lineage-associated genes are elevated during human HSC aging. (**A**) Schematic showing donor age groups classified as Young (25–32 years), Middle (35–55 years), and Old (62–77 years). (**B**) UMAP visualization of integrated single-cell RNA-seq data from human hematopoietic stem and progenitor cells (HSPCs). Data were integrated using canonical correlation analysis (CCA), with cell-type subsets identified based on original author-provided metadata. (**C**) UMAP of the HSC subsets distinguished by age groups. (**D**, **E**) Top enriched KEGG pathways identified in differential gene expression analyses between Old vs. Middle (**D**) and Middle vs. Young (**E**) HSCs. Pathway enrichment significance was assessed using a hypergeometric test, and *P* values were adjusted for multiple comparisons using the Benjamini–Hochberg method. (**F**) Dot plot showing expression of platelet activation-related genes across age groups. (**G**) UMAP feature plots displaying SELP expression levels in HSCs from Young, Middle, and Old donors. (**H**–**J**) Violin plots comparing megakaryocytic (**H**), myeloid (**I**), and inflammation-related (**J**) gene module scores among HSCs across age groups. Statistical significance was determined using Wilcoxon rank-sum tests. In all violin plots, the overlaid box plot represents the median (center line), the 25th and 75th percentiles (box bounds), and whiskers extending to the most extreme data points within 1.5 times the interquartile range from the box bound. The number of cells (*n*) analyzed for each group is: Young, *n* = 1381; Middle, *n* = 909; Old, *n* = 1263.

**Figure EV1:**
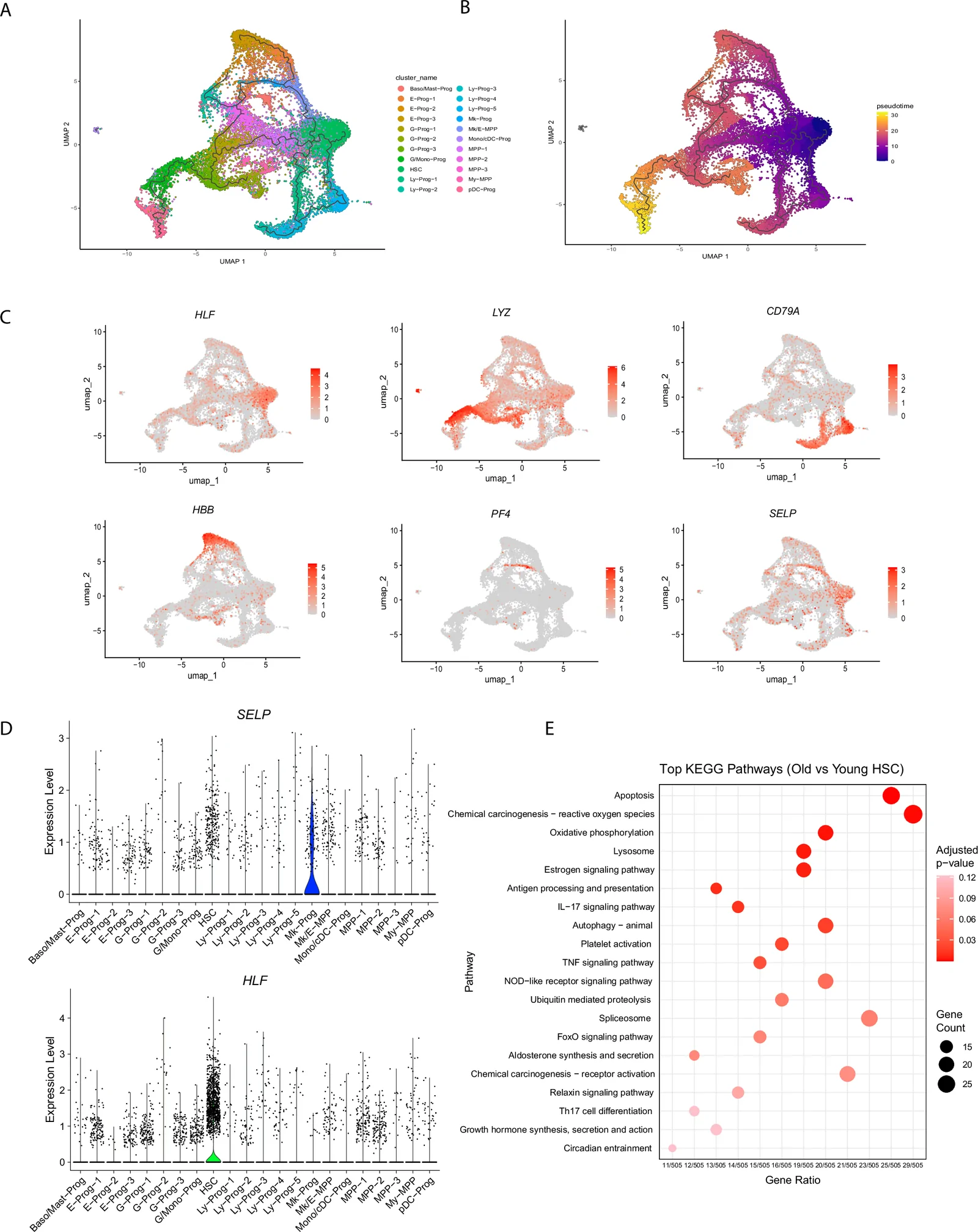
Single-cell landscape and age-associated transcriptional features of human hematopoietic stem and progenitor cells (HSPCs). (**A**) Monocle-based trajectory analysis of human HSPC single-cell RNA-seq data with cells colored by cluster identity. (**B**) The same Monocle trajectory colored by pseudotime, indicating the inferred progression from primitive HSCs toward differentiated progenitors. (**C**) UMAP feature plots displaying expression levels of representative lineage signature genes, highlighting lineage-specific transcriptional patterns. (**D**) Violin plots showing the expression distribution of SELP (top) and HLF (bottom) across defined HSPC and progenitor clusters, illustrating differential expression among cell states. The number of cells (*n*) analyzed for each cluster is as follows: Baso/Mast-Prog, *n* = 391; E-Prog-1, *n* = 1365; E-Prog-2, *n* = 132; E-Prog-3, *n* = 934; G-Prog-1, *n* = 1285; G-Prog-2, *n* = 976; G-Prog-3, *n* = 970; G/Mono-Prog, *n* = 1207; HSC, *n* = 3553; Ly-Prog-1, *n* = 932; Ly-Prog-2, *n* = 677; Ly-Prog-3, *n* = 395; Ly-Prog-4, *n* = 906; Ly-Prog-5, *n* = 881; Mk-Prog, *n* = 239; Mk/E-MPP, *n* = 855; Mono/cDC-Prog, *n* = 184; MPP-1, *n* = 1745; MPP-2, *n* = 1086; MPP-3, *n* = 171; My-MPP, *n* = 728; pDC-Prog, *n* = 903. (**E**) Dot plot of the top KEGG pathways enriched in old versus young HSCs. Dot size represents the number of genes contributing to each pathway, the *x* axis indicates the gene ratio, and color intensity reflects the adjusted *P* value. Pathway enrichment significance was assessed using a hypergeometric test, and *P* values were adjusted for multiple comparisons using the Benjamini–Hochberg method.

HSC aging is accompanied by profound alterations in molecular functions. To dissect the molecular basis of these age-associated changes and capture dynamic shifts across different phases of human HSC aging, we analyzed single-cell transcriptional profiles from a publicly available single-cell RNA-seq dataset (Li et al, 2025), across a defined range of donor ages. Bone marrow hematopoietic stem and progenitor cells (HSPCs) were profiled from young (25–32 years), middle-aged (35–53 years), and elderly (62–77 years) individuals (Fig. 1A). This allows us to identify transcriptional programs that emerge during early and late phases of aging. Integrated analysis using Seurat CCA revealed distinct clustering of HSPCs by developmental stage and lineage potential (Figs. 1B and EV1A–D).

Subsequent analyses were performed on transcriptionally defined HSCs selected from the HSPC dataset across age groups (Fig. 1C). Differential pathway analysis between age groups highlighted enrichment of platelet activation, oxidative stress, and chemokine signaling pathways in old HSCs relative to younger counterparts (Figs. 1D,E and EV1E). Notably, megakaryocytic-lineage-associated genes including *SELP, SELPLG*, and *ITGA2B* were upregulated in aged HSCs relative to young and middle-age groups, suggesting that aged HSCs are increasingly “primed” toward the megakaryocyte fate, with potential consequences for altered lineage output and functional decline (Fig. 1F,G). Module scoring further confirmed that aged HSCs exhibit elevated megakaryocytic and myeloid signatures, alongside higher inflammatory pathway activity, suggesting a shift toward a pro-inflammatory, lineage-biased state with age (Fig. 1H–J; Table EV1).

Collectively, these findings indicate that aging in human HSCs is associated with transcriptional priming toward megakaryocytic and myeloid programs, with *SELP* emerging as a prominent marker of this age-associated lineage bias.

### P-selectin identifies a distinct early-aged murine HSC population with myeloid bias and loss of quiescence

**Figure EV2:**
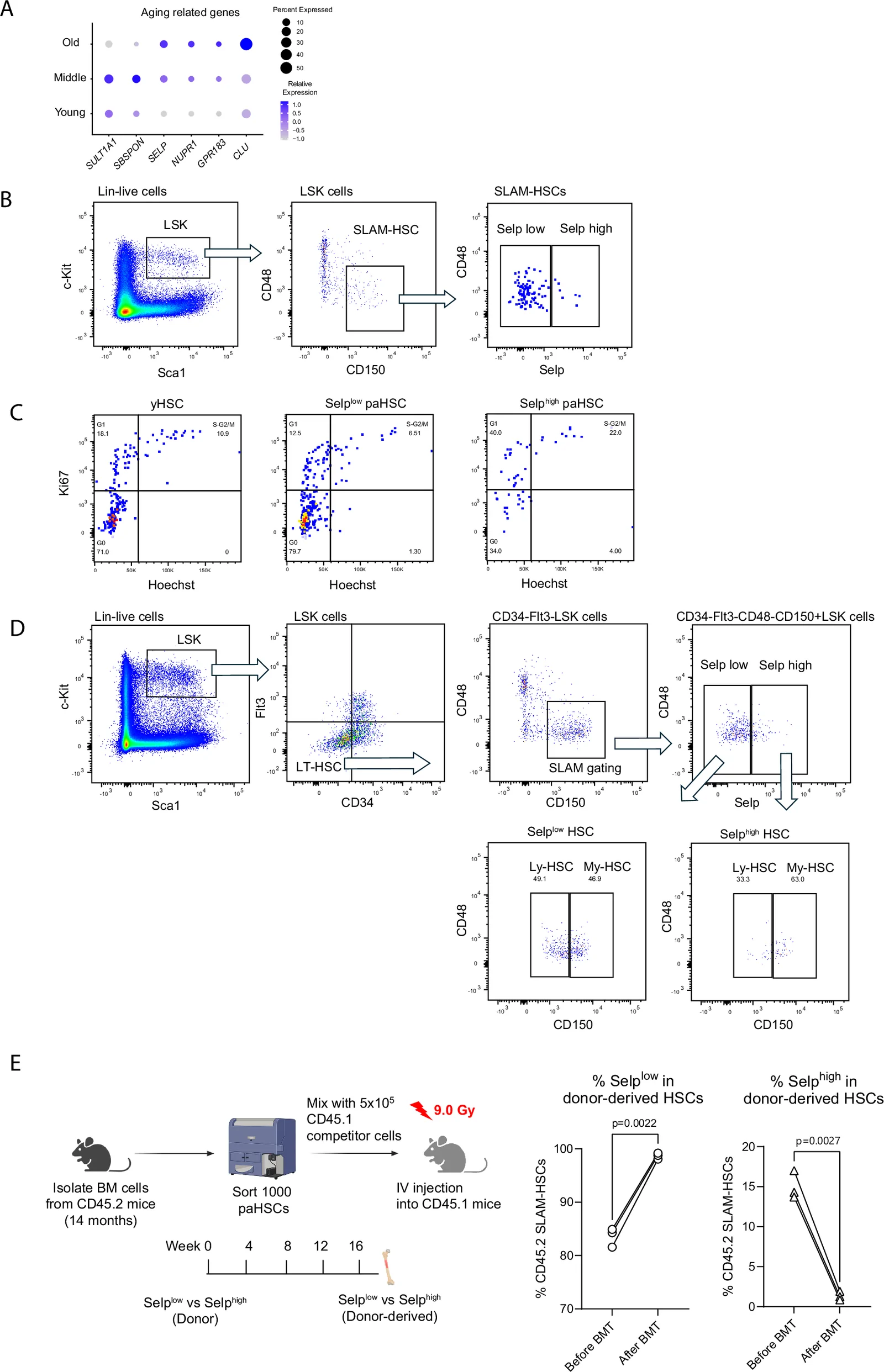
Flow cytometric identification and characterization of Selp^low^ and Selp^high^ pre-aged hematopoietic stem cells (paHSCs). (**A**) Dot plot showing the expression of aging-related genes across young, middle-aged, and old human HSCs. Dot size indicates the percentage of cells expressing each gene, and color intensity represents the average scaled expression level. (**B**) Flow cytometry gating strategy for the identification of Selp-defined HSC subsets. Lineage-negative (Lin⁻) live cells were gated to identify LSK cells (Lin⁻Sca1⁺c-Kit⁺), followed by SLAM-HSCs (CD48⁻CD150⁺). SLAM-HSCs were further subdivided into Selp^low^ and Selp^high^ populations. (**C**) Cell-cycle analysis of young HSCs (yHSC), Selp^low^ paHSCs, and Selp^high^ paHSCs based on Ki-67 and Hoechst staining, showing the distribution of cells across G₀/G₁ and S/G₂/M phases. (**D**) Extended flow cytometry gating strategy to resolve lineage-biased HSC subsets. Lin⁻ live cells were gated to identify LSK cells, followed by selection of long-term HSCs (LT-HSCs; CD34⁻Flt3⁻ LSK). Within the LT-HSC compartment, HSCs were defined as CD48⁻CD150⁺ cells and further subdivided into Selp^low^ and Selp^high^ fractions. Each fraction was subsequently resolved into lymphoid-biased HSCs (Ly-HSCs) and myeloid-biased HSCs (My-HSCs) based on differential CD150 expression. (**E**) The left panel depicts the experimental schematic of the competitive bone marrow transplantation (BMT) assay. Right panels describe the comparison of the frequency of Selp^low^ and Selp^high^ paHSCs within the donor-derived SLAM-HSC pool before BMT and 16 weeks after BMT. Data points represent individual recipient mice, and lines connect donor frequencies to their respective post-transplant outcomes. Statistical significance was determined by a two-tailed Student’s *t* test, and exact *P* values are shown.

Analysis of human HSC datasets spanning multiple ages revealed heterogeneous upregulation of aging-associated genes reported in previous studies (Itokawa et al, 2022; Sun et al, 2025; Totani et al, 2025). For example, *SULT1A1* and *SBSPON* exhibited marked increases from young to middle age but declined in old age, whereas *SELP*, *NUPR1*, and *GPR183* showed sharp elevation by middle age and remained modestly increased thereafter. In contrast, *CLU* displayed a modest rise from young to middle age, followed by a pronounced increase in old age (Fig. EV2A).

**Figure 2:**
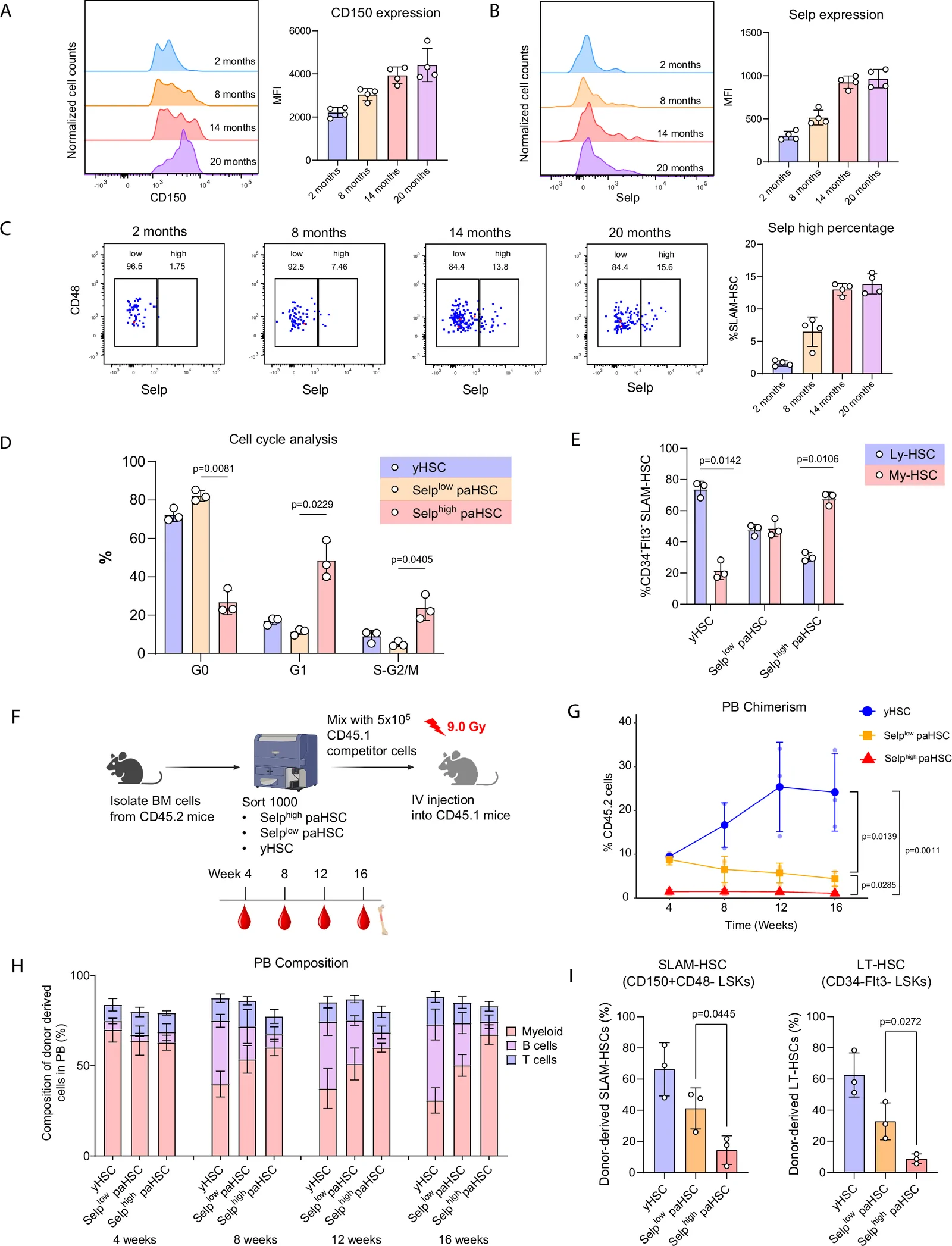
P-selectin marks early-aged murine HSCs with enhanced myeloid potential and reduced quiescence. (**A**, **B**) FACS analysis of CD150 (**A**) and P-selectin (Selp; **B**) expression in HSCs from mice at 2, 8, 14, and 20 months of age. Left: histogram overlays; right: quantification of mean fluorescence intensity (MFI). For quantification, error bars represent the mean ± SD; *n* = 4 independent mice per group. (**C**) Representative FACS plots showing the proportion of Selp^high^ SLAM-HSCs at different ages and a summary of Selp^high^ frequencies. For quantification, error bars represent the mean ± SD; *n* = 4 independent mice per group. (**D**) Cell-cycle distribution (G0, G1, S-G2/M) of young HSCs (yHSCs), Selp^low^ early-aged HSCs (paHSCs), and Selp^high^ paHSCs. For quantification and statistical analysis, error bars represent the mean ± SD; *n* = 3 independent mice per group; statistical significance was determined by two-tailed Student’s *t* test; exact *P* values are indicated in the figure, and *P *< 0.05 was considered statistically significant. (**E**) Lineage priming analysis showing distribution of myeloid-primed (My-HSC) and lymphoid-primed (Ly-HSC) in yHSCs, Selp^low^ and Selp^high^ paHSCs. For quantification and statistical analysis, error bars represent the mean ± SD; *n* = 3 independent mice per group; statistical significance was determined by two-tailed Student’s *t* test; exact *P* values are indicated in the figure, and *P* < 0.05 was considered statistically significant. (**F**) Schematic of the competitive transplantation assay. Sorted yHSCs, Selp^low^, and Selp^high^ paHSCs were mixed with competitor BM cells and transplanted into irradiated recipients. Peripheral blood was analyzed at 4, 8, 12, and 16 weeks. (**G**) Peripheral blood (PB) chimerism over time showing engraftment of yHSCs, Selp^low^, and Selp^high^ paHSCs. Individual data points were shown; lines indicate the mean value for each cohort at the indicated time points. For quantification and statistical analysis, error bars represent the mean ± SD; *n* = 3 independent mice per group. Statistical analysis was performed on log10-transformed data using two-way repeated measures ANOVA with Geisser-Greenhouse correction. Tukey’s multiple comparisons test was used to evaluate differences between populations within each time point. Exact *P* values are indicated in the figure, and *P* < 0.05 was considered statistically significant. (**H**) Donor-derived lineage composition in PB at the indicated time points, illustrating distinct lineage distributions among yHSCs, Selp^low^, and Selp^high^ paHSCs. For quantification, error bars represent the mean ± SD; *n* = 3 independent mice per group. (**I**) Quantification of donor-derived SLAM-HSCs and LT-HSCs in recipient bone marrow at 16 weeks post-transplant. For quantification and statistical analysis, error bars represent the mean ± SD; *n* = 3 independent mice per group; statistical significance was determined by two-tailed Student’s *t* test; exact *P* values are indicated in the figure, and *P* < 0.05 was considered statistically significant. LT-HSCs long-term HSCs, LSK Lin^−^Sca-1^+^c-Kit^+^. Source data are available online for this figure.

Among these candidates, *SELP* was notable for its early and sustained induction during the transition from young to middle age, suggesting that it may mark an initial shift in HSC state rather than reflecting late-stage aging alone. To test this hypothesis and determine whether Selp marks a conserved feature of HSC aging, we next turned to murine models to examine whether its expression follows a similar age-associated trajectory as observed in humans. We first analyzed Selp expression across different ages (2, 8, 14, and 20 months). Flow cytometry analysis revealed a gradual increase in the canonical SLAM marker CD150 with age (Fig. 2A), whereas both Selp expression and the proportion of Selp high HSCs rose sharply from 2 to 14 months and remained relatively stable thereafter (Figs. 2B,C and EV2B), consistent with the human data. Given that Selp expression peaks and stabilizes after 14 months, it suggests that Selp can mark early phases of HSC aging in mice. Many hallmarks of hematopoietic aging, including HSC expansion and myeloid bias, appear by 9 to 12 months of age in C57BL/6 mice, corresponding to middle age in humans and suggesting that functional decline initiates during this transitional phase (Colom et al, 2023; Young et al, 2021). Accordingly, HSCs from 6 to 10-week-old and 14 to 16-month-old mice were used to represent young (yHSCs) and early-aged (paHSCs) groups, respectively, with the latter reflecting early phases of HSC aging before the onset of overt age-associated decline.

To examine whether Selp expression is associated with distinct proliferative states during aging, we assessed the cell-cycle distribution of young and early-aged HSCs using transcriptional cell-cycle scoring. Compared with yHSCs, Selp^low^ paHSCs exhibited a modest increase in the proportion of cells in the G0 phase and a corresponding decrease in G1/S/G2M phases. In contrast, Selp^high^ paHSCs displayed reduced G0 occupancy and enrichment in G1/S/G2M phases, suggesting enhanced cycling activity that may reflect chronic inflammatory, lineage bias, or compensatory proliferation (Figs. 2D and EV2C). Together, these findings highlight a potential link between Selp-associated signaling and dysregulated cell-cycle control in hematopoietic stem cell aging.

In the bone marrow, myeloid-biased hematopoietic stem cells (My-HSCs) are functionally primed to replenish myeloid and megakaryocyte lineages in response to inflammatory cues, whereas lymphoid-biased HSCs (Ly-HSCs) preferentially contribute to lymphoid output. Previous studies have shown that these subsets can be distinguished based on CD150 expression levels (Montecino-Rodriguez et al, 2019; Toghani et al, 2025). Accordingly, we assessed the distribution of My-HSCs and Ly-HSCs among yHSCs, Selp^high^ and Selp^low^ paHSCs. Interestingly, yHSCs displayed a predominance of Ly-HSCs, whereas Selp^high^ paHSCs were primarily enriched for My-HSCs, with Selp^low^ paHSCs exhibiting an intermediate distribution (Figs. 2E and EV2D). These findings suggest that Selp expression classifies early-aged HSCs along a lineage-bias continuum, with high Selp expression marking a myeloid-skewed, pro-inflammatory subset.

To determine whether this phenotypic lineage bias translates into functional differences in vivo, we performed competitive transplantation of sorted yHSCs, Selp^high^ and Selp^low^ paHSCs into irradiated recipients (Fig. 2F). Peripheral blood analysis showed that Selp^high^ paHSCs had severely impaired long-term repopulation, whereas Selp^low^ paHSCs retained modest engraftment potential, and yHSCs demonstrated the highest relative capacity to reconstitute hematopoiesis (Fig. 2G). Lineage analysis of donor-derived cells revealed that yHSCs generated the most balanced contribution across myeloid, B, and T lineages. Selp^low^ paHSCs displayed an intermediate output, whereas Selp^high^ paHSCs exhibited a pronounced myeloid-biased reconstitution (Fig. 2H). Consistently, quantification of SLAM-HSCs (Lin^-^Sca-1^+^c-Kit^+^CD150^+^CD48^−^) and long-term-HSCs (LT-HSCs; Lin^−^Sca-1^+^c-Kit^+^CD34^−^Flt3^−^) in recipient bone marrow revealed a Selp-dependent reduction in stem cell maintenance, with yHSCs showing the greatest long-term preservation, Selp^low^ paHSCs exhibiting intermediate capacity, and Selp^high^ paHSCs displaying a marked loss of stem cell persistence (Fig. 2I).

To further assess the functional stability and competitive fitness of Selp-labeled paHSCs within a regenerative context, we measured their frequency before and after a competitive bone marrow transplantation assay. Analysis of the donor-derived SLAM-HSC compartment 16 weeks post-transplantation revealed a dramatic shift in the population composition. The frequency of Selp^low^ cells within the donor-derived paHSC pool increased significantly to near homogeneity, while the Selp^high^ fraction was nearly eliminated (Fig. EV2E). These results indicate that Selp^low^ paHSCs possess a superior competitive survival and self-renewal advantage over their Selp^high^ counterparts.

Collectively, these findings confirm that Selp expression stratifies early-aged HSCs by functional state, with high Selp marking a proliferative, myeloid-biased, and functionally compromised subset.

### P-selectin stratifies early-aged murine HSCs into distinct transcriptional states

**Figure 3:**
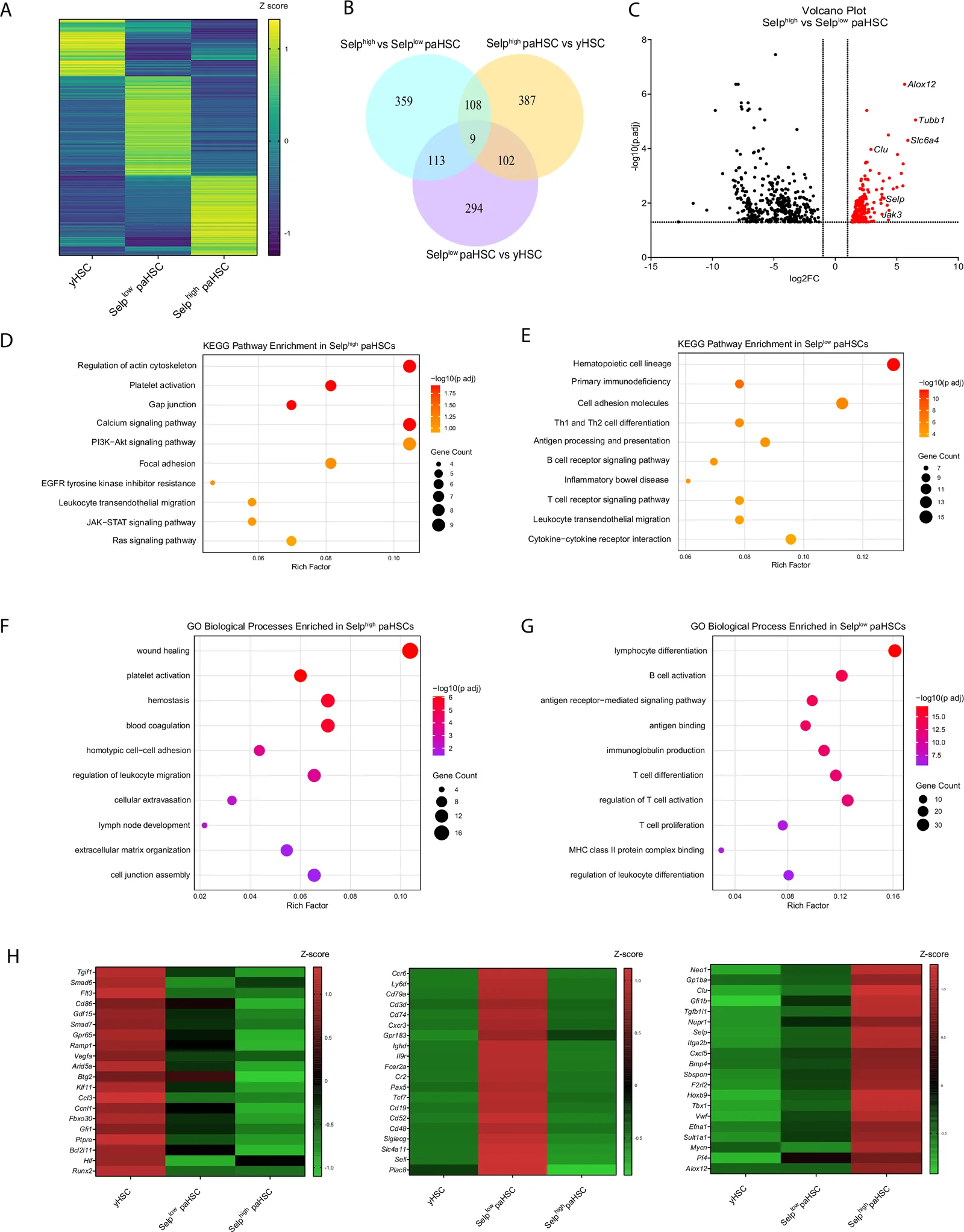
P-selectin expression distinguishes distinct transcriptional programs within early-aged murine HSCs. (**A**) Heatmap showing Z-score normalized expression of differentially expressed genes (DEGs) across yHSC, Selp^low^ paHSCs, and Selp^high^ paHSCs. (**B**) Venn diagram illustrating the overlap of DEGs among yHSC, Selp^low^, and Selp^high^ paHSC. The sum of all numbers within the circles represents the total number of differentially expressed genes, while overlapping regions indicate shared DEGs between groups. (**C**) Volcano plot highlighting DEGs between Selp^high^ and Selp^low^ paHSCs, with representative genes labeled. Statistical significance was determined using the Wald test, and *P* values were adjusted by the Benjamini–Hochberg method. (**D**, **E**) KEGG pathway enrichment analysis for upregulated genes in Selp^high^ (**D**) and Selp^low^ (**E**) paHSCs; dot size represents the number of genes and color intensity represents −log10 adjusted *P* value. (**F**, **G**) Gene Ontology (GO) biological process enrichment analysis of upregulated genes in Selp^high^ (**F**) and Selp^low^ (**G**) paHSCs; dot size represents the number of genes and color intensity represents −log10 adjusted *P* value. For all enrichment analyses, statistical significance was assessed via a hypergeometric test with Benjamini–Hochberg adjusted *P* values. (**H**) Heatmaps of representative gene sets across yHSC, Selp^low^ paHSC, and Selp^high^ paHSCs.

**Figure EV3:**
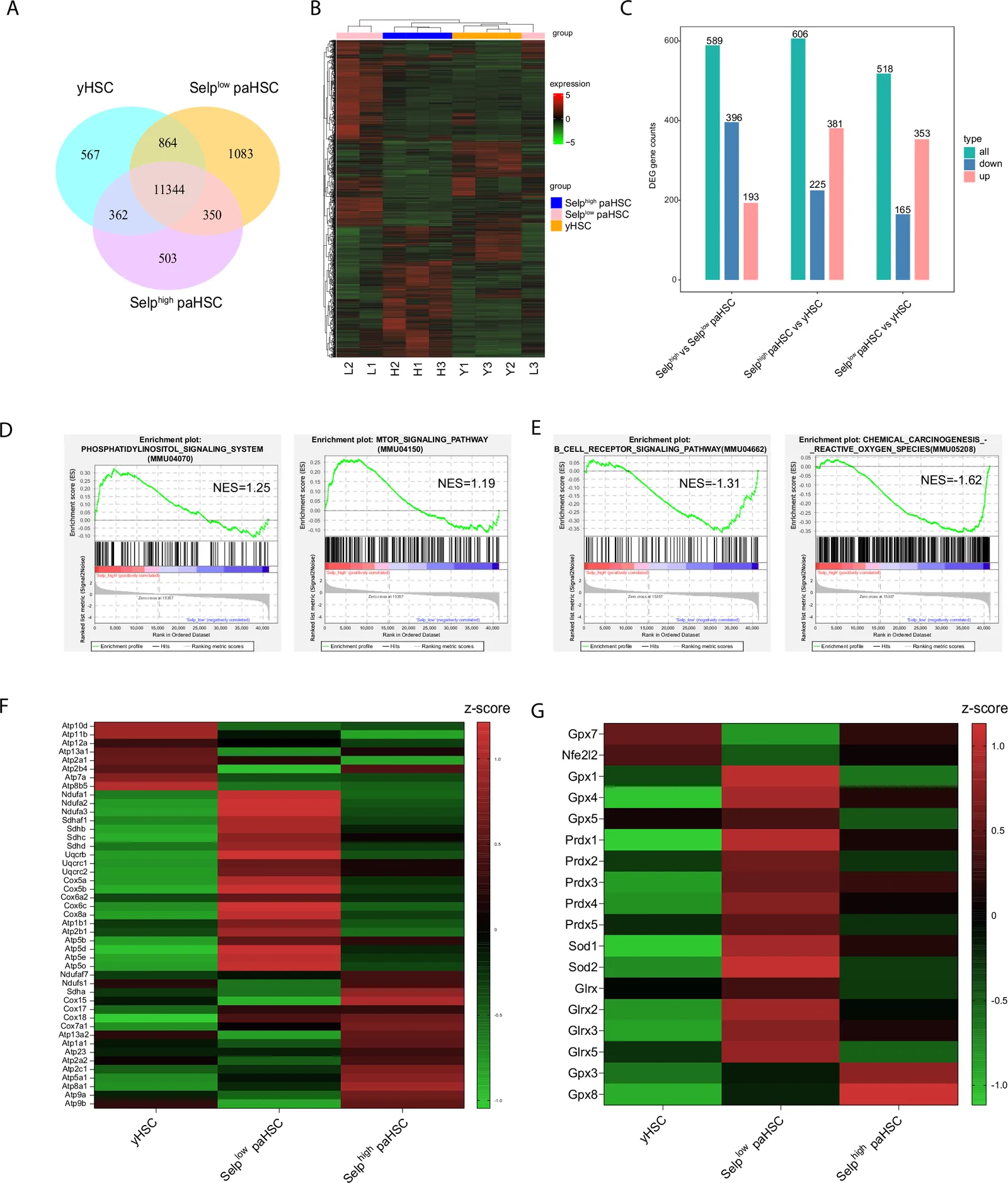
Transcriptional differences between young HSCs and Selp-defined pre-aged HSC subsets. (**A**) Venn diagram showing uniquely expressed genes in yHSC, Selp^low^ paHSC, and Selp^high^ paHSC groups. Overlapping regions indicate genes co-expressed in two or more groups. (**B**) Heatmap showing differentially expressed genes (DEGs) across yHSCs, Selp^low^ paHSCs, and Selp^high^ paHSCs. Row-normalized expression levels (Z-score) are shown for biological repeats (Y1-3, yHSCs; L1-3, Selp^low^ paHSCs; H1-3, Selp^high^ paHSCs). (**C**) Bar plot summarizing the number of DEGs between yHSC, Selp^low^ paHSC, and Selp^high^ paHSC groups. Bars indicate the total number of DEGs (all), as well as upregulated (up) and downregulated (down) genes. (**D**, **E**) GSEA plots highlighting KEGG pathways enriched in Selp^high^ (**D**) and Selp^low^ (**E**) paHSCs. (**F**, **G**) Heatmaps showing the scaled expression of mitochondrial metabolism-related genes (**F**) and antioxidant/redox-related genes (**G**) across yHSCs, Selp^low^ paHSC, and Selp^high^ paHSC. Gene expression values are Z-score normalized.

To characterize transcriptional heterogeneity associated with HSC aging, we performed RNA-seq on purified yHSCs, Selp^low^ paHSCs, and Selp^high^ paHSCs. Global transcriptomic profiling revealed that Selp^high^ paHSCs displayed a markedly distinct gene expression landscape compared with Selp^low^ paHSCs and yHSCs (Figs. 3A,B and EV3A–C). Differential expression analysis identified a subset of genes selectively upregulated in Selp^high^ paHSCs, including *Clu, Tubb1*, and *Alox12*, which are linked to megakaryocytic differentiation and inflammatory activation (Fig. 3C).

Functional pathway enrichment based on KEGG analysis demonstrated that Selp^high^ paHSCs were enriched for signaling cascades related to platelet activation, PI3K–Akt, JAK–STAT, and Ras pathways (Fig. 3D), implicating enhanced cytoskeletal remodeling, adhesion, and inflammatory signaling. In contrast, Selp^low^ paHSCs displayed enrichment for hematopoietic lineage, antigen presentation, and cytokine receptor pathways (Fig. 3E), suggestive of preserved immune and regenerative programs. Consistently, GO biological process analysis further delineated functional divergence between Selp^high^ and Selp^low^ paHSCs. Selp^high^ paHSCs were preferentially enriched for processes related to platelet activation and blood coagulation (Fig. 3F), whereas Selp^low^ paHSCs showed strong enrichment for regulation of lymphocyte activation, proliferation, and differentiation (Fig. 3G), reflecting preservation of immune-related and multilineage differentiation programs.

More specifically, yHSCs were enriched for lymphoid and stemness-associated genes, including *Flt3, Hlf, Gfi1*, and *Runx2*, consistent with a balanced, youthful transcriptional state. Selp^low^ paHSCs expressed early lymphoid and immune signaling genes such as *Cd79a, Pax5, Cd52*, and *Tcf7*, reflecting an intermediate, less inflammatory state. In contrast, Selp^high^ paHSCs showed elevated expression of megakaryocytic and pro-inflammatory regulators, including *Itga2b, Pf4, Vwf, Tgfb1i1*, and *Cxcl5*, marking a myeloid-skewed, inflammatory transcriptional program (Fig. 3H).

GSEA of paHSCs stratified by Selp expression revealed trends toward distinct pathway-level programs. Selp^high^ paHSCs showed relative enrichment for phosphatidylinositol and mTOR signaling, indicative of increased proliferative potential and potentially heightened inflammatory signaling, whereas Selp^low^ paHSCs exhibited relative enrichment for B-cell receptor signaling and reactive oxygen species pathways, suggesting preserved lymphoid priming and an increased capacity for redox control (Fig. EV3D,E). Consistent with this pathway enrichment, gene expression analysis revealed distinct redox and metabolic programs between Selp^low^ and Selp^high^ paHSCs. Selp^low^ paHSCs exhibit broader and more coordinated activation of antioxidant and redox-regulatory genes, together with differential regulation of mitochondrial oxidative phosphorylation components, consistent with preserved mitochondrial metabolism and enhanced redox regulation. In contrast, Selp^high^ paHSCs show a more restricted redox gene signature accompanied by an alternative pattern of mitochondrial gene regulation, indicative of metabolic rewiring toward a pro-inflammatory and stress-prone state (Fig. EV3F,G).

Together, these data indicate that Selp expression stratifies early-aged HSCs into functionally distinct states, with Selp^high^ cells acquiring a megakaryocytic-primed, pro-inflammatory transcriptional program, whereas Selp^low^ cells retain features of a metabolically and immunologically preserved, multipotent HSC state.

### P-selectin-expressing HSCs exhibit distinct pro-inflammatory and mitochondrial oxidative features

**Figure 4:**
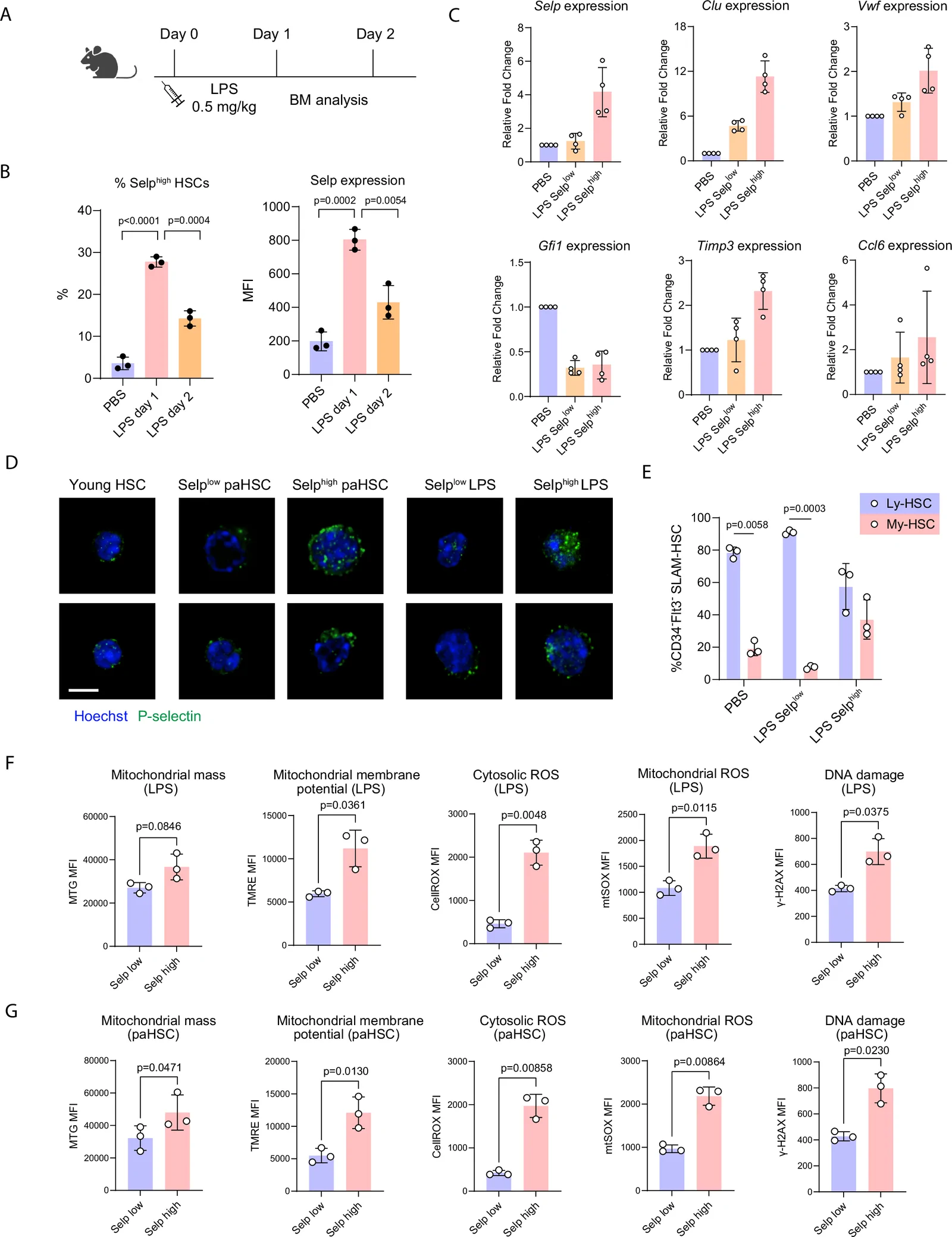
P-selectin mediates LPS-induced pro-inflammatory and mitochondrial stress features in HSCs resembling early aging. (**A**) Schematic illustration of the LPS treatment with bone marrow harvested at day 1 and day 2 for analysis. (**B**) Flow cytometric quantification of Selp^high^ HSC frequency (left) and P-selectin MFI (right) following PBS or LPS treatment. For quantification and statistical analysis, error bars represent the mean ± SD; *n* = 3 independent mice per group; statistical significance was determined by two-tailed Student’s *t* test; exact *P* values are indicated in the figure, and *P* < 0.05 was considered statistically significant. (**C**) qPCR analysis of selected gene expression in Selp^high^ and Selp^low^ HSCs following LPS treatment, with bulk HSCs from PBS-treated mice included as controls. For quantification, error bars represent the mean ± SD; *n* = 4 independent mice per group. (**D**) Immunofluorescence images of P-selectin (green) in young HSCs, Selp^low^ and Selp^high^ paHSCs, and LPS-induced Selp^low^ and Selp^high^ HSC subsets; nuclei stained with Hoechst (blue). Scale bar, 5 μm. (**E**) Flow cytometric quantification of Ly-HSC and My-HSC fractions in bulk HSCs following PBS treatment, and in Selp^low^ and Selp^high^ HSCs following LPS treatment. For quantification and statistical analysis, error bars represent the mean ± SD; *n* = 3 independent mice per group; statistical significance was determined by two-tailed Student’s *t* test; exact *P* values are indicated in the figure, and *P* < 0.05 was considered statistically significant. (**F**, **G**) Metabolic and oxidative stress analyses of LPS-treated HSCs comparing Selp^low^ and Selp^high^ subsets following LPS treatment (**F**) and in early-aged mice (**G**). For quantification and statistical analysis, error bars represent the mean ± SD; *n* = 3 independent mice per group; statistical significance was determined by two-tailed Student’s *t* test; exact *P* values are indicated in the figure, and *P* < 0.05 was considered statistically significant. MTG (Mito-Tracker Green: mitochondrial mass), TMRE (tetramethylrhodamine ethyl ester perchlorate: mitochondrial membrane potential), CellROX (cytosolic ROS), mtSOX (mitochondrial ROS), and γ-H2AX (DNA damage). Source data are available online for this figure.

To further assess the role of P-selectin in driving pro-inflammatory transcriptional programs that may contribute to hematopoietic inflammaging, which is a chronic, low-grade inflammatory state that accelerates stem cell dysfunction and tissue aging, we next examined whether inflammatory stress could induce similar aging-associated phenotypes in young HSCs. To this end, we employed a lipopolysaccharide (LPS)-induced systemic inflammation model (Fig. 4A). Strikingly, both the proportion of Selp^high^ HSCs and the overall P-selectin expression level markedly increased one day after LPS administration, followed by a partial normalization on day two (Fig. 4B). This transient yet robust induction prompted us to focus on subsequent analyses on day 1, when Selp expression is maximally induced, to investigate the immediate effects of acute inflammatory stress on HSC phenotypes and transcriptional programs.

Quantitative PCR analysis further confirmed that LPS stimulation upregulated *Selp* alongside its associated megakaryocytic and inflammatory signature genes (*Clu*, *Vwf*, *Timp3*, *Ccl6*), while concomitantly downregulating *Gfi1*, a transcriptional repressor of megakaryocytic differentiation (Fig. 4C). Immunofluorescence imaging validated the robust surface expression of P-selectin on Selp^high^ HSCs post-LPS challenge, closely resembling the phenotype of early-aged HSCs (Fig. 4D). Phenotypically, LPS-induced Selp^high^ HSCs were enriched for CD150^high^ My-HSCs, a feature characteristic of aged and myeloid-skewed stem cell populations (Fig. 4E).

Given the enrichment of inflammatory stress pathways in Selp^high^ paHSCs, together with the compensatory activation of redox homeostasis in Selp^low^ paHSCs (Fig. EV3D–G), we next examined whether inflammatory stimulation through LPS exposure could reproduce these metabolic and oxidative features in young HSCs. Flow cytometric analyses revealed that LPS-induced Selp^high^ HSCs exhibited significantly elevated mitochondrial membrane potential, increased intracellular reactive oxygen species (ROS) and mitochondrial superoxide levels, as well as heightened γ-H2AX staining intensity indicative of DNA damage, compared with their Selp^low^ counterparts (Fig. 4F). Notably, early-aged HSCs exhibited comparable oxidative profiles, reinforcing that P-selectin upregulation is coupled to mitochondrial hyperactivation and oxidative stress accumulation (Fig. 4G). Interestingly, although mitochondrial metabolism-related genes are differentially expressed between Selp^low^ and Selp^high^ paHSCs, Selp^low^ paHSCs exhibited markedly lower levels of ROS and DNA damage despite active mitochondrial gene expression, likely due to the high expression of multiple redox-related genes (Fig. EV3F,G). These findings suggest that Selp^low^ HSCs sustain mitochondrial activity without inducing excessive oxidative stress and represent a metabolically competent, resilient subset of HSCs, in contrast to Selp^high^ HSCs, which display mitochondrial hyperactivation accompanied by oxidative stress accumulation.

Together, these findings demonstrate that inflammatory stress rapidly triggers P-selectin induction in HSCs, driving a megakaryocytic-primed, oxidative, and pro-inflammatory transcriptional state that mirrors the early aging phenotype. This establishes a mechanistic link between inflammation-induced P-selectin signaling and hematopoietic stem cell dysfunction during early aging.

### Divergent chromatin accessibility landscapes govern P-selectin-defined early HSC aging states

**Figure 5:**
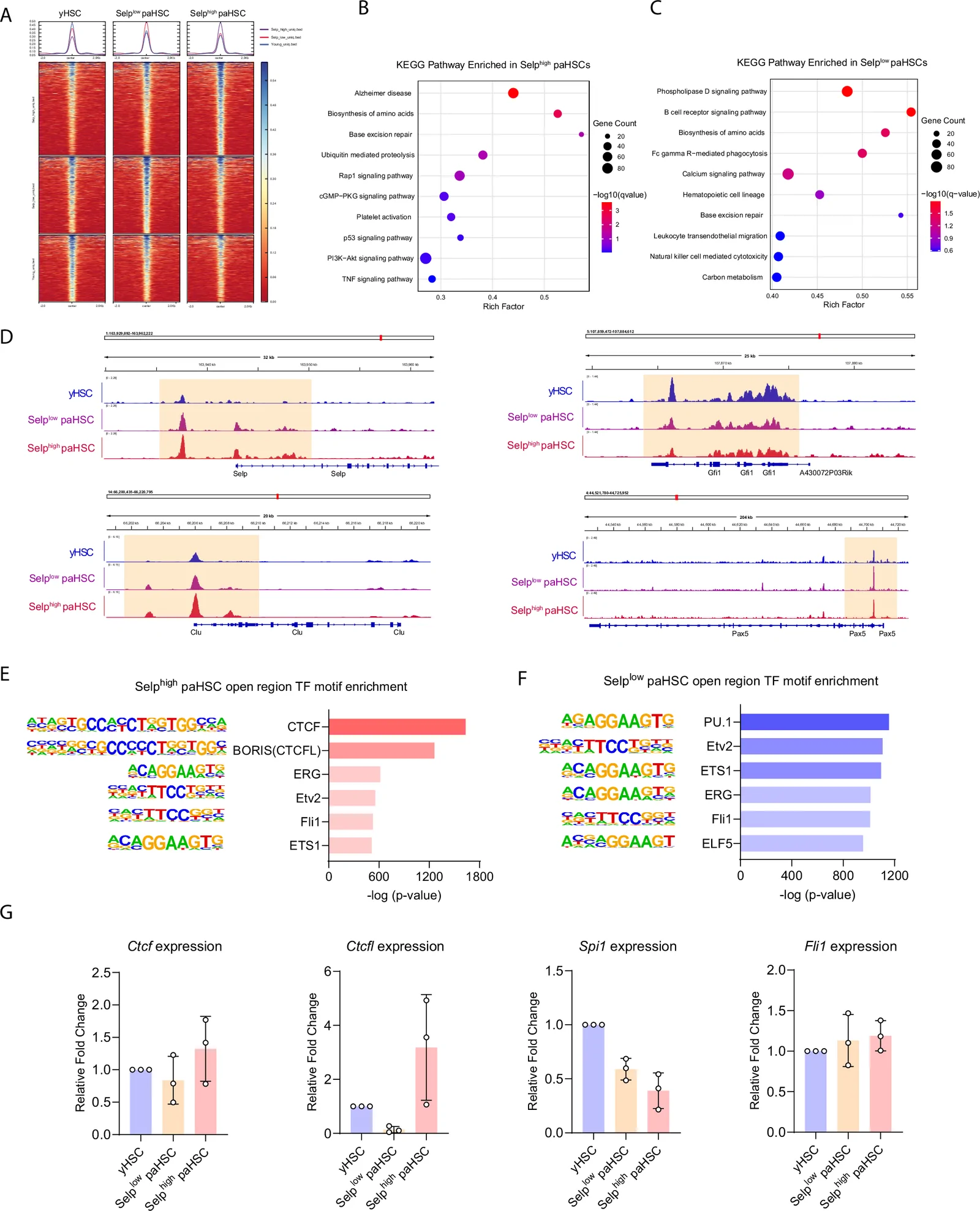
Distinct chromatin landscapes underlie transcriptional diversity among Selp-defined early-aged HSCs. (**A**) ATAC-seq heatmap showing chromatin accessibility profiles of yHSCs, Selp^low^ and Selp^high^ paHSCs. Representative aggregate peak plots (top) illustrate global accessibility differences across groups. (**B**, **C**) KEGG pathway enrichment of genes associated with differential open chromatin regions from Selp^high^ (**B**) and Selp^low^ (**C**) paHSC-specific open chromatin regions. Differential accessibility analysis between Selp^high^ and Selp^low^ paHSCs was first performed to identify group-specific accessible regions. Statistical significance of pathway enrichment was assessed using a hypergeometric test, and *P* values were adjusted for multiple comparisons using the Benjamini–Hochberg method. (**D**) Genome browser tracks of representative loci showing differential chromatin accessibility. (**E**, **F**) Transcription factor motif enrichment within Selp^high^ (**E**) and Selp^low^ (**F**) paHSC-specific accessible regions. Motif enrichment was analyzed using HOMER. *P* values represent hypergeometric tests comparing motif frequencies in target peaks versus GC-matched background sequences. (**G**) qPCR validation of selected transcription factor gene expression. For quantification, error bars represent the mean ± SD; *n* = 3 independent mice per group. Source data are available online for this figure.

**Figure EV4:**
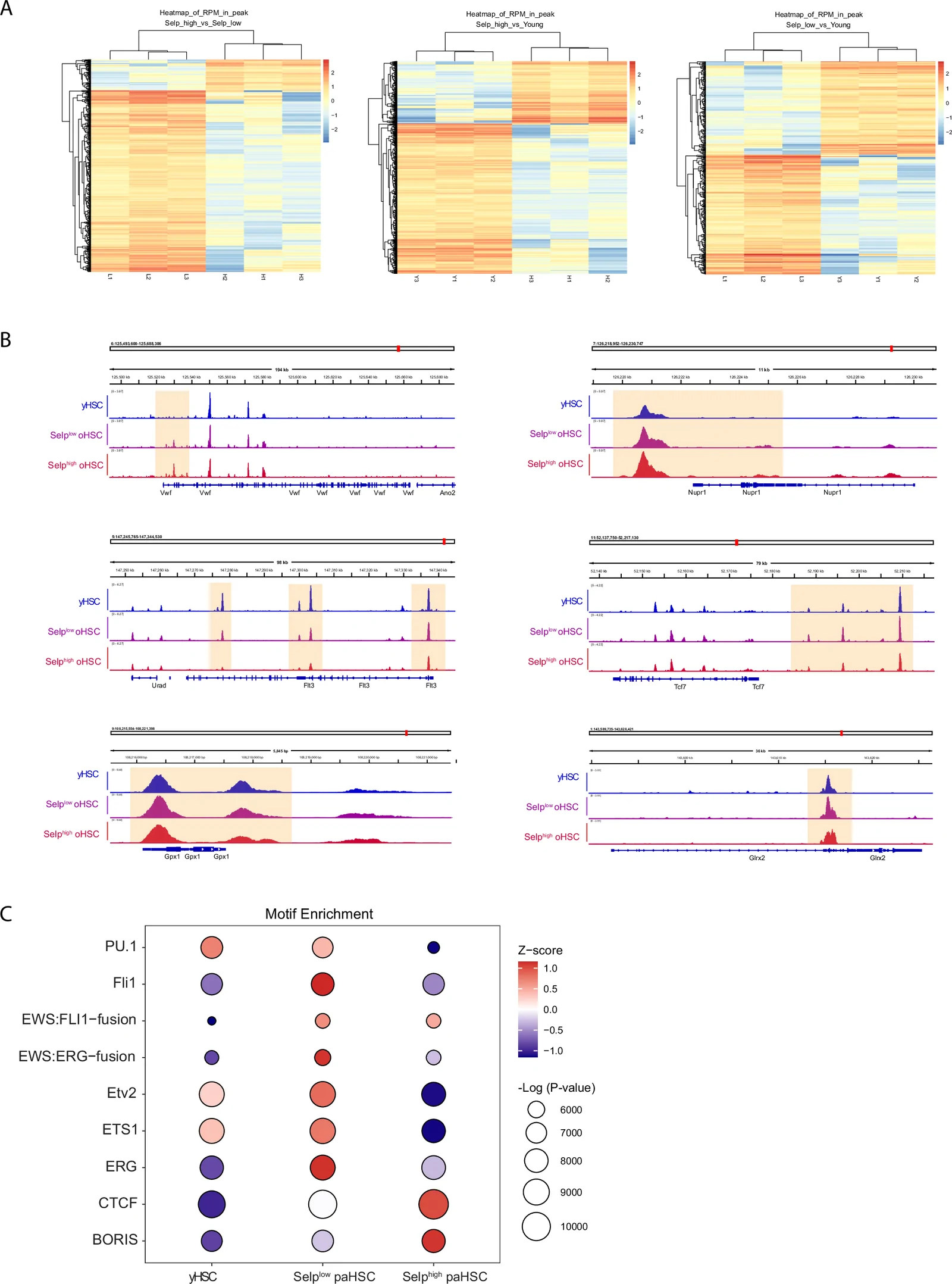
Chromatin accessibility differences and transcription factor motif enrichment in Selp-defined HSC subsets. (**A**) Heatmaps showing normalized chromatin accessibility across differentially accessible regions comparing Selp^high^ paHSC versus Selp^low^ paHSCs (left), Selp^high^ paHSC HSCs versus yHSCs (middle), and Selp^low^ paHSCs versus yHSCs (right). Rows represent ATAC-seq peaks and columns represent biological repeats; hierarchical clustering was applied to both peaks and samples (Y1-3, yHSCs; L1-3, Selp^low^ paHSCs; H1-3, Selp^high^ paHSCs). (**B**) Representative genome browser tracks showing chromatin accessibility at selected loci in yHSCs, Selp^low^ paHSCs, and Selp^high^ paHSCs. (**C**) Transcription factor motif enrichment analysis based on differentially accessible regions in yHSCs, Selp^low^ paHSCs, and Selp^high^ paHSCs. Dot color indicates motif enrichment Z-score, and dot size reflects −log10(*P* value). Motif enrichment was analyzed using HOMER. *P* values represent hypergeometric tests comparing motif frequencies in target peaks versus GC-matched background sequences.

To investigate the upstream regulatory mechanisms underlying Selp^high^ and Selp^low^ paHSCs, we performed ATAC-seq on sorted HSC populations from young and early-aged mice. Heatmap analysis of chromatin accessibility revealed distinct open chromatin profiles in Selp^high^ versus Selp^low^ paHSCs compared with yHSCs (Figs. 5A and EV4A), suggesting differential regulatory potential associated with P-selectin expression.

KEGG pathway enrichment analysis of genes associated with Selp^high^ paHSC open chromatin regions indicated activation of pathways related to platelet function, PI3K-Akt signaling, and DNA repair (Fig. 5B), consistent with the pro-inflammatory, myeloid-biased phenotype and transcriptomic signatures of Selp^high^ paHSCs. In contrast, Selp^low^ paHSCs were enriched for B-cell receptor signaling, hematopoietic cell lineage, and metabolic pathways (Fig. 5C), consistent with their preserved metabolic competence and lymphoid-biased transcriptomic profiles relative to their Selp^high^ counterparts.

Furthermore, genome browser tracks revealed differential chromatin accessibility at key loci. Selp^high^ paHSCs exhibited increased accessibility at inflammatory and myeloid/megakaryocyte-associated genes, including *Selp, Clu, Vwf*, and *Nupr1*, whereas Selp^low^ paHSCs showed elevated chromatin accessibility at lymphoid lineage genes, including *Pax5* and *Tcf7*, as well as antioxidant genes such as *Gpx1* and *Glrx2*, consistent with their lymphoid-biased potential and redox regulation. Young HSCs showed the highest accessibility at loci such as *Gfi1* and *Flt3*, indicative of preserved multipotency, with *Gfi1* supporting quiescence and balanced lineage output, and *Flt3* priming for lymphoid differentiation (Figs. 5D and EV4B).

Motif enrichment analysis of differentially accessible chromatin regions revealed distinct transcription factor signatures across subsets, characterized by a stepwise loss of lineage master regulators, a transient activation of stemness-associated factors within the Selp^low^ fraction, and a continuous accumulation of architectural stress-associated motifs in the Selp^high^ population (Fig. EV4C). More specifically, while yHSCs displayed a relatively restricted motif landscape, paHSCs exhibited pronounced divergence upon Selp stratification. Selp^low^ paHSCs were strongly enriched for ETS-family motifs, including PU.1, ETV2, and ERG (Fig. 5E), consistent with a regulatory program associated with lineage flexibility, stem cell maintenance, and resilience to oxidative stress (Chavez et al, 2021; Iwasaki et al, 2005; Knudsen et al, 2015; Staber et al, 2013a). In contrast, Selp^high^ paHSCs showed preferential enrichment of CTCF and CTCFL (BORIS) motifs (Fig. 5F), suggesting increased involvement of chromatin architectural regulators in shaping transcriptional programs linked to myeloid bias, inflammatory signaling, and stress responses (Cui et al, 2025; Tang et al, 2024). Together, these data place Selp^high^ paHSCs and Selp^low^ paHSCs within a broader regulatory continuum defined relative to young HSCs, while highlighting distinct transcription factor dependencies that emerge with aging. Consistent with motif predictions, quantitative expression analysis showed trends of higher *Ctcf*/*Ctcf1* expression in Selp^high^ paHSCs and enrichment of *Spi1* expression in Selp^low^ paHSCs (Fig. 5G), supporting the notion that distinct transcriptional networks underlie the functional heterogeneity of early-aged HSCs.

Collectively, these results reveal that chromatin accessibility landscapes and upstream transcriptional regulators define P-selectin-associated functional states in early-aged HSCs, providing mechanistic insight into the divergence between vulnerable Selp^high^ and resilient Selp^low^ populations.

### SELP delineates distinct transcriptional states of human HSCs across aging

Having established in mice that Selp marks a pre-aging HSC subset with distinct transcriptional and functional features, we next examined whether SELP expression similarly stratifies human HSCs during aging.

**Figure 6:**
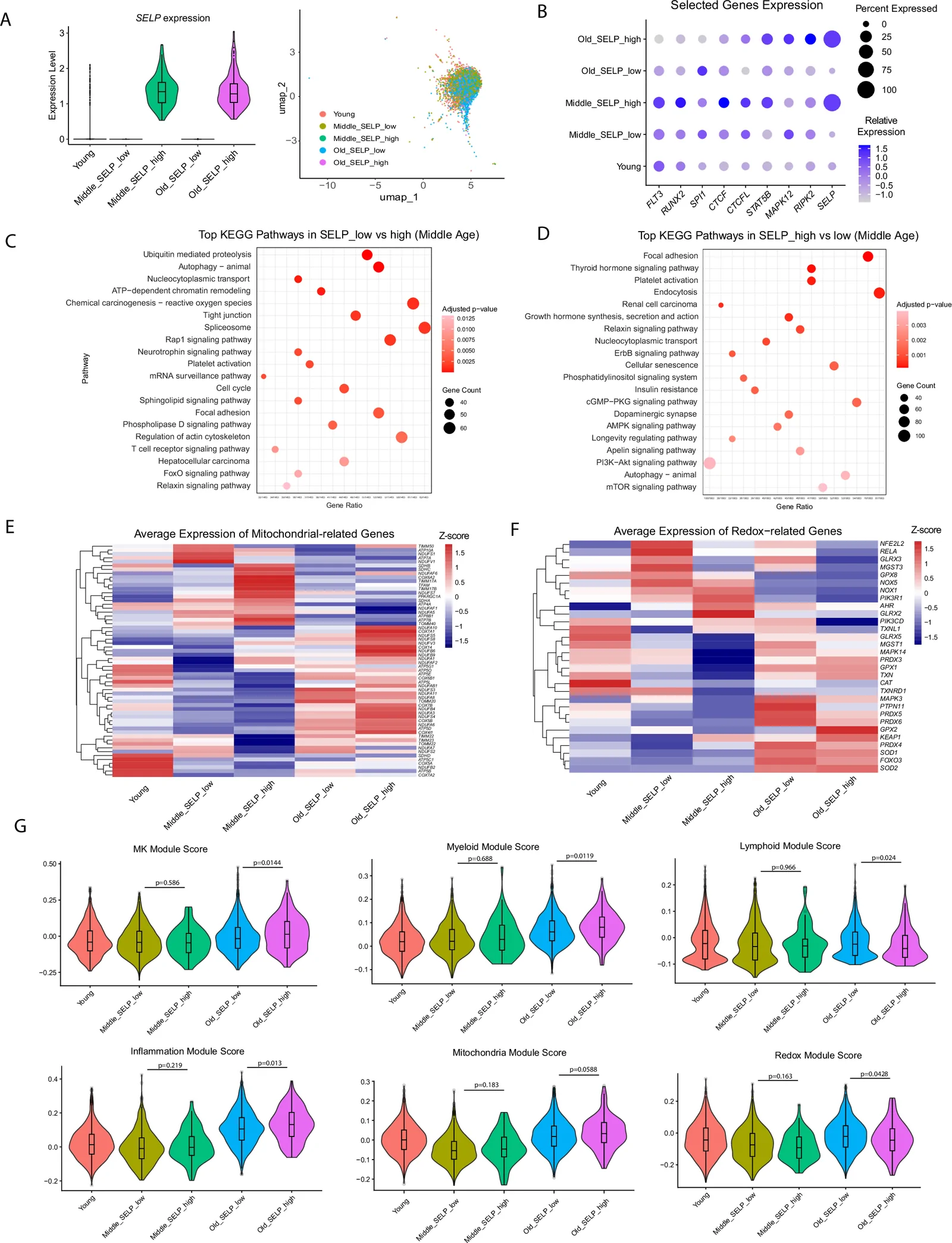
*SELP* stratifies conserved transcriptionally distinct states during human HSC aging. (**A**) Violin plot (left) and UMAP visualization (right) of *SELP* expression levels and distribution across young, middle-aged, and old HSC cohorts stratified by SELP status. In all violin plots, the overlaid box plot represents the median (center line), the 25th and 75th percentiles (box bounds), and whiskers extending to the most extreme data points within 1.5 times the interquartile range from the box bound. The number of cells (*n*) analyzed for each group is: Young, *n* = 1381; Middle_SELP_low, *n* = 836; Middle_SELP_high, *n *= 73; Old_SELP_low, *n* = 1137; Old_SELP_high, *n* = 126. (**B**) Dot plot depicting expression of representative gene signatures in *SELP*_high and *SELP*_low subsets across age groups. (**C**, **D**) KEGG pathway enrichment analyses for *SELP*_low vs *SELP*_high middle-aged HSCs (**C**), and vice versa (**D**). Statistical significance of pathway enrichment was assessed using a hypergeometric test, and *P* values were adjusted for multiple comparisons using the Benjamini–Hochberg method. (**E**, **F**) Heatmaps illustrating differential expression of mitochondrial- (**E**) and redox- (**F**) related genes. (**G**) Module score analyses for megakaryocytic, myeloid, inflammatory, lymphoid, mitochondrial, and redox-associated programs across distinct HSC types. Statistical significance was determined using Wilcoxon rank-sum tests. In all violin plots, the overlaid box plot represents the median (center line), the 25th and 75th percentiles (box bounds), and whiskers extending to the most extreme data points within 1.5 times the interquartile range from the box bound. The number of cells (*n*) analyzed for each group is: Young, *n* = 1381; Middle_SELP_low, *n* = 836; Middle_SELP_high, *n* = 73; Old_SELP_low, *n* = 1137; Old_SELP_high, *n* = 126.

To this end, we analyzed the single-cell RNA-sequencing dataset of human HSCs encompassing multiple age groups and stratified cells based on *SELP* expression and donor age (Young, Middle-aged, and Old). Because *SELP* expression was minimal in young HSCs, we further subdivided the middle-aged and old groups into *SELP*_high and *SELP*_low subsets for comparative analysis. *SELP* expression increased markedly during the transition from young to middle age and plateaued thereafter, with a *SELP*_high population emerging in middle-aged HSCs and persisting into old age (Fig. 6A).

Comparative transcriptomic analyses revealed that stem cell-associated genes, such as *FLT3*, are highly expressed in young adult HSCs, whereas inflammatory and stress-response genes, including *STAT5B, MAPK12*, and *RIPK2*, are upregulated in *SELP*_high HSCs in both middle-aged and old individuals. Interestingly, consistent with murine observations, *CTCF* and *CTCFL* expression increase sharply during middle age and are enriched in *SELP*_high HSCs, whereas *SPI1* shows the opposite trend, being predominantly expressed in *SELP*_low HSCs from middle-aged and older donors (Fig. 6B).

**Figure EV5:**
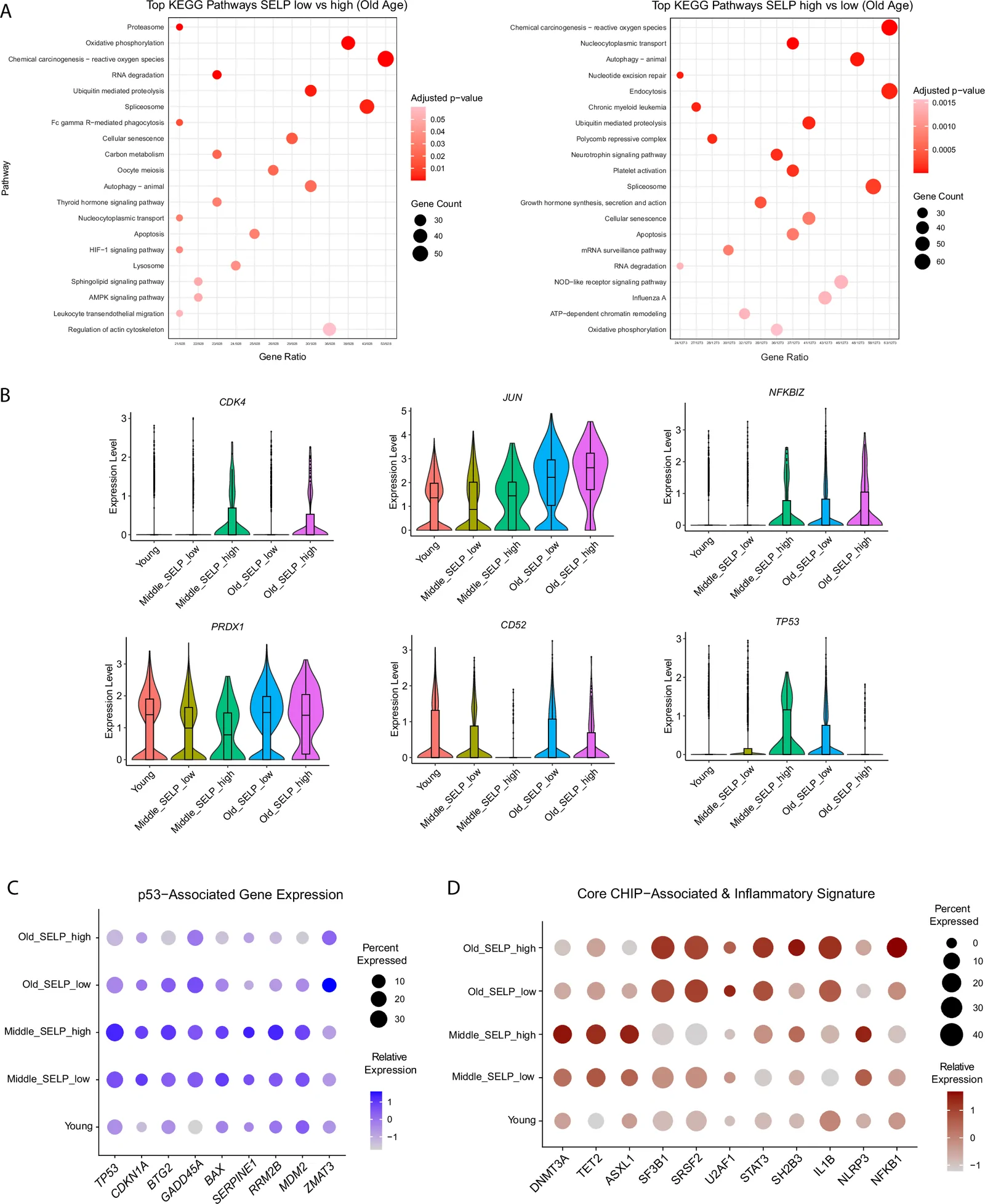
SELP stratification reveals distinct stress-response programs in human HSCs upon aging. (**A**) KEGG pathway enrichment analysis of differentially expressed genes between *SELP*_low and *SELP*_high Old HSCs in human groups. Statistical significance of pathway enrichment was assessed using a hypergeometric test, and *P* values were adjusted for multiple comparisons using the Benjamini–Hochberg method. (**B**) Violin plots showing the expression of representative genes across young, middle-aged, and old HSCs stratified by SELP expression. In all violin plots, the overlaid box plot represents the median (center line), the 25th and 75th percentiles (box bounds), and whiskers extending to the most extreme data points within 1.5 times the interquartile range from the box bound. The number of cells (*n*) analyzed for each group is: Young, *n* = 1381; Middle_SELP_low, *n* = 836; Middle_SELP_high, *n* = 73; Old_SELP_low, *n* = 1137; Old_SELP_high, *n* = 126. (**C**, **D**) Dot plot summarizing the expression of p53-associated genes (**C**) and Clonal Hematopoiesis of Indeterminate Potential (CHIP) and inflammation-associated genes. (**D**) across young, middle-aged, and old human HSCs, further subdivided into *SELP*_low and *SELP*_high fractions. Dot size indicates the percentage of cells expressing each gene, and color intensity represents the average scaled expression level.

Pathway enrichment analysis revealed that SELP stratification already delineates distinct biological programs in middle age, with further consolidation of these differences in old HSCs (Figs. 6C,D and EV5A). In middle-aged HSCs, *SELP*_low cells were preferentially enriched for pathways related to proteostasis and cellular homeostasis, including ubiquitin-mediated proteolysis, autophagy, nucleocytoplasmic transport, and redox-associated processes, consistent with an adaptive metabolic maintenance state. In contrast, *SELP*_high middle-aged HSCs showed enrichment of pathways associated with cellular activation and signaling, such as focal adhesion, platelet activation, PI3K-Akt, AMPK, and mTOR signaling, indicating early engagement of stress- and activation-related programs. In old HSCs, these SELP-associated differences became more pronounced and shifted toward overt stress and dysfunction. *SELP*_low old HSCs retained enrichment of oxidative phosphorylation, proteasome, and redox-related pathways, whereas *SELP*_high old HSCs exhibited stronger enrichment of pathways linked to reactive oxygen species, DNA damage and repair, inflammatory signaling, and senescence-associated processes. Together, these analyses suggest that SELP-dependent divergence emerges at middle age, with *SELP*_low HSCs maintaining metabolic and proteostatic capacity, while *SELP*_high HSCs progressively transition toward stress-activated and degenerative states with advancing age.

Consistent with these signatures, at the metabolic level, mitochondrial genes are activated in both *SELP*_low and *SELP*_high HSCs across middle-aged and old donors, whereas redox-related genes, particularly the master regulator *NFE2L2 (NRF2)*, are selectively upregulated in *SELP*_low HSCs, especially in middle-aged individuals (Fig. 6E,F). This pattern suggests that *SELP*_low HSCs maintain metabolic activity while preserving redox homeostasis.

Module score analysis further revealed that *SELP*_high HSCs exhibited elevated megakaryocytic, myeloid, mitochondrial, and inflammatory programs, whereas *SELP*_low HSCs maintained higher lymphoid and redox module activities (Fig. 6G; Table EV1), particularly in old HSCs, although the trend begins to emerge during middle age. At the level of individual genes, *SELP*_high HSCs consistently expressed higher *CDK4, JUN*, and *NFKBIZ* in both middle-aged and aged cells, reflecting enhanced proliferation and inflammatory priming, whereas *SELP*_low HSCs maintained higher *CD52* and *PRDX1* expression that highlighted a lymphoid potential and metabolic resilience (Fig. EV5B).

Interestingly, p53 expression was elevated in middle-aged *SELP*_high HSCs (Fig. EV5B), consistent with active DNA damage responses in stress-primed cells, as p53 pathway activation is preferentially associated with damage-exposed and stress-responsive states (Abuetabh et al, 2022). This heightened p53 activity was accompanied by a coordinated, yet transient, induction of p53-associated checkpoint, DNA repair, and senescence-related programs (*TP53, CDKN1A, BTG2, GADD45A, RRM2B, SERPINE1*), indicating an active stress-response state. In contrast, p53 expression declined in aged *SELP*_high HSCs, together with a global attenuation of p53 target gene expression, suggesting chronic stress exposure followed by exhaustion or dampening of p53-dependent adaptive pathways. By comparison, aged *SELP*_low HSCs selectively upregulated the p53 target *ZMAT3*, consistent with a shift toward transcript stabilization and long-term cellular persistence during advanced aging (Bieging-Rolett et al, 2020) (Fig. EV5C).

Furthermore, to investigate the potential link between age-associated stress and CHIP, we examined the expression of core CHIP-associated and inflammatory genes across *SELP*-defined HSC subsets. While middle-aged *SELP*_high HSCs showed an early induction of epigenetic regulators like *DNMT3A, TET2*, and *ASXL1*, aged *SELP*_high HSCs exhibited a profound upregulation of splicing factors (*SF3B1, SRSF2, U2AF1*) and signaling mediators (*STAT3, SH2B3*). This molecular profile in old *SELP*_high HSCs was coupled with a robust inflammatory signature, characterized by elevated expression of *IL1B, NLRP3*, and *NFKB1*. These findings suggest that the *SELP*_high state in advanced age is associated with an accumulation of CHIP-related mutations and a chronic pro-inflammatory environment, potentially driving the functional decline and competitive disadvantage observed in these cells. In contrast, *SELP*_low HSCs maintained lower levels of these inflammatory and CHIP-associated markers, aligning with their superior persistence and more stable molecular landscape during aging (Fig. EV5D).

Together, these findings demonstrate that SELP expression stratifies aging human HSCs into distinct functional states, with *SELP*_high cells representing a pro-inflammatory, myeloid-biased subset and *SELP*_low cells retaining metabolic resilience and lymphoid differentiation potential. Importantly, these human observations closely mirror our murine analyses, highlighting conserved mechanisms by which SELP expression delineates HSC states across species. Notably, the emergence of these transcriptional, functional, and stress-response differences during middle age defines a pre-aging phase in which early molecular and metabolic shifts set the stage for progressive HSC decline.

## Discussion

HSC function begins to decline during early aging, accompanied by subtle myeloid skewing and rising oxidative stress. In this study, we demonstrate that P-selectin expression distinguishes early-aged HSCs with divergent functional and molecular properties. Early-aged HSCs with high Selp expression exhibit elevated ROS, increased DNA damage, and a bias toward megakaryocyte/myeloid differentiation, whereas HSCs with lower Selp expression retain metabolic fitness, enhanced antioxidant capacity, and preserve lymphoid-associated transcriptional programs. These findings reveal a previously unrecognized resilient subset within the early aging HSC pool. Furthermore, ATAC-seq analysis uncovered distinct upstream regulatory networks governing these two states, providing mechanistic insight into how transcriptional and chromatin programs shape the early transition of HSCs from youth to aging.

Previous studies have primarily linked Selp signaling to late-stage HSC aging and functional deterioration. De Haan et al demonstrated that signaling through P-selectin’s primary ligand, *Selplg*, modulates aging-associated transcriptional programs, with disruption of this pathway accelerating HSC aging (Yang et al, 2025). The transcriptional landscape of geriatric (22-month) HSCs in that study revealed a state defined by quiescence, erythroid repression, and broad myeloid bias. In contrast, our 14-month paHSCs reveal a clear molecular continuity that precedes this terminal state, with Selp^high^ HSCs in mid-life exhibiting a transcriptionally primed state of intracellular signaling pathways. This altered baseline signaling likely serves as a precursor to the chronic inflammatory phenotype and eventual lineage bias observed in geriatric hematopoiesis (Yang et al, 2025).

Similarly, Hu et al showed that chronic Selp elevation drives HSC proliferation, oxidative stress, and genomic instability via IFNγ-JAK1-STAT1 and PI3K–AKT-mTOR pathways (He et al, 2025). In contrast, our study focuses on functional heterogeneity during the early phases of HSC aging, demonstrating that reduced Selp expression marks an early-aged HSC subset with preserved metabolic fitness, intact redox homeostasis, and resistance to myeloid-biased transcriptional reprogramming, thereby defining an early protective state within the aging continuum. Notably, this early-stage functional heterogeneity is conserved across mice and humans, underscoring the translational relevance of Selp as both a marker and regulator of early HSC aging. It is also important to note that global transcriptional divergence does not necessarily preclude functional preservation. In Selp^low^ paHSCs, while the hierarchical separation from young controls might be driven by a unique adaptive signature, these cells maintain a core self-renewal program that remains characteristic of a more youthful state.

Furthermore, our ATAC-seq analysis revealed distinct upstream transcriptional networks governing Selp^high^ and Selp^low^ HSCs during early aging. Notably, Selp^low^ early-aged HSCs are enriched for PU.1 motifs, a pioneer transcription factor that opens lineage-specific enhancers and maintains immune readiness. By modulating cell cycle and self-renewal programs, PU.1 preserves quiescence and protects HSCs from stress-induced overexpansion and functional decline (Chavez et al, 2021; Staber et al, 2013b). PU.1 has also been implicated in regulating oxidative stress and mitochondrial function (Zhong et al, 2025); hence, the enrichment of PU.1 motifs in Selp^low^ cells suggests that it may shield this subset from oxidative damage, maintain metabolic stability, and sustain transcriptional fidelity, supporting the resilience of Selp^low^ HSCs during aging. Functional validation of these protective roles in vivo will be important in future studies and may reveal opportunities to modulate PU.1 activity to mitigate age-associated HSC dysfunction. In contrast, Selp^high^ early-aged HSCs are associated with CTCF, whose redistribution has been linked to chromatin disorganization, transcriptional drift, and altered three-dimensional genome architecture in aged HSCs (Tang et al, 2024). Particularly, in response to inflammatory signals, CTCF rapidly occupies increasingly accessible regulatory regions of myeloid genes in aged HSCs, potentially driving their preferential myeloid differentiation (Cui et al, 2025).

We further explored the relationship between SELP expression and CHIP, a condition characterized by the expansion of HSCs harboring somatic mutations in the absence of overt malignancy (Weeks and Ebert, 2023). Our single-cell transcriptomic profiling reveals that the relationship between *SELP* expression and CHIP progresses through a distinct, multi-stage molecular trajectory across lifespans. This evolution initiates during the middle-age transition with an early priming of core epigenetic regulators within the *SELP*_high population. As aging advances, this state shifts into a full-scale transcriptional divergence characterized by the peak expression of pro-inflammatory mediators and critical RNA splicing factors, alongside a prominent upregulation of p53 stress-associated transcripts.

Importantly, our human module score analysis in Fig. 6G demonstrates that while systemic aging elevates baseline inflammatory and metabolic stress pathways across the entire geriatric stem cell pool, the *SELP*_high subset undergoes an accelerated, multi-pathway collapse. These findings suggest that SELP serves as more than a passive marker of functional exhaustion. Instead, it acts as a highly effective risk-stratification tool that identifies a vulnerable HSC subset uniquely primed for clonal emergence. By bridging early middle-age epigenetic erosion with a late-stage hyper-inflammatory bone marrow environment, SELP expression reflects a state of chronic cellular stress that may facilitate the expansion of mutant clones, ultimately identifying individual stem cells at high risk for myeloid transformation.

Collectively, our work reveals that Selp delineates a spectrum of HSC states during the early phase of aging, from vulnerable Selp^high^ to resilient Selp^low^ cells, in both humans and mice. This insight provides a conceptual framework for early, biomarker-guided interventions aimed at preserving or rejuvenating hematopoietic function, highlighting therapeutic opportunities to prevent age-associated hematologic decline and to counteract inflammation-driven stem cell dysfunction before significant functional deterioration occurs.

## Methods

**Table:** Reagents and tools table

Reagent/resource	Reference or source	Identifier or catalog number
**Experimental models**		
C57BL/6-Ly5.2 (CD45.2)	Beijing Vital River Laboratory Animal Technology Co., Ltd	N/A
B6.SJL (CD45.1)	Animal facility of the State Key Laboratory of Experimental Hematology	N/A
**Antibodies**		
c-Kit	BioLegend; Invitrogen	105847; 404-1171-82
Sca-1	BioLegend	108126
CD4	BioLegend; Invitrogen	100540; 17-0041-83
CD8	BioLegend; Invitrogen	100706; 45-0081-82
B220	Biolegend	103243; 103236
TER-119	Biolegend	116228
Gr-1	BioLegend	108428; 108408
CD34	Invitrogen	11-0341-85
Mac-1	Biolegend	101228; 101236
Flt3	Biolegend; Invitrogen	135306; 17-1351-82
CD45.2	BioLegend	109808; 109824
CD45.1	BioLegend	110730
CD48	BioLegend; Invitrogen	103428; 11-0481-85
CD150	BioLegend	115914; 115929
P-selectin	Invitrogen	17-0626-80
Phospho-Histone H2A.X	Invitrogen	12-9865-42
Ki-67 Monoclonal Antibody	Invitrogen	12-5698-82
Rabbit anti-P-selectin/CD62P recombinant monoclonal antibody	Bethyl Laboratories	A700-268
Goat anti-Rabbit IgG (H + L) Alexa Fluor™ 488	Invitrogen	A-11008
**Oligonucleotides and other sequence-based reagents**		
qPCR primers	This study	Table EV2
**Chemicals, enzymes, and other reagents**		
lipopolysaccharide (LPS)	MedChemExpress	HY-D1056
MitoTracker™ Green FM	MedChemExpress	HY-135056
TMRE	Invitrogen	T669
CellROX	Invitrogen	C10443
MitoSOX	Invitrogen	M36005
Hoechst	Miltenyi Biotec	130-111-569
Propidium Iodide	Biolegend	421301
**Software**		
Prism 10	Graphpad Software	https://www.graphpad.com
Endnote 8.2	Clarivate	https://endnote.com/
FlowJo (v10.10.1)	FlowJo, LLC	https://www.flowjo.com/
Imaris (v10.2.0)	Oxford Instruments	https://imaris.oxinst.com/
Integrative Genomics Viewer (IGV; version 2.19.4)	UC San Diego and Broad Institute	https://igv.org/
RStudio Desktop (v2025.05.0)	Posit Software, PBC	https://posit.co/
Seurat (v5)	Satija Lab	https://satijalab.org/seurat/
Monocle3	Trapnell Lab	https://cole-trapnell-lab.github.io/monocle3/
ggplot2	Posit Software, PBC	https://ggplot2.tidyverse.org/
fastp (v0.20.0)	OpenGene	https://github.com/OpenGene/fastp
HISAT2 (v2.2.1)	Daehwan Kim Lab	https://daehwankimlab.github.io/hisat2/
featureCounts (v2.0.6)	Walter and Eliza Hall Institute of Medical Research	https://subread.sourceforge.net/
DESeq2 (v1.42.0)	Bioconductor	https://bioconductor.org/packages/DESeq2
clusterProfiler (v4.8.1)	Bioconductor	https://bioconductor.org/packages/clusterProfiler
GSEA	Broad Institute	https://www.gsea-msigdb.org/gsea/index.jsp
BWA (bwa mem v0.7.12)	Heng Li Lab	https://github.com/lh3/bwa
MACS2 (v2.1.0)	Xiaole Shirley Liu Lab	https://github.com/macs3-project/MACS
HOMER (v4.9.1)	Benner Lab	http://homer.ucsd.edu/homer/
**Other**		
RNeasy Mini Kit	Qiagen	74104
SMARTer® Ultra® Low RNA Kit for Illumina®	Takara Bio/Clontech	634936
TruePrep DNA Library Prep Kit V2 for Illumina	Vazyme	TD503-02
Hyperactive ATAC-Seq Library Prep Kit for Illumina	Vazyme	TD711
TruePrep Index Kit V2 for Illumina	Vazyme	TD202
IntraPrep Permeabilization Reagent	Beckman Coulter	IM2389
ABScript Neo RT Master Mix for qPCR with gDNA Remover	ABclonal	RK20433
2× Universal SYBR Green Fast qPCR Mix	ABclonal	RK21203

### Mice

All mice were in a C57BL/6 background. Detailed strain and source information are provided in the Reagents and tools table. To eliminate sex-specific data variability, only age-matched female mice were used. Mice were kept within a specific pathogen-free (SPF) facility maintained at 22 ± 2 °C and 50–70% humidity under a 12-h light/dark cycle. The investigators were not blinded during the experiments. All mice were maintained under experimental protocols approved by the institutional animal care and use committees of the State Key Laboratory of Experimental Hematology (IHCAMS-DWLL-NSFC2024120-2) and complied with the Chinese national standard GB/T 35892-2018.

### Flow cytometry, cell sorting, and analysis

For HSC analysis, bone marrow (BM) cells were harvested, subjected to red blood cell lysis, and incubated with Fc receptor-blocking antibody prior to surface staining. Cells were subsequently stained with fluorophore-conjugated antibodies against surface markers and sorted or analyzed by flow cytometry using BD FACSAria™ III Cell Sorter and FACSymphony™ A1 Cell Analyzer (BD Biosciences). Detailed information regarding the fluorophore-conjugated antibodies used for FACS analysis is provided in the Reagents and tools table.

### Bone marrow transplantation assays

Competitive bone marrow transplantation assays were performed to assess the repopulating capacity of distinct HSC populations. BM cells from CD45.2 donor mice were subjected to fluorescence-activated cell sorting to isolate ~1000 HSCs per recipient. Sorted donor HSCs were mixed with 5 × 10^5^ whole BM competitor cells isolated from CD45.1 mice and intravenously injected into lethally irradiated CD45.1 recipient mice. Recipient mice received a split dose of 9 Gy with 4-h intervals prior to transplantation. Peripheral blood was collected from transplanted recipients at 4-, 8-, 12-, and 16-week post-transplantation to evaluate donor-derived hematopoietic reconstitution. At the terminal time point (16 weeks), BM was harvested for further analysis. Donor chimerism and lineage output were determined by flow cytometry based on CD45.1/CD45.2 expression.

### Single-cell RNA-sequencing analysis

Raw gene-cell count matrices from publicly available single-cell RNA-sequencing datasets were imported into Seurat (v5) (Hao et al, 2024). Standard quality-control filtering was applied to remove low-quality cells, including those with high mitochondrial transcript percentages, low gene counts, or potential doublets. Canonical correlation analysis (CCA) was performed using the standard Seurat integration workflow. Integration features were identified using SelectIntegrationFeatures, followed by anchor detection using FindIntegrationAnchors. To identify enriched biological pathways, differentially expressed genes (DEGs) were identified via FindMarkers and were subsequently subjected to Kyoto Encyclopedia of Genes and Genomes (KEGG) pathway enrichment analysis using clusterProfiler. Trajectory reconstruction was performed using Monocle (Cao et al, 2019; Qiu et al, 2017; Trapnell et al, 2014). The Seurat object was converted to a Monocle cell_data_set object, and the integrated UMAP coordinates were mapped into Monocle for graph learning and pseudotime ordering. Trajectory states were mapped back to Seurat clusters for interpretation. All visualizations were generated using Seurat, Monocle3, and ggplot2.

### Bulk RNA-sequencing and analysis

Total RNA was isolated using the RNeasy Mini Kit according to the manufacturer’s instructions. cDNA was synthesized using the SMARTer® Ultra® Low RNA Kit for Illumina® following the manufacturer’s instructions. Libraries were constructed using the TruePrep DNA Library Prep Kit V2 for Illumina following the manufacturer’s protocols. Raw reads were processed with fastp to remove adapters, low-quality reads, and poly-N sequences. Clean reads were aligned to the reference genome using HISAT2 (v2.2.1), and gene expression was quantified using featureCounts (v2.0.6) with FPKM normalization.

### Differential expression analysis

Differential gene expression between two groups was performed using the DESeq2 R package (v1.42.0) (Love et al, 2014), which models read counts with a negative binomial distribution. *P* values were adjusted for multiple testing using the Benjamini–Hochberg method, and genes with adjusted *P* ≤ 0.05 and |log₂(fold change)| ≥1 were considered significantly differentially expressed.

### GO and KEGG enrichment analysis

Gene Ontology (GO) enrichment analysis was performed using the clusterProfiler (v4.8.1) with correction for gene length bias. GO terms with adjusted *P* < 0.05 were considered significantly enriched. KEGG pathway enrichment was also analyzed using clusterProfiler, assessing whether differentially expressed genes were significantly enriched in known KEGG pathways (http://www.genome.jp/kegg/).

### Gene set enrichment analysis (GSEA)

Genes were ranked by differential expression, and enrichment of gene sets was assessed at the top or bottom of the ranked list. Both GO and KEGG datasets were used independently for enrichment analysis via the local GSEA tool.

### ATAC-seq library construction

Chromatin accessibility was assessed using the Hyperactive ATAC-Seq Library Prep Kit for Illumina, and indexed libraries were prepared with the TruePrep Index Kit V2 for Illumina following the manufacturer’s instructions. Libraries were subsequently sequenced on the Illumina X Plus platform (Novogene).

### ATAC sequencing and analysis

Indexed ATAC-seq libraries were clustered on a cBot Cluster Generation System using the TruSeq PE Cluster Kit v3-cBot-HS (Illumina) and sequenced on the Illumina NovaSeq platform to generate 150 bp paired-end reads. Raw reads were processed with fastp (v0.20.0) to remove adapters, poly-N sequences, low-quality reads (> 40% bases with quality <15), and reads shorter than 18 bp. Clean reads were aligned to the reference genome using BWA mem (v0.7.12), discarding reads from mitochondria/chloroplasts, improperly paired reads, and PCR duplicates, and retaining only uniquely mapped reads with MAPQ ≥ 13. Peaks were called using MACS2 (v2.1.0) (Zhang et al, 2008) with a q-value threshold of 0.05. Motif discovery was performed on 500 bp regions centered on peak summits using HOMER (v4.9.1) (Heinz et al, 2010). Peak annotation and assignment to nearest genes were performed with ChIPseeker (Yu et al, 2015), followed by KEGG pathway analysis using KOBAS. Differential peaks between groups were identified by merging peaks with bedtools, calculating mean RPM, and selecting peaks with |log2 fold change | >1. Normalized tracks were generated for visual comparison using the Integrative Genomics Viewer (IGV; version 2.19.4).

### Lipopolysaccharide (LPS)-induced acute inflammatory stress model

To induce acute systemic inflammatory stress, mice were administered lipopolysaccharide (LPS) on Day 0. LPS derived from *Escherichia coli* O55:B5 was dissolved in sterile phosphate-buffered saline (PBS) and injected intraperitoneally at a dose of 0.5 mg/kg body weight.

### Mitochondrial and oxidative stress assays

MitoTracker™ Green FM was used to assess mitochondrial mass, while TMRE was applied to evaluate mitochondrial membrane potential. CellROX™ was used to detect cytosolic ROS, and MitoSOX™ was used to measure mitochondrial ROS. Cells were subjected to mitochondrial and oxidative stress assays according to the manufacturer’s instructions.

### Intracellular phospho-H2A.X and Ki-67 staining

For the assessment of DNA damage and cell-cycle status, cells were fixed and permeabilized using the IntraPrep Permeabilization Reagent according to the manufacturer’s instructions. Permeabilized cells were subsequently stained with Phospho-Histone H2A.X (Ser139) monoclonal antibody or Ki-67 monoclonal antibody at the recommended working dilutions. For cell-cycle analysis, Hoechst 33342 was added following intracellular staining to label DNA content. After washing, samples were acquired and analyzed by flow cytometry.

### Confocal microscopy

Cells were fixed with 4% paraformaldehyde and permeabilized with 0.1% Triton X-100. Fixed and permeabilized cells were incubated overnight at 4 °C with Rabbit anti-P-selectin/CD62P recombinant monoclonal antibody. After washing, cells were incubated with a fluorophore-conjugated secondary antibody. Confocal images were acquired using a Dragonfly 200 high-speed spinning disk confocal microscope (Andor Technology) and rendered using Imaris software (v10.2.0).

### RNA extraction and quantitative real-time PCR (qPCR)

RNA extraction was performed as described previously (Yang et al, 2024). Reverse transcription was carried out using ABScript Neo RT Master Mix for qPCR with gDNA Remover, according to the manufacturer’s instructions. Quantitative PCR (qPCR) was performed using 2× Universal SYBR Green Fast qPCR Mix. Primer sequences used for qPCR are listed in Table EV2.

### Statistical analysis

All experiments were performed at least three times under similar conditions. Statistical analyses were performed using GraphPad Prism. Comparisons between two groups were assessed using Student’s *t* test (two-tailed). For multi-group and time-course analyses, statistical significance was evaluated using two-way repeated measures ANOVA followed by appropriate multiple comparisons tests, as indicated in the corresponding figure legends. Data are presented as mean ± standard deviation (SD). *P* < 0.05 was considered statistically significant.

### Graphics

The synopsis and select experimental illustration elements were created with BioRender.com.

## Supplementary information

Table EV1

Table EV2

Peer Review File

Source data Fig. 2

Source data Fig. 4

Source data Fig. 5

Expanded View Figures

## Data Availability

Bulk RNA-seq raw and processed data generated in this study have been deposited in the Gene Expression Omnibus (GEO) under accession number GSE319049. Bulk ATAC-seq raw and processed data generated in this study have been deposited in the Gene Expression Omnibus (GEO) under accession number GSE319303. The processed single-cell RNA sequencing data for human CD34⁺ hematopoietic stem and progenitor cells (HSPCs) were obtained from and re-analyzed using a publicly available dataset in the Gene Expression Omnibus (GEO) under accession number GSE189161. The source data of this paper are collected in the following database record: biostudies:S-SCDT-10_1038-S44318-026-00887-w.
