# Supplementary material for: P-selectin delineates conserved functional heterogeneity in early hematopoietic stem cell aging in humans and mice
Source: EMBO J. 2026 Aug 5;45(18):6421–49. doi: 10.1038/s44318-026-00887-w (PMC13578734; doi:10.1038/s44318-026-00887-w)
Supplement: Supplementary file 1 [file 44318_2026_887_MOESM1_ESM.pdf]

Table EV1. List of genes used to generate gene module scores.

Module	Gene Symbols
**Megakaryocyte (MK)**	*TPO, MPL, ITGA2B, ITGB3, PF4, MYH9, RUNX1, NFE2, VWF, PFN1, CXCR4, PRKG1, DGKA, GNA13, PIK3CG, P2RX1, CSRP1, VCL, THBS1, ITGB1*
**Myeloid**	*IL1B, NFKB1, IRF5, NLRP3, STAT3, CEBPA, LYZ, MPO, STAT1, LY86, CD74, CD80, CXCL8, S100A9, CD33, CD68, CCR2, IL6, TNF, FCGR3A, CCL3, MMP9, CXCL10, CD163, FCGR1A, CD14, S100A8, CSF1R, CCL2, CX3CR1*
**Lymphoid**	*CD3D, CD3E, CD19, CD79A, CD79B, IL7R, BCL6, TCF7, LEF1, BLNK, LCK, ZAP70, PRF1, NKG7, GZMB, IFNG, IL2RG, IKZF1, CD52, CD27*
**Inflammation**	*IL1B, IL6, TNF, NFKB1, NFKBIA, IL18, NLRP3, PYCARD, CXCL8, CCL2, CCL5, CXCL10, TLR2, TLR4, MYD88, STAT3, JUN, FOS, PTGS2, NOS2, NOS3, SMAD3, SMAD2, SMAD7, IRF3, IRF7, MAPK1, MAPK8, IKBKB, CXCL1, IL12A, RELA*
**Mitochondrial**	*NDUFA1, NDUFA10, NDUFA11, NDUFA3, NDUFA5, NDUFA6, NDUFA7, NDUFA8, NDUFB9, NDUFAB1, NDUFAF1, NDUFAF2, NDUFAF6, NDUFB2, NDUFB4, NDUFB6, NDUFS1, NDUFS2, NDUFS3, NDUFS4, NDUFS5, NDUFS7, NDUFS8, NDUFV1, NDUFV3, SDHA, SDHB, SDHC, SDHD, COX4I1, COX5A, COX5B, COX6A2, COX6B1, COX7A1, COX7A2, COX7B, COX14, ATP4A, ATP5B, ATP5C1, ATP5D, ATP5E, ATP5G1, ATP7B, ATP5O, ATP5L, ATP8B1, ATP10A, ATP7A, TFAM, PPARGC1A, TIMM17A, TIMM17B, TIMM22, TIMM23, TIMM50, TOMM20, TOMM22, TOMM40*
**Redox**	*GPX2, SOD2, GLRX5, GPX1, SOD1, PRDX3, TXNRD1, TXN, CAT, TXNL1, NFE2L2, GLRX2, PRDX4, NOX1, PRDX6, NOX5, PRDX5, MGST1, GLRX3, GPX8, PIK3CD, PTPN11, AHR, PIK3R1, MAPK3, FOXO3, MGST3, MAPK14, RELA, KEAP1*
