# Supplementary material for: P-selectin delineates conserved functional heterogeneity in early hematopoietic stem cell aging in humans and mice
Source: EMBO J. 2026 Aug 5;45(18):6421–49. doi: 10.1038/s44318-026-00887-w (PMC13578734; doi:10.1038/s44318-026-00887-w)
Supplement: Supplementary file 2 [file 44318_2026_887_MOESM2_ESM.pdf]

Table EV2. List of qPCR primer used in the study

Selp-F	CTCATGAACTGCAGCCATCC	*Selp*-R	TCCCAGAAGCCAAACATTGC
Clu-F	CTCCGAGCTTCCACAACATG	*Clu*-R	TGCGGTCATCTTCACCTTCT
Vwf-F	GGTTTTCTCTCCCTGGCTCT	*Vwf*-R	CAACTGCTGGATGGATCTGC
Gfi1-F	AGAATGAACTCTGGGCAGCT	*Gfi1*-R	TAAATGCATCAGTGGCGCTC
Timp3-F	CCCTTTGGCACTCTGGTCTA	*Timp3*-R	ACGTGGGGCATCTTACTGAA
Ccl6-F	AACTCTACCCTGGCACTTCC	*Ccl6*-R	TGCTCACTCCTCCCTGATTC
Ctcf-F	GCTGCCTTTGTCTGTTCCAA	*Ctcf*-R	TGTCTCCCCTCCATTTTCCC
Ctcfl-F	AGAGGACCCACAAGAACGAG	*Ctcfl*-R	CTTCTCTCCTGTGTGCATGC
Spi1-F	ACTTCACAGAGCTGCAGAGT	*Spi1*-R	GCATCTGTTCCAGCTCCATG
Fli1-F	GTCACCTCCTCCAGCTTCTT	*Fli1*-R	GGGACACTGGGGTTTGGATA
Actb-F	GTGGGAATGGGTCAGAAGGA	*Actb*-R	CTTCTCCATGTCGTCCCAGT
