# Supplementary material for: P-selectin delineates conserved functional heterogeneity in early hematopoietic stem cell aging in humans and mice
Source: EMBO J. 2026 Aug 5;45(18):6421–49. doi: 10.1038/s44318-026-00887-w (PMC13578734; doi:10.1038/s44318-026-00887-w)
Supplement: Supplementary file 3 [file 44318_2026_887_MOESM3_ESM.pdf]

THE EMBO JOURNAL

P-selectin delineates conserved functional heterogeneity in early hematopoietic stem cell aging in humans and mice

Chong Yang, Tong Wang, Yue Chai, Lisha Wang, Yixuan Wang, Weili Wang, Fang Dong, and Toshio Suda

Corresponding author: Toshio Suda (sudato@keio.jp)

Review Timeline:

Submission Date:	12th Feb 26
Editorial Decision:	7th Apr 26
Revision Received:	10th Jun 26
Editorial Decision:	1st Jul 26
Revision Received:	7th Jul 26
Accepted:	23rd Jul 26

Editor: Daniel Klimmeck

Transaction Report:

(Note: With the exception of the correction of typographical or spelling errors that could be a source of ambiguity, letters and reports are not edited. Depending on transfer agreements, referee reports obtained elsewhere may or may not be included in this compilation. Referee reports are anonymous unless the Referee chooses to sign their reports.)

Dear Toshio,

Thank you again for the submission of your manuscript (EMBOJ-2026-123859) to The EMBO Journal, and providing us with a preliminary point-by-point response to the concerns raised by the referees. As mentioned, your study was assessed by two reviewers with expertise in aging in the blood system and hematopoietic stem cell biology, whose comments are enclosed below.

As you will see from their comments, the referees acknowledge the timeliness and potential appeal of your findings. However, they also express major concerns regarding the robustness and conclusiveness of the analysis provided with respect to stress dependence of the annotated aged HSC chromatin landscapes and integration with previous related results (Ref#1, pts. 1,2) and rigor of in vivo exploration of Selp status dependence of HSC functionality (Ref#2, pts.1,4; see also Ref#1, pt.3). Further, they request substantial amendments to methods annotation and orthogonal validation, and raise a number of issues related to the presentation of the findings, clarity of the data presentation and plausibility of the claims from the data shown, that would need to be conclusively addressed to achieve the level of robustness and clarity needed for The EMBO Journal.

Given the overall interest stated and broader angle of your results, we are able to invite you to revise your manuscript experimentally to address the referees' comments, along the lines sketched in your outline. I need to stress though that we do require strong support from the referees on a revised version of the study in order to move on to publication of the work.

Please feel free to contact me if you have any questions or need further input on the referee comments.

As you know, we generally allow three months as standard revision time. As a matter of policy, competing manuscripts published during this period will not negatively impact on our assessment of the conceptual advance presented by your study. However, we request that you contact the editor as soon as possible upon publication of any related work, to discuss how to proceed. Should you foresee a problem in meeting this three-month deadline, please let us know in advance and we may be able to grant an extension.

When preparing your letter of response to the referees' comments, please bear in mind that this will form part of the Review Process File, and will therefore be available online to the community. For more details on our Transparent Editorial Process, please review our Editorial Policies: https://link.springer.com/partners/embo-press/editorial-policies

When submitting your revised manuscript, please carefully review the instructions below.

Please feel free to approach me any time should you have additional questions related to this.

Thank you for the opportunity to consider your work for publication.

I look forward to your revision.

Best regards,

Daniel

Daniel Klimmeck, PhD
Senior Editor
The EMBO Journal

Instruction for the preparation of your revised manuscript:

Read our guidance for manuscript revisions and related editorial policies: https://link.springer.com/journal/44318/submission-guidelines#cms-Revised-submissions
1) a .docx formatted version of the manuscript text (including legends for main figures, EV figures and tables). Please make sure that the changes are highlighted to be clearly visible.

2) individual production quality figure files as .eps, .tif, .jpg (one file per figure).

3) a .docx formatted letter INCLUDING the reviewers' reports and your detailed point-by-point response to their comments. As part of the EMBO Press transparent editorial process, the point-by-point response is part of the Review Process File (RPF), which will be published alongside your paper.

4) a complete author checklist, which you can download from our author guidelines. Please insert information in the checklist that is also reflected in the manuscript. The completed author checklist will also be part of the RPF.

5) Please note that all corresponding authors are required to supply an ORCID ID for their name upon submission of a revised manuscript.

6) It is mandatory to include a 'Data Availability' section after the Materials and Methods. Before submitting your revision, primary datasets produced in this study need to be deposited in an appropriate public database, and the accession numbers and database listed under 'Data Availability'. Please remember to provide a reviewer password if the datasets are not yet public.
In case you have no data that requires deposition in a public database, please state so in this section. Note that the Data Availability Section is restricted to new primary data that are part of this study.
*** Note - All links should resolve to a page where the data can be accessed. ***

7) Our journal encourages inclusion of *data citations in the reference list* to directly cite datasets that were re-used and obtained from public databases. Data citations in the article text are distinct from normal bibliographical citations and should directly link to the database records from which the data can be accessed. In the main text, data citations are formatted as follows: "Data ref: Smith et al, 2001" or "Data ref: NCBI Sequence Read Archive PRJNA342805, 2017". In the Reference list, data citations must be labeled with "[DATASET]". A data reference must provide the database name, accession number/identifiers and a resolvable link to the landing page from which the data can be accessed at the end of the reference.

8) At EMBO Press we ask authors to provide source data for the main and EV figures. Our source data coordinator will contact you to discuss which figure panels we would need source data for and will also provide you with helpful tips on how to upload and organize the files.

Numerical data can be provided as individual .xls or .csv files (including a tab describing the data). For 'blots' or microscopy, uncropped images should be submitted (using a zip archive or a single pdf per main figure if multiple images need to be supplied for one panel.

9) We replaced Supplementary Information with Expanded View (EV) Figures and Tables that are collapsible/expandable online. A maximum of 5 EV Figures can be typeset. EV Figures should be cited as 'Figure EV1, Figure EV2" etc. in the text and their respective legends should be included in the main text after the legends of regular figures.

- For the figures that you do NOT wish to display as Expanded View figures, they should be bundled together with their legends in a single PDF file called *Appendix*, which should start with a short Table of Content. Appendix figures should be referred to in the main text as: "Appendix Figure S1, Appendix Figure S2" etc.

- Additional Tables/Datasets should be labelled and referred to as Table EV1, Dataset EV1, etc. Legends have to be provided in a separate tab in case of .xls files. Alternatively, the legend can be supplied as a separate text file (README) and zipped together with the Table/Dataset file.

10) When assembling figures, please refer to our figure preparation guideline in order to ensure proper formatting and readability in print as well as on screen:
https://media.springernature.com/original/springer-cms/rest/v1/content/27825798/data/v1

Please remember: Digital image enhancement is acceptable practice, as long as it accurately represents the original data and conforms to community standards. If a figure has been subjected to significant electronic manipulation, this must be noted in the figure legend or in the 'Materials and Methods' section. The editors reserve the right to request original versions of figures and the original images that were used to assemble the figure.

11) For data quantification: please specify the name of the statistical test used to generate error bars and P values, the number

(n) of independent experiments (specify technical or biological replicates) underlying each data point and the test used to calculate p-values in each figure legend. The figure legends should contain a basic description of n, P and the test applied. Graphs must include a description of the bars and the error bars (s.d., s.e.m.).

Please remember: Digital image enhancement is acceptable practice, as long as it accurately represents the original data and conforms to community standards. If a figure has been subjected to significant electronic manipulation, this must be noted in the figure legend or in the 'Materials and Methods' section. The editors reserve the right to request original versions of figures and the original images that were used to assemble the figure.

Further information is available in our Guide For Authors: https://link.springer.com/journal/44318/submission-guidelines#cms-Revised-submissions

Please remember: Digital image enhancement is acceptable practice, as long as it accurately represents the original data and conforms to community standards. If a figure has been subjected to significant electronic manipulation, this must be noted in the figure legend or in the 'Materials and Methods' section. The editors reserve the right to request original versions of figures and the original images that were used to assemble the figure.

We realize that it is difficult to revise to a specific deadline. In the interest of protecting the conceptual advance provided by the work, we recommend a revision within 3 months (12th Mar 2026). Please discuss the revision progress ahead of this time with the editor if you require more time to complete the revisions.

Referee #1:

Although P-selectin has previously been identified as a molecule that is preferentially expressed on aged murine HSCs, little is known on how expression alters during development and throughout life, and little to nothing is known on the relevance of P-selectin expression on human HSCs.

In this manuscript the authors present convincing data on the relevance of SELP expression on human HSCs, and in addition provide a novel mechanism by which Selp may affect (murine) HSC dysfunctioning as they demonstrate that upon Selp induction (after LPS) exposure, mitochondrial membrane potential and various ROS species increase. These data, in conjunction with data that have recently been published by others, demonstrate that Selp is not merely a marker for aged HSCs, but that Selp signalling contributes to functional activity of HSCs.

Overall, the experiments have been carefully executed and are well presented. There are several issues that the authors may want to address, which would further strengthen their observations.

1. Recently, a paper was published in which murine Selp-hi and Selp-low HSC subsets were transcriptionally profiled (ref 16 in the current manuscript). It would be useful to assess to what extent the transcriptomes that the authors present in the present manuscript overlap (or not) with those reported in ref 16.
2. The authors identify chromatin changes in Selp-hi and Selp-lo HSCs, including opening up of the locus that encodes Selp. These changes have likely gradually accumulated in HSCs as they age. The authors also show that transcriptional changes can rapidly be induced by exposing HSCs to LPS. Do the authors have data (or could they speculate) on how and whether the epigenetic chromatin landscape rapidly changes (within 24 hrs) after HSCs have been challenged by LPS?
3. It would be interesting to know whether in aged human HSCs, SELP and SELPLG are expressed by the same individual HSCs? The data shown in Figure 1F and G should allow the authors to assess this quite easily. If expressed by the same cells, would this suggest autocrine signalling?
4. The data in figure 1 originate from a recently published single cell RNA-Seq analysis. It would be useful to indicate in the text, or legend of the figure, how human HSCs were sorted. What markers were used?
5. In figure 2A I would change 'SLAM" into "CD150". There are more SLAM markers than only CD150, so it seems more insightful to indicate that in these 2 panels CD150 expression is displayed.

Referee #2:

Yang et al nicely establish that an upregulated transcript in aged HSCs, Selp, often reported in murine studies, can be used to define a population of myeloid biased HSCs that can be detected middle age and also in humans. This marker to distinguish myeloid/meg biased human HSC is an important finding. Unfortunately, the studies performed in the murine model to evaluate the differences between Selphi and Selplow could have been more robustly performed, and there are some concerns about the interpretation of some of the data.

Major concerns:

1) Transplantation is the gold standard for HSC transplants, and from young WT animal, 1000 HSCs against 5 X 10^5 WBM competitor cells should have extremely robust overall reconstitution compared to the ~20% PB chimerism- it is possible that the 9.0 GY or radiation did not robustly deplete the endogenous HSCs, but this very low chimerism for 1000 young donor HSCs. Additionally, these transplants from 3 groups had only 3 recipients per donor group- making these data (without individual data points presented) difficult to fully evaluate. How many donor mice were used to collect the 1000 donor HSCs- were they pooled? Please show individual data points in the fig 2g and 2i. What was the wbm chimerism in these transplanted mice? Within the donor derived cells, were there differences in the frequency of the HSCs from the CD45.2 LSK cells?

2) For RNA seq analysis in Fig 3, please show a PCA of the RNA-seq replicates between the young, paSelphi and paSelplow HSC and clarify the number of replicates to generate the RNA-seq analysis. The paSelphi HSCs seem reminiscent of the VWF or CD41high HSCs- and these genes are enriched in the transcripts of the paSelphi DEG- is there heterogeneity between the Selphi compared to VWF high or CD41 high HSCs? What did Neo1 levels look like as this is another gene expression that correlates with HSC lineage skewing.

3) After LPS injection, the authors evaluate the Selphi fraction. However, it is unclear if the LSK compartment was used to identify these cells as myeloid progenitors upregulate Sca1 expression under LPS stimulation and myeloid progenitors- especially MkPs express Selp and may be inflating the increased frequency of cells seen in the Selphi fraction.

4) The authors propose that the Selphi fraction of HSCs are present at mid-age and have restricted lineage output and changes in proinflammatory and stress signals - likely maintained in old age- while the Selplow fraction is a more functionally robust population. At 20 months (fig 2C), there is a large frequency of Selplow HSC- do these cells maintain their functional robustness at older ages? If not, at what point would Selp expression no longer be a useful marker for separating these functionally different HSCs? This would be particularly relevant for the human samples, as one of the key findings of this manuscript is the definition of a cell surface marker on human HSCs that may be able to identify myeloid-biased HSCs from more balanced HSCs, and thus if there is a limitation on what aged groups this would be useful for, this should be discussed.

5) The ATAC data, do paSelplow HSC compared to young HSCs show any of the same enrichments for CTCF or CTCFL as the paSelphi vs paSelplow- is there a gradient of open chromatin enrichment with Selp expression (even when low compared to non-expressed?)

6) Touching again on the use of SELP in human samples, would it be possible to determine if Selphi HSC were enriched for clonal hematopoiesis mutations? Could the expression of Selp help identify CHIP/ are there potential implications for CHIP?

Minor question- in fig 4C, 4 replicates are shown while in all other data points from these experiments, there were only 3 replicates- please clarify if these data are generated from different animals

Responses to Reviewers

Referee #1:

Although P-selectin has previously been identified as a molecule that is preferentially expressed on aged murine HSCs, little is known on how expression alters during development and throughout life, and little to nothing is known on the relevance of P-selectin expression on human HSCs.
In this manuscript the authors present convincing data on the relevance of SELP expression on human HSCs, and in addition provide a novel mechanism by which Selp may affect (murine) HSC dysfunctioning as they demonstrate that upon Selp induction (after LPS) exposure, mitochondrial membrane potential and various ROS species increase. These data, in conjunction with data that have recently been published by others, demonstrate that Selp is not merely a marker for aged HSCs, but that Selp signalling contributes to functional activity of HSCs.
Overall, the experiments have been carefully executed and are well presented. There are several issues that the authors may want to address, which would further strengthen their observations.

1. Recently, a paper was published in which murine Selp-hi and Selp-low HSC subsets were transcriptionally profiled (ref 16 in the current manuscript). It would be useful to assess to what extent the transcriptomes that the authors present in the present manuscript overlap (or not) with those reported in ref 16.

Response: We appreciate this constructive suggestion. We agree that a direct comparison with the transcriptional profiles reported by Yang et al. (2025) provides critical context for our characterization of $Selp^{high}$ HSCs. A key distinction of our study is the focus on pre-aged HSCs (14–16 months), whereas Yang et al. analyzed geriatric HSCs (>22 months). This temporal difference allows us to identify molecular signatures that serve as early drivers of lineage skewing versus those that represent the consequences of advanced aging.

As shown in the table below, our $Selp^{high}$ paHSCs exhibit a transcriptional program that heavily overlaps with the geriatric subsets, specifically through the enrichment of "Platelet activation" and "Regulation of actin cytoskeleton". While Yang et al. emphasize a broad myeloid bias and the repression of erythroid master regulators like Gata1, our data highlights that the megakaryocyte/platelet-priming component of this bias is already firmly instantiated by the pre-aged stage. Furthermore, the transition from the JAK-STAT/PI3K-Akt signaling we observe at 14 months to the innate immune activation reported at 22 months suggests a progression where HSCs first become hyper-responsive to external cues before settling into a state of chronic

inflammatory dysfunction.

Feature	Yang et al., Nature Aging, 2025	Present Manuscript	Biological Interpretation
**Mouse Age**	>22 months (Geriatric)	14 months (Early-aged)	Our data captures the initiation of the aging phenotype.
**Selp^{high} Profile**	• Myeloid biased;<br>• Erythroid repression;<br>• Quiescent.	• Myeloid/Platelet biased;<br>• JAK-STAT signaling.	Conserved: Platelet priming and niche-adhesion wiring are early events in HSC aging.
**Selp^{low} Profile**	Lymphoid differentiation	• Hematopoietic cell lineage;<br>• lymphoid signaling;<br>• Antigen presentation.	Conserved: Selp^{low} remains a robust marker for lymphoid-poised HSCs across age groups.
**Signaling Axis**	Innate immune activation.	PI3K-Akt and JAK-STAT signaling.	Transition: 14m cells show high cytokine-responsive signaling, potentially preceding the "inflamm-aging" seen at 22m.

We have updated the Discussion section to include this comparative analysis, which reinforces Selp as a consistent marker of the myeloid-biased HSC lineage throughout the aging process.

2. The authors identify chromatin changes in Selp-hi and Selp-lo HSCs, including opening up of the locus that encodes Selp. These changes have likely gradually accumulated in HSCs as they age. The authors also show that transcriptional changes can rapidly be induced by exposing HSCs to LPS. Do the authors have data (or could they speculate) on how and whether the epigenetic chromatin landscape rapidly changes (within 24 hrs) after HSCs have been challenged by LPS?

Response: We thank the reviewer for this insightful point regarding the kinetics of chromatin remodeling. While we have not directly measured chromatin accessibility in HSCs within 24 hours of LPS exposure, we agree that the relationship between age-associated remodeling and acute inflammatory response is important to our model.

The literature establishes that a 24-hour window is robustly sufficient for epigenetic rewiring in HSCs. While acute LPS stimulation induces only transient transcriptomic and phenotypic shifts, it leaves a persistent imprint on chromatin accessibility at specific myeloid lineage enhancers (de Laval et al., 2020). Mechanistically, extracellular signals rapidly couple to signal-dependent

transcription factors (Glass & Natoli, 2016). In HSCs, a well-characterized driver of this immediate inflammatory response is the transcription factor C/EBPβ, which targets closed chromatin to deposit stable epigenetic marks (de Laval et al., 2020). This rapid, C/EBPβ-dependent remodeling may place critical genes into a permanently poised state, establishing a structural memory. Hence, these studies demonstrate a conserved molecular axis wherein a 24-hour inflammatory window is sufficient for C/EBPβ-mediated chromatin rewriting.

3. It would be interesting to know whether in aged human HSCs, SELP and SELPLG are expressed by the same individual HSCs? The data shown in Figure 1F and G should allow the authors to assess this quite easily. If expressed by the same cells, would this suggest autocrine signalling?

Response: We thank the reviewer for this insightful question. We examined the single-cell expression of SELP and SELPLG within the aged human HSC compartment. While both genes are expressed within the same overall HSC population, co-expression at the single-cell level is relatively limited, with the majority of HSCs expressing either SELP or SELPLG alone. Only a small subset of cells shows detectable co-expression.

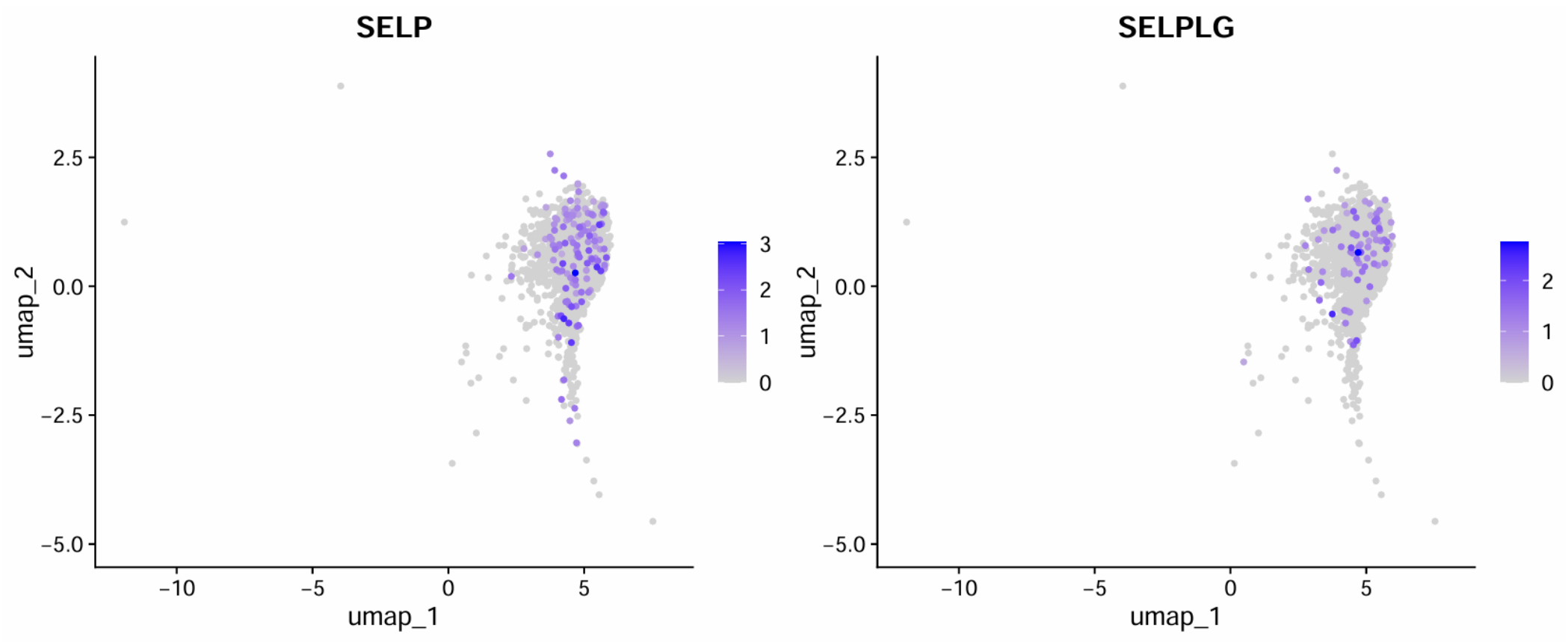

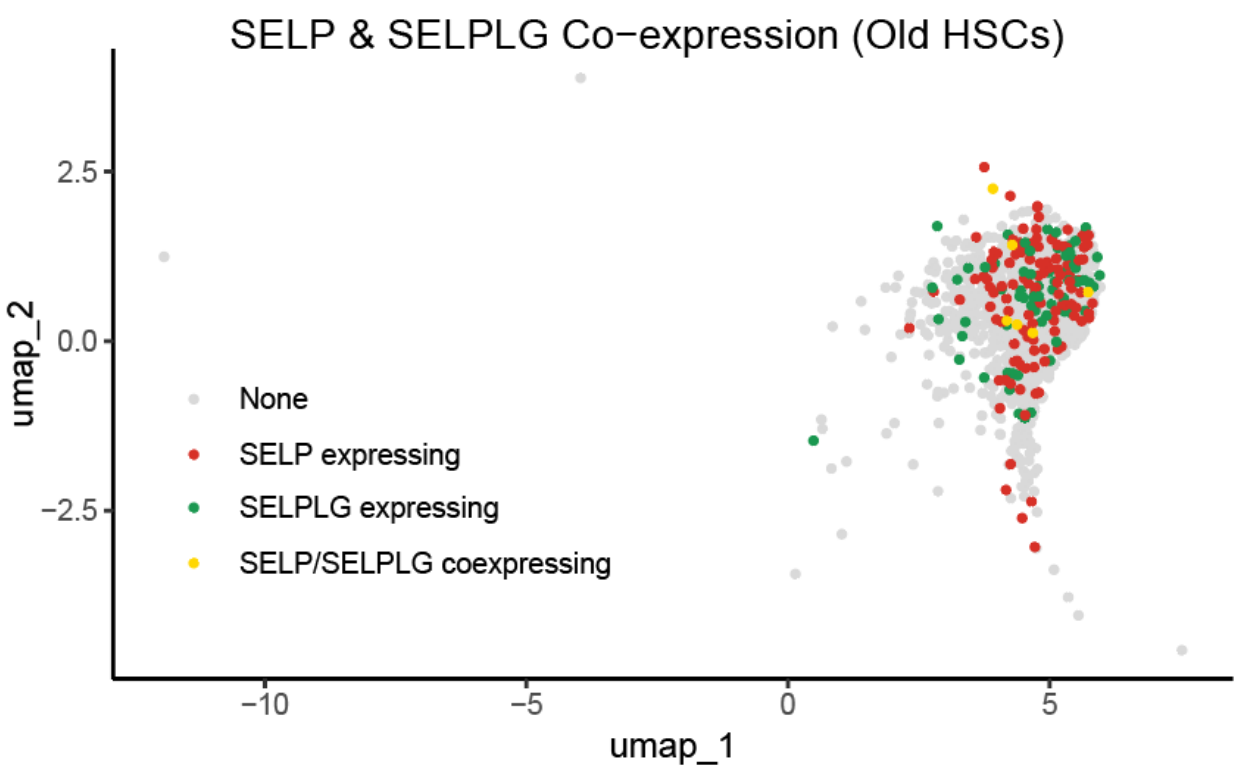

These findings suggest that *SELP* and *SELPLG* are not broadly co-regulated within individual HSCs, but rather reflect heterogeneous expression states within the population. Importantly, although co-expression could in principle permit cell-autonomous interactions, the limited

overlap observed here does not strongly support a predominant autocrine signaling mechanism. Instead, this pattern is more consistent with context-dependent or cell-cell interaction roles.

4. The data in figure 1 originate from a recently published single cell RNA-Seq analysis. It would be useful to indicate in the text, or legend of the figure, how human HSCs were sorted. What markers were used?

Response: We thank the reviewer for this suggestion. We have updated the figure legend to explicitly state that the annotation of the human HSPC data was derived from a previously published single-cell RNA-seq dataset. We would like to clarify that we did not perform de novo cell type annotation for this analysis. Instead, we utilized the original author-provided annotations available in the associated public metadata.

To ensure the robustness of these inherited labels for our specific study, we verified the expression of canonical lineage-specific markers and HSC signature (*HLF*) in Fig. EV1. For comprehensive details regarding the original sorting strategies and gating hierarchies used to define these populations, we refer the reader to the primary publication cited in the text.

5. In figure 2A I would change 'SLAM" into "CD150". There are more SLAM markers than only CD150, so it seems more insightful to indicate that in these 2 panels CD150 expression is displayed.

Response: We thank the reviewer for this correction. We have replaced “SLAM” with “CD150” in Figure 2A for clarity.

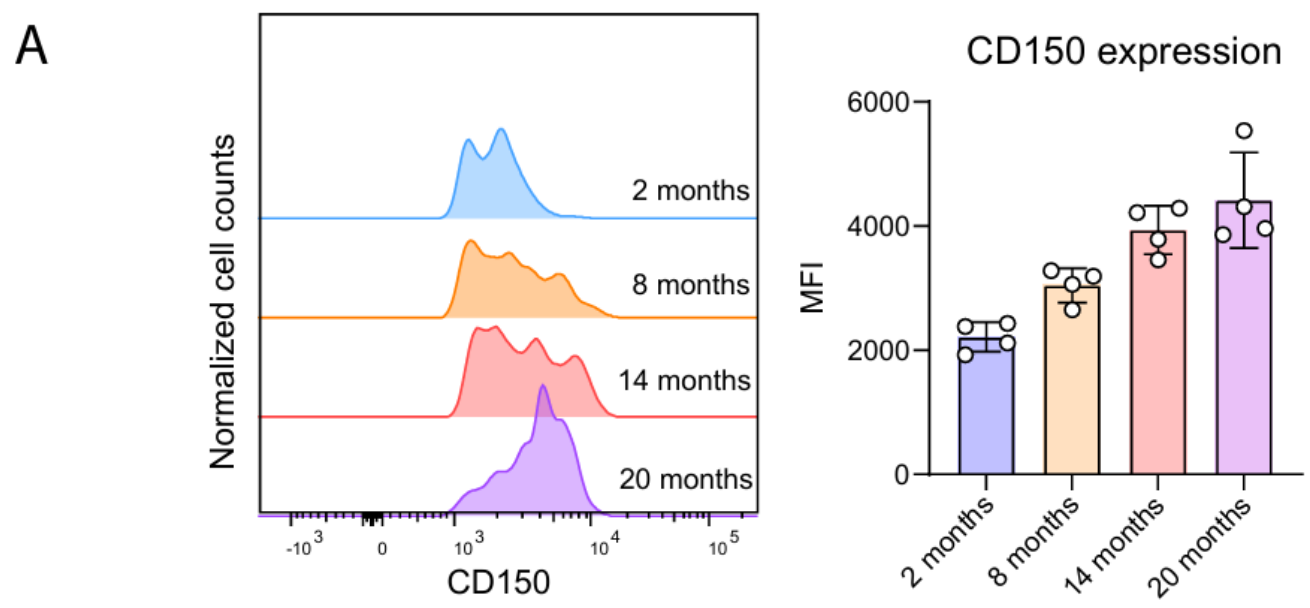

Referee #2:

Yang et al nicely establish that an upregulated transcript in aged HSCs, Selp, often reported in murine studies, can be used to define a population of myeloid biased HSCs that can be detected middle age and also in humans. This marker to distinguish myeloid/meg biased human HSC is an important finding. Unfortunately, the studies performed in the murine model to evaluate the differences between Selphi and Selplow could have been more robustly performed, and there are some concerns about the interpretation of some of the data.

Major concerns:

1) Transplantation is the gold standard for HSC transplants, and from young WT animal, 1000 HSCs against 5 X 10^5 WBM competitor cells should have extremely robust overall reconstitution compared to the ~20% PB chimerism- it is possible that the 9.0 GY or radiation did not robustly deplete the endogenous HSCs, but this very low chimerism for 1000 young donor HSCs. Additionally, these transplants from 3 groups had only 3 recipients per donor group- making these data (without individual data points presented) difficult to fully evaluate. How many donor mice were used to collect the 1000 donor HSCs- were they pooled? Please show individual data points in the fig 2g and 2i. What was the wbm chimerism in these transplanted mice? Within the donor derived cells, were there differences in the frequency of the HSCs from the CD45.2 LSK cells?

Responses: We thank the reviewer for the opportunity to clarify our transplantation protocol and the resulting chimerism levels. Regarding the irradiation dose, we noted a clerical error in the initial manuscript. Following our relocation to the current institute, the protocol was adjusted to a split-dose of 9 Gy (4.5 Gy × 2, 4h apart) to ensure robust myeloablation of endogenous HSCs while mitigating gastrointestinal toxicity and acute radiation-induced stress. We have updated the Materials and Methods section to reflect this accurately.

As the reviewer noted, 1000 HSCs against 5 x 10^5 competitor cells is a significant donor pool. However, we interpret the observed ~25% chimerism as a reflection of the high stringency of our competitive model and the specific seeding efficiency of our experimental setup. While absolute chimerism can vary between facilities based on sorting parameters and recipient housing, the final output in a competitive assay is a product of both the donor-to-competitor ratio and the initial homing kinetics following high-purity FACS isolation.The mechanical and oxidative pressures of multi-parameter sorting can influence immediate seeding efficiency without compromising long-term identity. Specifically, the rarity of Selp-defined HSC fractions in aged mice necessitates prolonged sorting durations. This extended *ex vivo* period can impact immediate homing and seeding kinetics. Crucially, to ensure a strictly controlled experiment, young control HSCs (yHSCs) were held *ex vivo* for the same duration and processed under

identical conditions alongside the aged fractions.

Because all cohorts including the control yHSCs and Selp fractions were subjected to these identical technical constraints, the ~25% chimerism in the control group serves as a reliable, internally consistent baseline. The significant and reproducible functional decline observed in the Selp^{high} fraction remains the primary evidence for Selp as a marker of functional exhaustion. This relative difference remains statistically robust and biologically relevant within this controlled competitive environment.

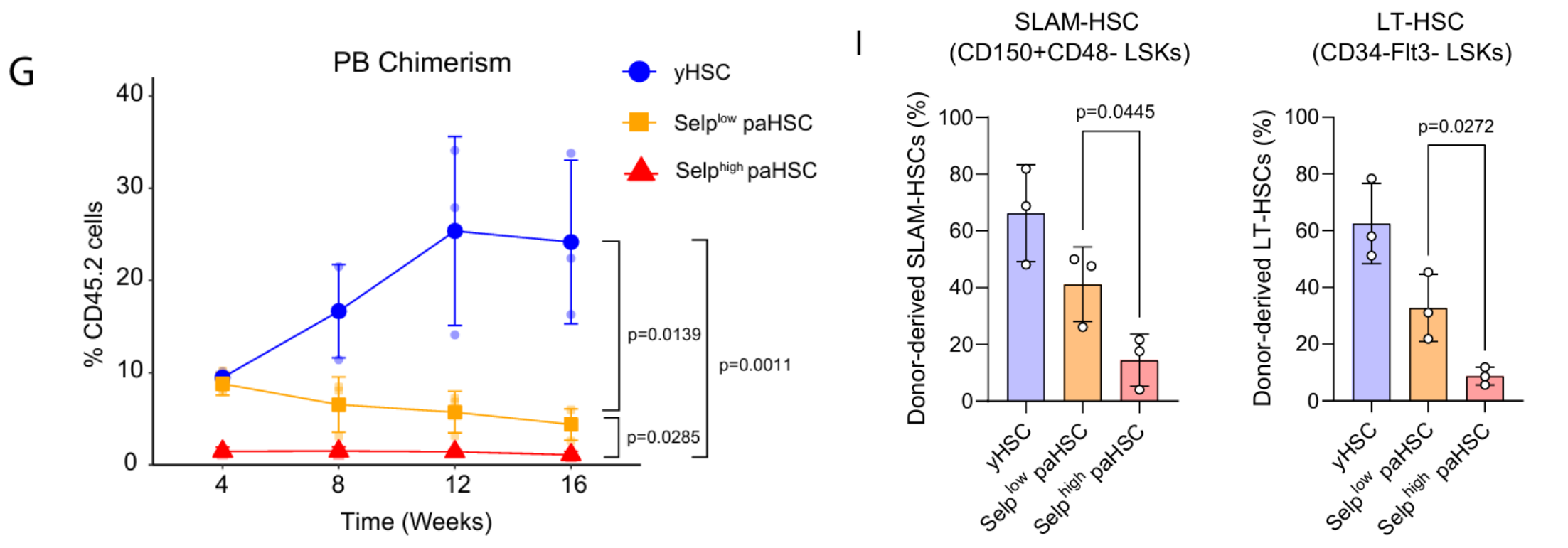

We have updated Figures 2G and 2I to show individual data points for each recipient mouse. To ensure longitudinal consistency, the gating strategy was stringently verified across all time points. Furthermore, error bars in Figure 2G were modified from SEM to SD to better represent biological variance. This refined analysis fully sustains our original conclusions with higher precision.

Due to the inherent rarity of the Selp^{high} HSC fraction in aged mice, donor HSCs were pooled from three age-matched donor mice per group, as it ensures a sufficient cell dose while capturing a representative biological average. While we acknowledge that n=3 recipients per group represents a focused cohort, the strikingly consistent functional gradient observed across the Selp^{low} and Selp^{high} groups provide strong confidence in the biological phenomenon described. The clear step-down in reconstitution capacity across these three populations demonstrates that Selp expression is a robust and sensitive marker of functional decline in the aged hematopoietic system.

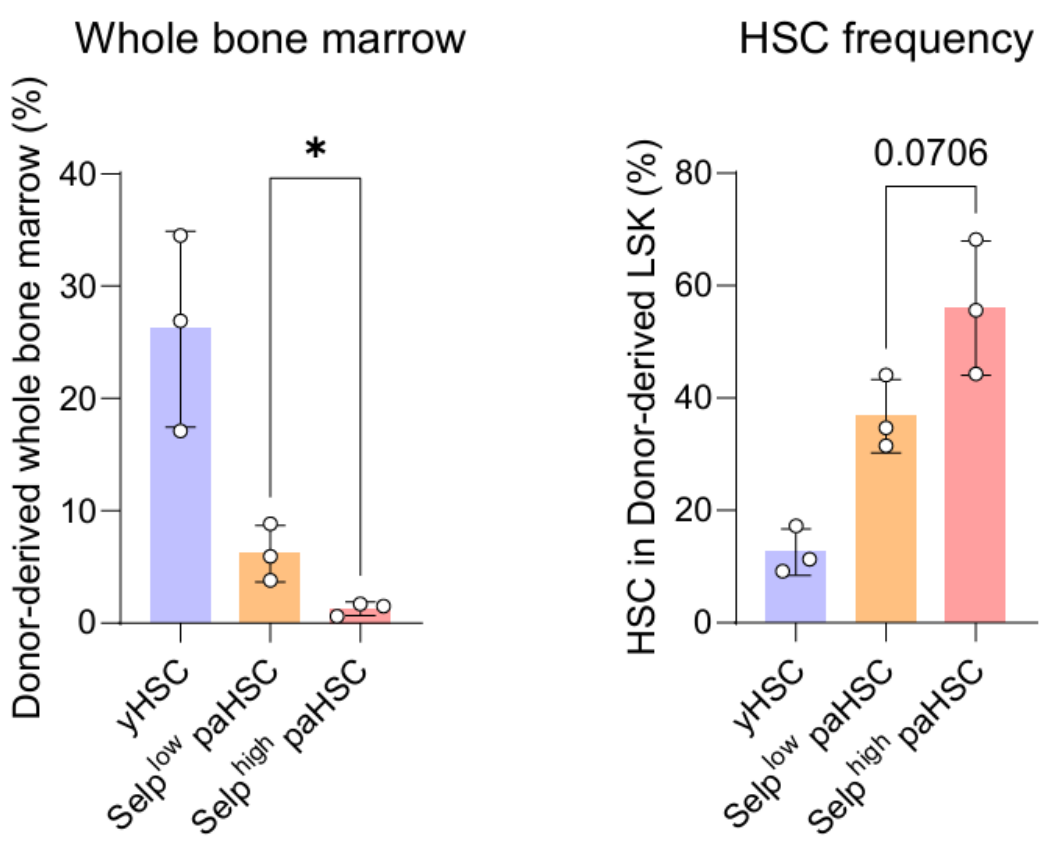

We also thank the reviewer for comments on detailed bone marrow analysis. We have updated the figures to include Whole Bone Marrow (WBM) chimerism and the frequency of phenotypic HSCs within the donor-derived compartment.

1. Whole Bone Marrow (WBM) Chimerism at 16 Weeks

The WBM chimerism mirrors the trends observed in the peripheral blood, confirming that the peripheral readouts accurately reflect the bone marrow state.

2. HSC Frequency within Donor-Derived LSK Cells

To assess the "quality" of the engrafted cells, we analyzed the frequency of phenotypic HSCs (CD150+ CD48- LSK) within the donor-derived (CD45.2+) LSK population. We observed a paradoxical trend that is a well-characterized hallmark of HSC aging: (1). Young HSCs exhibited a balanced hematopoietic hierarchy, where a relatively small pool of stem cells maintains robust downstream output. (2). $Selp^{low}$ and $Selp^{high}$ paHSCs: Showed a progressive increase in phenotypic HSC frequency. This Paradoxical Increase in the $Selp^{high}$ fraction suggests a profound differentiation block or phenotypic expansion without functional output. While these aged cells retain the surface markers of a stem cell, they fail to contribute effectively to the progenitor pools. This indicates that the $Selp^{high}$ fraction is functionally exhausted and trapped in a primitive state, providing a clear mechanistic explanation for the failure of these cells to maintain peripheral blood production over 16 weeks.

E

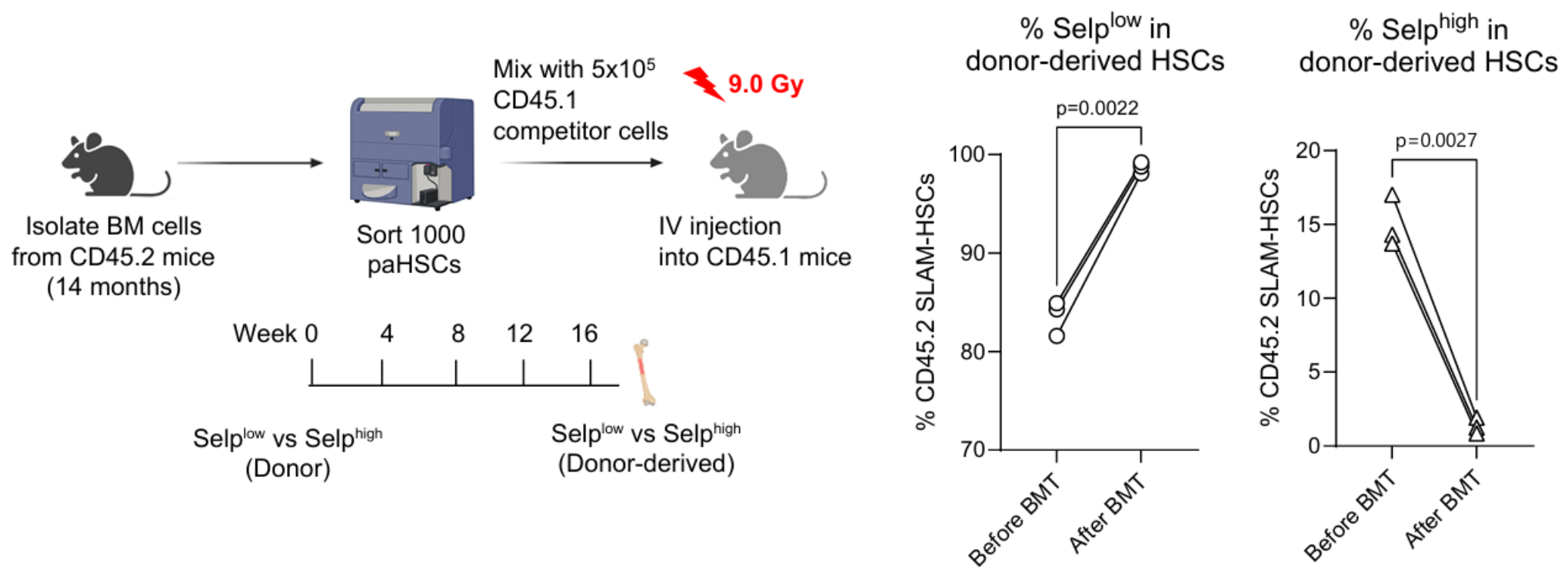

Furthermore, as part of our original experimental design to evaluate functional exhaustion, we performed a prospective transplantation experiment using purified SLAM-HSCs from 14-month-old donors prior to the submission of this study. Our analysis of the donor-derived compartment 16 weeks post-transplant reveals a striking loss of phenotypic identity in the Selp^{high} fraction, with nearly all donor-derived HSCs residing in the Selp^{low} gate. Conversely, the Selp^{low} fraction robustly reconstituted the HSC pool while maintaining its specific phenotypic profile. This one-way transition indicates that Selp^{high} HSCs are not only functionally inferior in terms of blood output, but also lack the self-renewal capacity required to maintain their subset identity in a competitive setting. These findings identify Selp^{high} HSCs as a terminal, exhausted state, providing a clear mechanistic basis for the differentiation block and impaired fitness described in our manuscript. We have integrated the data into Figure EV2 in the revised manuscript.

2) For RNA seq analysis in Fig 3, please show a PCA of the RNA-seq replicates between the young, paSelphi and paSelplow HSC and clarify the number of replicates to generate the RNA-seq analysis. The paSelphi HSCs seem reminiscent of the VWF or CD41high HSCs- and these genes are enriched in the transcripts of the paSelphi DEG- is there heterogeneity between the Selphi compared to VWF high or CD41 high HSCs? What did Neo1 levels look like as this is another gene expression that correlates with HSC lineage skewing.

Response: We thank the reviewer for the comments. For all groups in Figure 3, we utilized 3 biological replicates, representing a total of 9 independent samples. To ensure high-fidelity profiling of these rare populations, cDNA was synthesized using the SMARTer Ultra Low RNA Kit. We have provided a hierarchical clustering heatmap of all 9 replicates in Figure EV3 as we believe this offers a more comprehensive view of the data structure and sample correlation than a standard PCA. As shown in the dendrogram, the yHSC and Selp^{high} paHSC groups cluster with high uniformity, confirming the robustness of our low-input workflow. While the Selp^{low} paHSC group exhibits greater transcriptomic drift, we interpret this as a valid biological state reflecting the broader transcriptional landscape of the Selp^{low} fraction compared to the more programmed state of Selp^{high} cells.

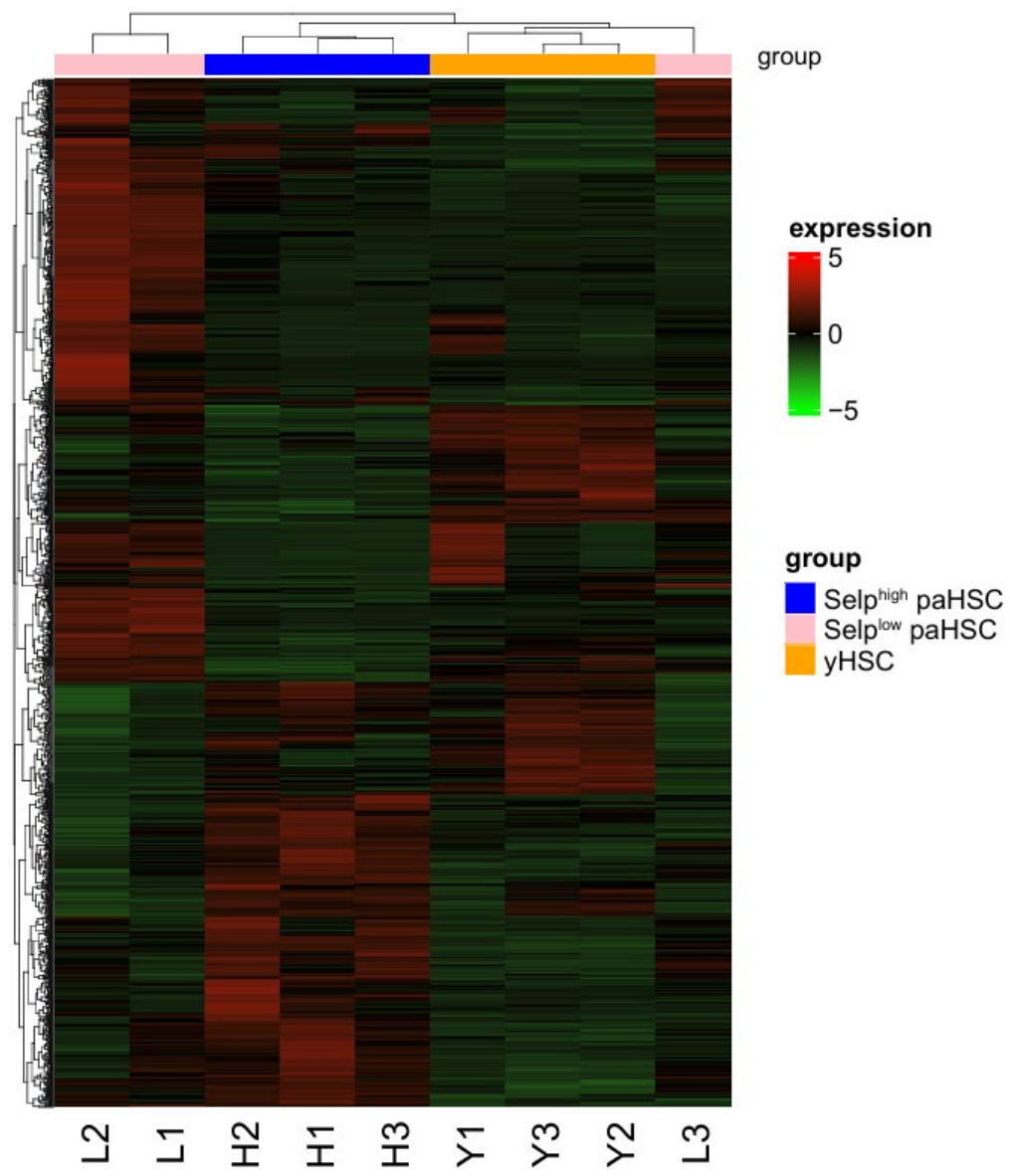

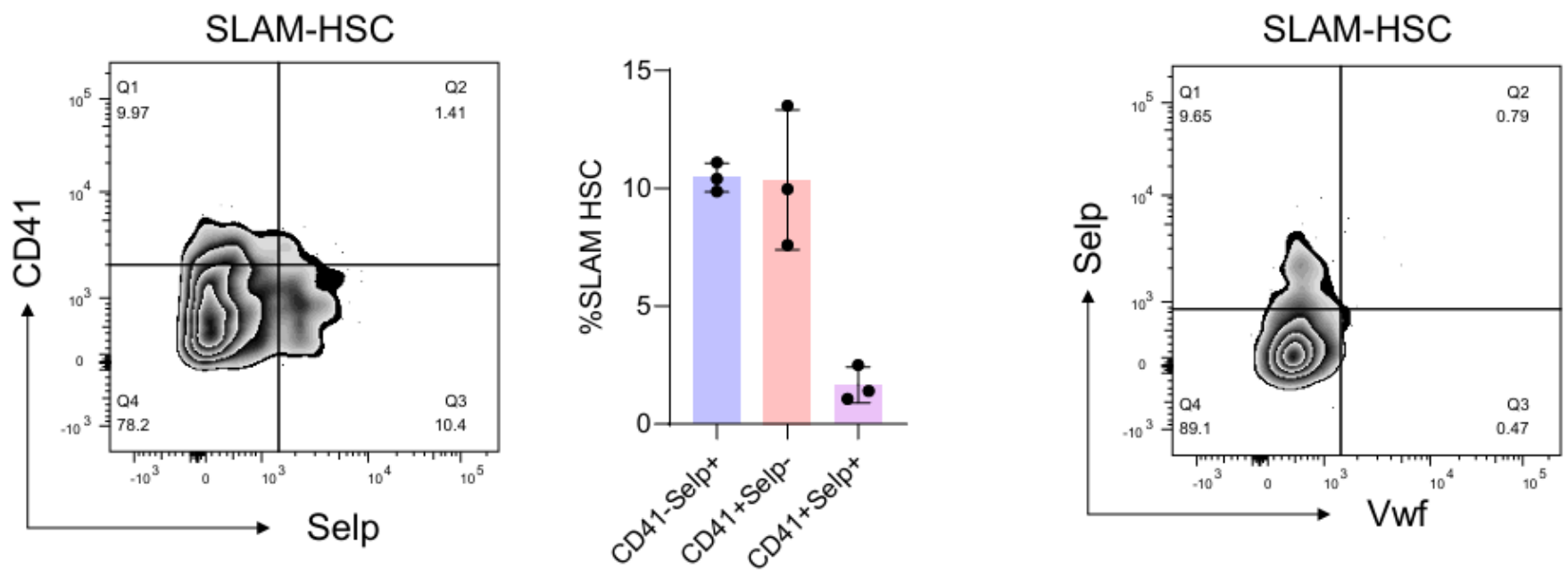

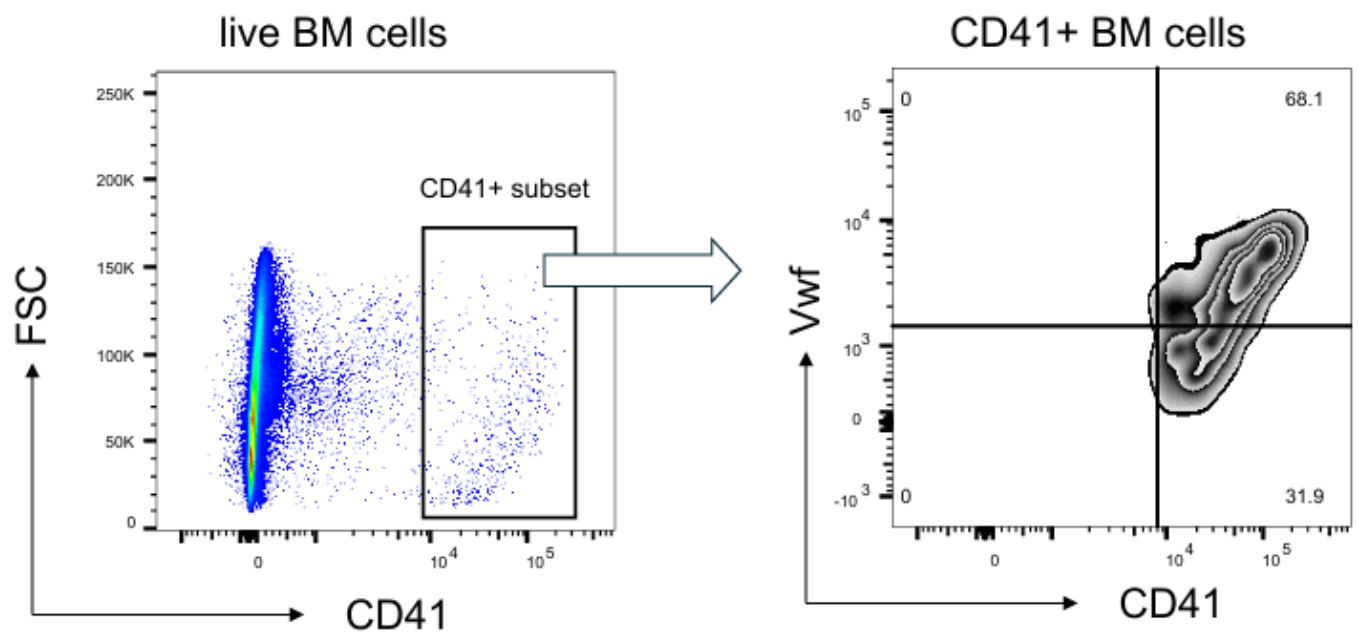

To address the reviewer's comment regarding the overlap between the Selp^{high} population and established platelet-biased HSC subsets, we performed multi-color flow cytometry combining surface SLAM markers, Selp, CD41, and optimized intracellular staining for Vwf in early aged mice. Our empirical data demonstrate that despite sharing an upstream megakaryocyte-primed transcriptional signature, the Selp^{high} population represents a phenotypically distinct cellular entity, and vast heterogeneity exists in the early aged HSCs. Furthermore, the Selp^{high} population segregates from Vwf+ cells, with Vwf+ HSCs constituting a minor fraction of the total SLAM-HSC pool. We acknowledge that the frequency of Vwf+ HSCs detected via our

intracellular staining protocol is more conservative than frequencies reported using transgenic reporter mice. This is expected given the different detection thresholds of direct protein staining versus genetic reporters. To rule out any technical artifacts during intracellular processing, we utilized CD41+ bone marrow cells as an internal positive control, which yielded a robust and bright Vwf signal in over sixty-eight percent of the cells. Taken together, these results show that while the Selp^{high} paHSCs share molecular priming features with previously reported platelet-biased HSCs, they represent a physically distinct reservoir, which highlights the immense cellular heterogeneity of early aged HSCs, and warrants further detailed characterization in future studies.

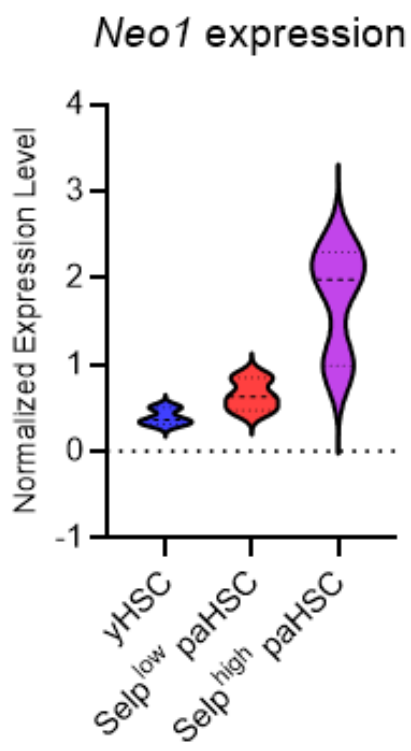

The reviewer also raises an excellent point regarding the relationship between SELP and known markers of lineage skewing, such as *Neo1*. Our transcriptomic analysis confirms a striking correlation: *Neo1* expression follows a clear upward gradient from Bulk Young to Selp^{low} paHSC, peaking in the Selp^{high} paHSC fraction. While the Selp^{high} paHSC population significantly upregulates *Neo1*, which is consistent with its myeloid-biased and pro-inflammatory phenotype, the Selp^{low} paHSC population maintains significantly lower *Neo1* levels, more closely resembling the youthful state.

3) After LPS injection, the authors evaluate the Selphi fraction. However, it is unclear if the LSK compartment was used to identify these cells as myeloid progenitors upregulate Sca1 expression under LPS stimulation and myeloid progenitors- especially MkPs express Selp and may be inflating the increased frequency of cells seen in the Selphi fraction.

Response: We thank the reviewer for the important point regarding potential Sca1 upregulation and the contamination of the Selp high fraction by activated myeloid or especially megakaryocyte progenitors under LPS stimulation. To address this, we employed a stringent gating strategy that moves beyond the LSK compartment to maintain high population purity. As demonstrated in the gating hierarchy (Fig. EV2B), our definition of hematopoietic stem cells relies on the SLAM markers CD150 positive and CD48 negative.

To specifically minimize the confounding effects of Sca-1 upregulation after LPS induction, we

identified MkPs by gating on the broader c-Kit positive population rather than the Sca-1-dependent LSK compartment illustrated below. By applying this approach and specifically gating on the CD48 negative subset, we effectively exclude the majority of CD41 positive MkPs that would otherwise inflate the Selp high fraction. This back-gating confirms that MkPs represent only a small fraction of the total SLAM-HSC gate even after LPS induction, ensuring that the observed shifts in Selp frequency are intrinsic to the purified stem cell pool.

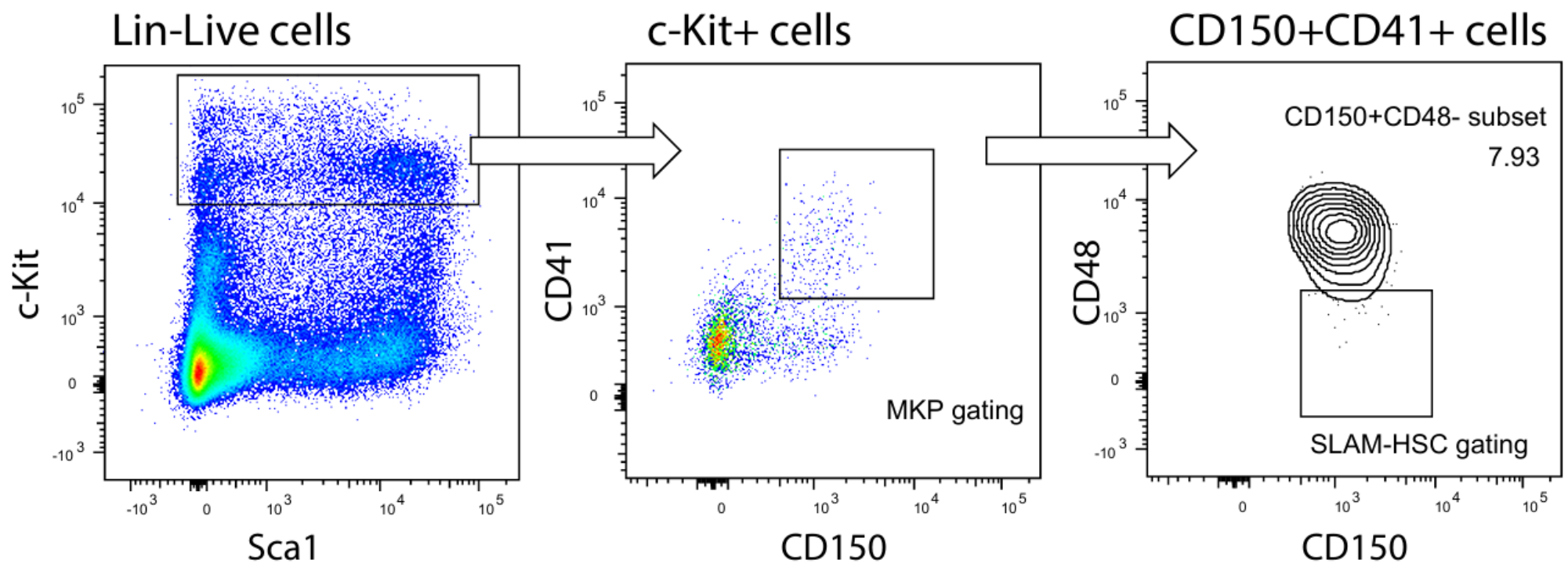

4) The authors propose that the Selphi fraction of HSCs are present at mid-age and have restricted lineage output and changes in proinflammatory and stress signals - likely maintained in old age- while the Selplow fraction is a more functionally robust population. At 20 months (fig 2C), there is a large frequency of Selplow HSC- do these cells maintain their functional robustness at older ages? If not, at what point would Selp expression no longer be a useful marker for separating these functionally different HSCs? This would be particularly relevant for the human samples, as one of the key findings of this manuscript is the definition of a cell surface marker on human HSCs that may be able to identify myeloid-biased HSCs from more balanced HSCs, and thus if there is a limitation on what aged groups this would be useful for, this should be discussed.

Response: We thank the reviewer for this profound point regarding the longitudinal utility of SELP as a biomarker across lifespans. The question of whether SELP_low HSCs maintain their youthful robustness into advanced old age is critical for defining the clinical window of our findings.

Our human single-cell module score analysis in Figure 6G demonstrates that human HSC aging follows a multi-stage trajectory. At middle age, the SELP_high versus SELP_low populations display largely overlapping distributions across inflammatory, myeloid, and redox module scores, indicating that middle age represents the baseline initiation phase of these stress programs rather than an absolute split. The functional divergence becomes more significant in advanced age, where the Old_SELP_high fraction undergoes a much more severe, accelerated collapse. Crucially, our data shows that SELP_low HSCs in old age do not remain

perfectly insulated from aging, as their inflammation, myeloid priming, and redox scores are visibly elevated relative to the young baseline. Therefore, SELP does not identify pristine youthful cells in elderly individuals. Instead, it serves as a highly effective discriminative marker in advanced age by successfully isolating a relatively preserved, less-damaged reservoir (SELP_low) from a highly compromised, hyper-inflammatory, and megakaryocytic/myeloid-skewed subset (SELP_high).

In the revised manuscript, we have expanded the Discussion to address the functional window:

1. Middle-Age Onset: We explicitly discuss that during the human middle-age transition, SELP marks the molecular initiation zone where cellular stress begins to brew, though significant phenotypic divergence is not yet fully locked in.
2. Advanced Age Stratification: We clarify that in geriatric cohorts, systemic aging affects the entire HSC pool, as evidenced by the baseline stress increases in the Old_SELP_low fraction. However, SELP remains highly valuable through advanced age because the divergence between the two fractions widens significantly, driven by the profound multi-pathway collapse of the SELP_high population.
3. Refined Clinical Application: We discuss the translation to human medicine, framing SELP not as an absolute tool for tracking perfectly youthful cells, but as a risk-stratification marker. Monitoring the ratio of these fractions across age cohorts could help identify patients undergoing accelerated hematological aging, early-onset myeloid bias, or priming towards Clonal Hematopoiesis of Indeterminate Potential (CHIP).

5) The ATAC data, do paSelplow HSC compared to young HSCs show any of the same enrichments for CTCF or CTCFL as the paSelphi vs paSelplow- is there a gradient of open chromatin enrichment with Selp expression (even when low compared to non-expressed?)

Response: We thank the reviewer for this insightful question regarding the epigenetic landscape. To address this, we would like to point to our motif enrichment analysis in Figure EV4C and shown below, which demonstrates a step-wise progression of chromatin accessibility that correlates with SELP expression levels.

C

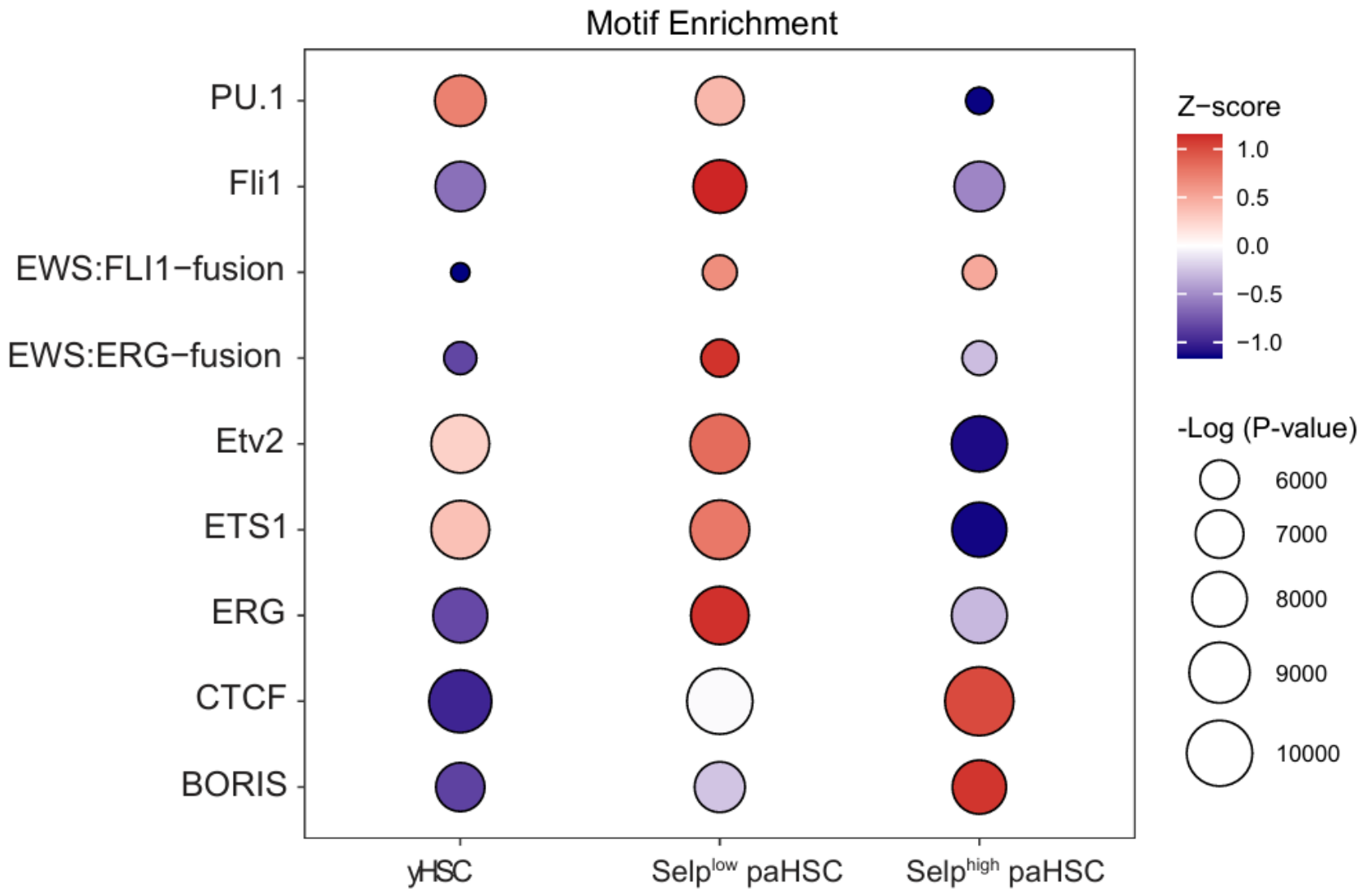

CTCF and BORIS (CTCFL) Gradient: As shown in the bubble plot, there is a clear directional shift in the Z-scores for CTCF and BORIS motifs. While yHSCs show the lowest accessibility (blue), the Selp^{low} paHSC population shows an intermediate state (white/light purple), and the Selp^{high} paHSC population shows the strongest enrichment (red). This confirms that even low Selp expression identifies a population that has begun the epigenetic transition toward the advanced aging state seen in Selp^{high} cells. This further validates SELP as a high-fidelity surface marker for tracking the progressive epigenetic "drift" of HSCs during the aging process. We have updated the Results and Discussion sections to specifically highlight this gradient of motif enrichment.

6) Touching again on the use of SELP in human samples, would it be possible to determine if Selphi HSC were enriched for clonal hematopoiesis mutations? Could the expression of Selp help identify CHIP/ are there potential implications for CHIP?

Response: We thank the reviewer for this insightful and clinically relevant suggestion. While we do not have matched somatic mutation data for the human HSC samples, we have performed an extensive single-cell analysis to evaluate whether SELP_high HSCs are enriched for the transcriptional signatures associated with CHIP-driver mutations and selective clonal fitness.

D

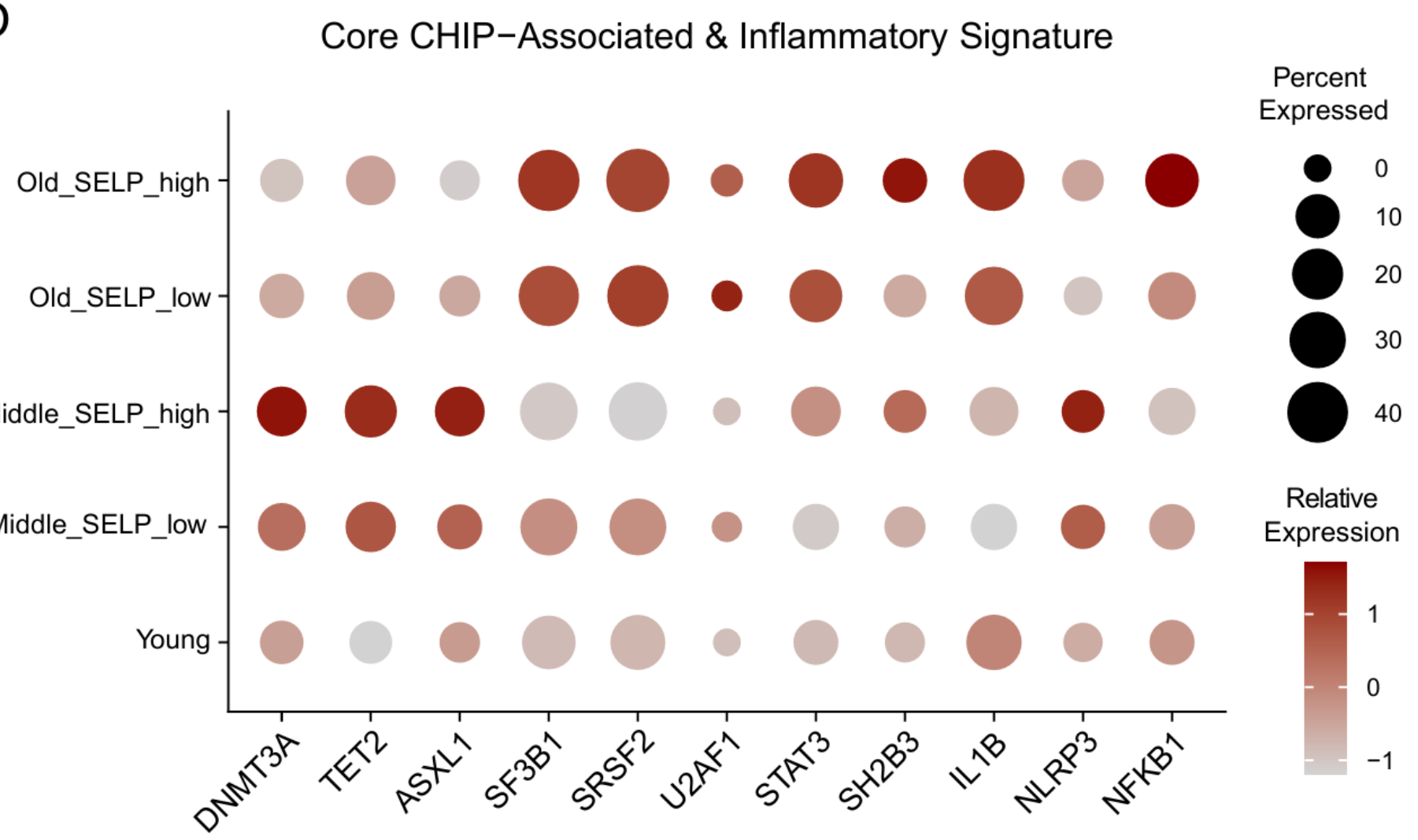

Our analysis reveals a biphasic molecular evolution of the SELP-high population across the human lifespan:

Early Epigenetic Instability (Middle-Age Transition): In middle-aged individuals, the SELP_high fraction exhibits a significant spike in the expression of the three primary epigenetic regulators of CHIP: ***DNMT3A, TET2***, and ***ASXL1***. This suggests that SELP expression identifies an early window of epigenetic erosion where the ground is prepared for clonal emergence.

Late-Stage Splicing and Inflammatory Priming (Old Age): As cells transition into the Old SELP_high group, the signature shifts toward advanced aging pathologies. These cells show peak expression of splicing machinery genes (***SF3B1, SRSF2, U2AF1***) and pro-inflammatory signaling mediators (***IL1B, NFKB1, STAT3***). This environment of chronic inflamm-aging is a known requirement for the selective expansion of mutant clones over their wild-type neighbors.

Hence, we propose that SELP expression serves as a high-fidelity cell-surface marker for a vulnerable HSC subset. These cells are transcriptionally primed for clonal expansion through a combination of early epigenetic instability and late-stage inflammatory stress.

We have included these new findings in Fig EV5D and expanded our discussion to propose SELP as a potential marker for identifying HSCs at high risk for clonal expansion and myeloid transformation.

Minor question- in fig 4C, 4 replicates are shown while in all other data points from these experiments, there were only 3 replicates- please clarify if these data are generated from different animals

Response: We appreciate the reviewer’s careful attention to the replicate numbers. We clarify that the variation in replicate numbers in Figure 4 (n=3 vs. n=4) is due to the technical requirement of using independent biological cohorts for distinct readouts. Because the high-sensitivity RNA isolation required for qPCR and the multi-parameter surface staining required

for FACS analysis often necessitates different sample processing of rare SLAM-HSC populations, these data points were generated from separate age-matched animals treated under identical protocols. The inclusion of a fourth replicate in Figure 4C was to provide higher resolution for the observed phenotypic trends., and the high consistency across all independent cohorts further validates the robustness of our phenotypic findings.

Dear Dr Suda,

Thank you again for sending us your amended manuscript (EMBOJ-2026-123859R) for reconsideration by The EMBO Journal, together with a point-by-point response to the issues raised during peer-review. Your revised study was sent back to the referees for their scientific reassessment, and we have received detailed re-reports from both of them, which I enclose below. As you will see, the reviewers state that the work has been satisfactorily enhanced by the revisions and they can now support publication at the EMBO Journal, pending minor revision.

Thus, we are pleased to inform you that your manuscript has been accepted in principle for publication in The EMBO Journal.

Please carefully consider the remaining minor point by referee #2 by adjusting the data presentation and discussion of the results in the manuscript text where appropriate.

Also, we now need you to take care of a number of minor issues related to formatting and data annotation, as detailed below.

Please submit a revised version of the manuscript using the link enclosed below.

As you might remember from previous instances, every paper at the EMBO Journal includes a 'Synopsis', displayed on the html and freely accessible to all readers. The synopsis includes a 'model' figure that summarize the article. I would appreciate if you could provide this figure.

Thank you again for giving us the chance to consider your manuscript for The EMBO Journal, I look forward to hearing from you and receiving your final revised version of the manuscript.

Best regards,

Daniel Klimmeck

Daniel Klimmeck PhD
Senior Editor
The EMBO Journal

Formatting changes required for the revised version of the manuscript:

>> Author Contributions: Remove the author contributions information from the manuscript text. Note that CRediT has replaced the traditional author contributions section as of now because it offers a systematic machine-readable author contributions format that allows for more effective research assessment. and use the free text boxes beneath each contributing author's name to add specific details on the author's contribution.

More information is available in our guide to authors.
https://www.embopress.org/page/journal/14602075/authorguide

>> Please update the headings and the order of the manuscript sections to: Abstract, Introduction, Results, Discussion, Methods, Acknowledgements, Disclosure and competing interests statement, References, Figure legends, Expanded View Figure legends

>> Adjust the title of the 'Competing Interests' section to 'Disclosure and Competing Interests Statement'. Please add a

statement: 'T.S. is a member of the EMBO Journal editorial advisory board.'.

>> Remove the article highlights from the manuscript title page and upload them as the synopsis bullet points.

>> References: adjust the reference format to EMBO Journal format, 10 authors et al, and place References after the Disclosure and competing interests statement, before figure legends. DOIs should be removed for all published articles.

>> Dataset EV legends: please upload Table EV1 and EV2 as separate files.

>> Add a Reagents and Tools table to the Methods section, as a separate file using the existing template in the Guide For Authors, listing key reagents, experimental models, software and relevant equipment.

>> Figures in separate files: EV figures should also be uploaded as one file per figure.

>> Biorender: remove info from figure legends and add a dedicated section to the Methods using this format:
Graphics:
(some of the... OR Figure #... OR synopsis) Graphics were created with BioRender.com.

>> Data availability section: please remove referee tokens and make sure that the GEO datasets are made publicly accessible.

>> Consider additional changes and comments from our production team as indicated below:

- DAS:
1. Please note that the specific URLs for datasets (GSE319049, GSE319303) are not provided in the data availability statement.
2. Please note that reviewer access codes for "GSE319049, GSE319303" datasets are not provided in the data availability statement.
>>> now added AD 16.6.26
- Figure legends:
1. Please note that the exact p values are not provided in the legends of 4B.
2. Please indicate the statistical test used for data analysis in the legends of 1D,E; 3C-G; 5B,C,E,F; 6C,D,G; EV-1E; EV-4C; EV-5A.
3. Please note that the box plots need to be defined in terms of minima, maxima, centre, bounds of box and whiskers, and percentile in the legends of figures 1H-J; 6G; EV-5B.
4. Please note that information related to n is missing in the legends 1H-J; 6A,G; EV-1D; EV-5B.

Further information is available in our Guide For Authors: https://link.springer.com/journal/44318/submission-guidelines

----

Referee #1:

The authors have sufficiently addressed all my previous concerns.

Referee #2:

The revised manuscript does address many of the raised concerns adequately- but I'd like further clarification - minimally in the discussion but this point does bother me- the authors state the Selp-low HSCs are more youthful - and one of the main reasons for requesting the PCA plot of the RNA transcriptomes was to see not only to see how the replicates looked, but also to see if the Selp-low HSC were closer transcriptionally to the Young hsc (conversely if the Selp-Hi were even further from young compared to the Selp-low). Clustering analysis showed not only were the selp-low most variable in the replicates, but they were also much further away (on a completely separate branch) from the young and Selp-Hi HSC. Could the authors please address why and how this clustering is driven with the Selp-low seemingly the most different from the young HSC? It seems contradictory to the functional data (again though the transplants from both Selp-hi and Selp-low are not the most robust with n=3 recips and declining chimerism over the timecourse). Perhaps this isn't a major revisions but needs to be addressed to help clarify their interpretations of the results.

Responses to Reviewers

Referee #1:

The authors have sufficiently addressed all my previous concerns.

Response: We appreciate the reviewer's constructive suggestions, which have significantly improved the quality of our manuscript.

Referee #2:

The revised manuscript does address many of the raised concerns adequately- but I'd like further clarification - minimally in the discussion but this point does bother me- the authors state the Selp-low HSCs are more youthful - and one of the main reasons for requesting the PCA plot of the RNA transcriptomes was to see not only to see how the replicates looked, but also to see if the Selp-low HSC were closer transcriptionally to the Young hsc (conversely if the Selp-Hi were even further from young compared to the Selp-low). Clustering analysis showed not only were the selp-low most variable in the replicates, but they were also much further away (on a completely separate branch) from the young and Selp-Hi HSC. Could the authors please address why and how this clustering is driven with the Selp-low seemingly the most different from the young HSC? It seems contradictory to the functional data (again though the transplants from both Selp-hi and Selp-low are not the most robust with n=3 recips and declining chimerism over the timecourse). Perhaps this isn't a major revisions but needs to be addressed to help clarify their interpretations of the results.

Response: We thank the reviewer for the thoughtful comment and completely agree with the reviewer's observation regarding hierarchical clustering, where the $Selp^{low}$ paHSCs (L1 to L3) display higher transcriptomic heterogeneity and separate from the direct sister branch of yHSCs. We postulate that this divergence stems from both important technical attributes of the assay and a highly specific biological reality of this subpopulation:

- Technical Considerations of Low-Input RNA-seq: Given the scarcity of these purified HSCs, we employed optimized low-input amplification. This approach inherently involves extensive PCR pre-amplification, which can increase technical variance and stochastic transcript drop-out, thereby accentuating global transcriptomic distance between samples and youthful controls.
- A Unique Transcriptomic Identity: Biologically, our differential expression analysis reveals that $Selp^{low}$ paHSCs do not merely mimic a young state. Instead, they represent a distinct, transcriptionally active subpopulation with a specialized adaptive program (Figure 3H). This is characterized by two distinct gene expression patterns:

1. Preservation of Core Stemness Programs: Critical regulators of HSC survival, maintenance, and lineage priming (including *Gfi1*, *Runx2*, and *Flt3*) demonstrate a clear trend where Selp^{low} paHSCs maintain or transition toward expression levels seen in yHSCs, whereas these programs are down-regulated or lost in Selp^{high} paHSCs. This directly aligns with our functional transplantation data showing preserved youthfulness.
2. Activation of a Unique Adaptive Signature: Selp^{low} paHSCs reveal a massive, unique upregulation of specific lineage and homing-associated genes (such as *Ccr6*, *Cd79a*, *Cd74*, *Pax5*, and *Tcf7*) exclusively in the Selp^{low} population, which are absent in both yHSCs and Selp^{high} paHSCs.

Finally, we wish to clarify that the global transcriptomic profile and functional performance are distinct readouts. While global clustering is heavily influenced by the entire transcriptome, the functional capacity of HSC might be governed by a refined subset of core functional genes and programs. Our data demonstrate that while the global transcriptomic identity of Selp^{low} paHSCs is divergent due to this specialized adaptive program, the key functional indicators, such as *Gfi1, CD86,* and *Runx2*, remain correlated with a youthful expression profile. Thus, rather than a contradiction, these data demonstrate that Selp^{low} paHSCs utilize a specialized, distinct transcriptional state to actively resist standard aging trajectories and preserve functional capacity within an aged microenvironment.

We have updated the Discussion to explicitly clarify that the global transcriptomic divergence observed in Selp^{low} paHSCs is compatible with their preserved functional youthfulness.

Dear Toshio,

Thank you for submitting the revised version of your manuscript. I have now evaluated your amended manuscript and concluded that the remaining minor concerns have been sufficiently addressed.

I am thus pleased to inform you that your manuscript has been accepted for publication in the EMBO Journal.

Your manuscript will be processed for publication by EMBO Press. It will be copy edited and you will receive page proofs prior to publication. Please note that you will be contacted by Springer Nature Author Services to complete licensing and payment information.

Please note that it is The EMBO Journal policy for the transcript of the editorial process (containing referee reports and your response letter) to be published as an online supplement to each paper. If you do NOT want this, you will need to inform the Editorial Office via email immediately. More information is available here: https://www.embopress.org/transparent-process#Review_Process

You may qualify for financial assistance for your publication charges - either via a Springer Nature fully open access agreement or an EMBO initiative. Check your eligibility: https://www.embopress.org/page/journal/14602075/authorguide#chargesguide

Should you be planning a Press Release on your article, please notify the production team via this form in order to coordinate publication and release dates: https://forms.cloud.microsoft/e/eH4A8JVP6g

If you have any questions, please do not hesitate to contact the Editorial Office. Thank you for your contribution to The EMBO Journal.

Thank you again for this contribution to The EMBO Journal and congratulations on a successful publication! Please consider us again in the future for your most exciting work.

Best regards,

Daniel

Daniel Klimmeck, PhD
Senior Editor
The EMBO Journal
EMBO
Postfach 1022-40
Meyerhofstrasse 1
D-69117 Heidelberg
contact@embojournal.org

*****
Please note that it is The EMBO Journal policy for the transcript of the editorial process (containing referee reports and your response letters) to be published as an online supplement to each paper. If you should prefer removal of any referee-only figures included in the point-by-point response(s), e.g. because they may still be used for future publication or because they have been reproduced from published work by others, please do let us know immediately via response email.
More information is available here: https://link.springer.com/partners/embo-press/editorial-policies#Peer%20review

EMBO Press Author Checklist

Corresponding Author Name: Toshio Suda	
Journal Submitted to: The EMBO Journal	
Manuscript Number: EMBOJ-2026-123859	

USEFUL LINKS FOR COMPLETING THIS FORM
The EMBO Journal - Author Guidelines
EMBO Reports - Author Guidelines
Molecular Systems Biology - Author Guidelines
EMBO Molecular Medicine - Author Guidelines

Reporting Checklist for Life Science Articles (updated January
This checklist is adapted from Materials Design Analysis Reporting (MDAR) Checklist for Authors. MDAR establishes a minimum set of requirements in transparent reporting in the life sciences (see Statement of Task: 10.31222/osf.io/9sm4x). Please follow the journal's guidelines in preparing your
Please note that a copy of this checklist will be published alongside your article.

Abridged guidelines for figures
1. Data
The data shown in figures should satisfy the following conditions:
- the data were obtained and processed according to the field's best practice and are presented to reflect the results of the experiments in an accurate and unbiased manner.
- ideally, figure panels should include only measurements that are directly comparable to each other and obtained with the same assay.
- plots include clearly labeled error bars for independent experiments and sample sizes. Unless justified, error bars should not be shown for technical
- if n<5, the individual data points from each experiment should be plotted. Any statistical test employed should be justified.
- Source Data should be included to report the data underlying figures according to the guidelines set out in the authorship guidelines on Data

2. Captions
Each figure caption should contain the following information, for each panel where they are relevant:
- a specification of the experimental system investigated (eg cell line, species name).
- the assay(s) and method(s) used to carry out the reported observations and measurements.
- an explicit mention of the biological and chemical entity(ies) that are being measured.
- an explicit mention of the biological and chemical entity(ies) that are altered/varied/perturbed in a controlled manner.
- the exact sample size (n) for each experimental group/condition, given as a number, not a range;
- a description of the sample collection allowing the reader to understand whether the samples represent technical or biological replicates (including how many animals, litters, cultures, etc.).
- a statement of how many times the experiment shown was independently replicated in the laboratory.
- definitions of statistical methods and measures:
  - common tests, such as t-test (please specify whether paired vs. unpaired), simple χ2 tests, Wilcoxon and Mann-Whitney tests, can be unambiguously identified by name only, but more complex techniques should be described in the methods section;
  - are tests one-sided or two-sided?
  - are there adjustments for multiple comparisons?
  - exact statistical test results, e.g., P values = x but not P values < x;
  - definition of 'center values' as median or average;
  - definition of error bars as s.d. or s.e.m.

Please complete ALL of the questions below.
Select "Not Applicable" only when the requested information is not relevant for your study.

Materials

Newly Created Materials	Information included in the manuscript?	In which section is the information available? (Reagents and Tools Table, Materials and Methods, Figures, Data Availability Section)
New materials and reagents need to be available; do any restrictions apply?	Not Applicable	

Antibodies	Information included in the manuscript?	In which section is the information available? (Reagents and Tools Table, Materials and Methods, Figures, Data Availability Section)
For **antibodies** provide the following information:<br>- Commercial antibodies: RRID (if possible) or supplier name, catalogue number and or/clone number<br>- Non-commercial: RRID or citation	Yes	Methods

DNA and RNA sequences	Information included in the manuscript?	In which section is the information available? (Reagents and Tools Table, Materials and Methods, Figures, Data Availability Section)
**Short novel DNA or RNA including primers, probes**: provide the sequences.	Yes	Table EV2

Cell materials	Information included in the manuscript?	In which section is the information available? (Reagents and Tools Table, Materials and Methods, Figures, Data Availability Section)
**Cell lines:** Provide species information, strain. Provide accession number in repository **OR** supplier name, catalog number, clone number, and/**OR** RRID.	Not Applicable	
**Primary cultures:** Provide species, strain, sex of origin, genetic modification status.	Not Applicable	
Report if the cell lines were recently **authenticated** (e.g., by STR profiling) and tested for mycoplasma contamination.	Not Applicable	

Experimental animals	Information included in the manuscript?	In which section is the information available? (Reagents and Tools Table, Materials and Methods, Figures, Data Availability Section)
**Laboratory animals or Model organisms:** Provide species, strain, sex, age, genetic modification status. Provide accession number in repository **OR** supplier name, catalog number, clone number, **OR** RRID.	Yes	Methods
**Animal observed in or captured from the field:** Provide species, sex, and age where possible.	Not Applicable	
Please detail **housing and husbandry conditions**.	Yes	Methods

Plants and microbes	Information included in the manuscript?	In which section is the information available? (Reagents and Tools Table, Materials and Methods, Figures, Data Availability Section)
**Plants:** provide species and strain, ecotype and cultivar where relevant, unique accession number if available, and source (including location for collected wild specimens).	Not Applicable	
**Microbes:** provide species and strain, unique accession number if available, and source.	Not Applicable	

Human research participants	Information included in the manuscript?	In which section is the information available? (Reagents and Tools Table, Materials and Methods, Figures, Data Availability Section)
If collected and within the bounds of privacy constraints report on age, sex and gender or ethnicity for all study participants.	Not Applicable	

Core facilities	Information included in the manuscript?	In which section is the information available? (Reagents and Tools Table, Materials and Methods, Figures, Data Availability Section)
If your work benefited from core facilities, was their service mentioned in the acknowledgments section?	Yes	Acknowledgement

Design

Study protocol	Information included in the manuscript?	In which section is the information available? (Reagents and Tools Table, Materials and Methods, Figures, Data Availability Section)
If study protocol has been **pre-registered, provide DOI in the manuscript**. For clinical trials, provide the trial registration number **OR** cite DOI.	Not Applicable	
Report the **clinical trial registration number** (at ClinicalTrials.gov or equivalent), where applicable.	Not Applicable	

Laboratory protocol	Information included in the manuscript?	In which section is the information available? (Reagents and Tools Table, Materials and Methods, Figures, Data Availability Section)
Provide DOI OR other citation details if **external detailed step-by-step protocols** are available.	Yes	Methods, References

Experimental study design and statistics	Information included in the manuscript?	In which section is the information available? (Reagents and Tools Table, Materials and Methods, Figures, Data Availability Section)
Include a statement about **sample size** estimate even if no statistical methods were used.	Yes	Methods, Figures
Were any steps taken to minimize the effects of subjective bias when allocating animals/samples to treatment (e.g. **randomization procedure**)? If yes, have they been described?	Not Applicable	
Include a statement about **blinding** even if no blinding was done.	Yes	Methods
Describe **inclusion/exclusion criteria** if samples or animals were excluded from the analysis. Were the criteria pre-established?<br><br>If sample or data points were omitted from analysis, report if this was due to attrition or intentional exclusion and provide justification.	Not Applicable	
For every figure, are **statistical tests** justified as appropriate? Do the data meet the assumptions of the tests (e.g., normal distribution)? Describe any methods used to assess it. Is there an estimate of variation within each group of data? Is the variance similar between the groups that are being statistically compared?	Yes	Methods, Figures

Sample definition and in-laboratory replication	Information included in the manuscript?	In which section is the information available? (Reagents and Tools Table, Materials and Methods, Figures, Data Availability Section)
In the figure legends: state number of times the experiment was **replicated** in laboratory.	Yes	Figures
In the figure legends: define whether data describe **technical or biological replicates**.	Yes	Figures

Ethics

Ethics	Information included in the manuscript?	In which section is the information available? (Reagents and Tools Table, Materials and Methods, Figures, Data Availability Section)
Studies involving **human participants**: State details of **authority granting ethics approval** (IRB or equivalent committee(s), provide reference number for approval.	Not Applicable	
Studies involving **human participants**: Include a statement confirming that **informed consent** was obtained from all subjects and that the experiments conformed to the principles set out in the WMA Declaration of Helsinki and the Department of Health and Human Services Belmont Report.	Not Applicable	
Studies involving **human participants: ** For publication of **patient photos**, include a statement confirming that consent to publish was obtained.	Not Applicable	
Studies involving experimental **animals**: State details of **authority granting ethics approval** (IRB or equivalent committee(s), provide reference number for approval. Include a statement of compliance with ethical regulations.	Yes	Methods
Studies involving **specimen and field samples:** State if relevant **permits** obtained, provide details of authority approving study; if none were required, explain why.	Not Applicable	

Dual Use Research of Concern (DURC)	Information included in the manuscript?	In which section is the information available? (Reagents and Tools Table, Materials and Methods, Figures, Data Availability Section)
Could your study fall under dual use research restrictions? Please check biosecurity documents and list of **select agents and toxins** (CDC): https://www.selectagents.gov/sat/list.htm	Not Applicable	
If you used a select agent, is the security level of the lab appropriate and reported in the manuscript?	Not Applicable	
If a study is subject to dual use research of concern regulations, is the name of the **authority granting approval and reference number** for the regulatory approval provided in the manuscript?	Not Applicable	

Reporting

The MDAR framework recommends adoption of discipline-specific guidelines, established and endorsed through community initiatives. Journals have their own policy about requiring specific guidelines and recommendations to complement MDAR.

Adherence to community standards	Information included in the manuscript?	In which section is the information available? (Reagents and Tools Table, Materials and Methods, Figures, Data Availability Section)
State if relevant guidelines or checklists (e.g., **ICMJE, MIBBI, ARRIVE, PRISMA**) have been followed or provided.	Yes	Methods
For **tumor marker prognostic studies,** we recommend that you follow the **REMARK** reporting guidelines (see link list at top right). See author guidelines, under 'Reporting Guidelines'. Please confirm you have followed these guidelines.	Not Applicable	
For **phase II and III randomized controlled trials**, please refer to the **CONSORT** flow diagram (see link list at top right) and submit the CONSORT checklist (see link list at top right) with your submission. See author guidelines, under 'Reporting Guidelines'. Please confirm you have submitted this list.	Not Applicable	

Data Availability

Data availability	Information included in the manuscript?	In which section is the information available? (Reagents and Tools Table, Materials and Methods, Figures, Data Availability Section)
Have **primary datasets** been deposited according to the journal's guidelines (see 'Data Deposition' section) and the respective accession numbers provided in the Data Availability Section?	Yes	Data Availability
Were **human clinical and genomic datasets** deposited in a public access-controlled repository in accordance to ethical obligations to the patients and to the applicable consent agreement?	Not Applicable	
Are **computational models** that are central and integral to a study available without restrictions in a machine-readable form? Were the relevant accession numbers or links provided?	Not Applicable	
If publicly available data were reused, provide the respective **data citations in the reference list**.	Yes	References
