# Supplementary material for: P-selectin delineates conserved functional heterogeneity in early hematopoietic stem cell aging in humans and mice
Source: EMBO J. 2026 Aug 5;45(18):6421–49. doi: 10.1038/s44318-026-00887-w (PMC13578734; doi:10.1038/s44318-026-00887-w)
Supplement: Supplementary file 4 [file 44318_2026_887_MOESM7_ESM.pdf]

Expanded View Figures

Figure EV1. Single-cell landscape and age-associated transcriptional features of human hematopoietic stem and progenitor cells (HSPCs).

(**A**) Monocle-based trajectory analysis of human HSPC single-cell RNA-seq data with cells colored by cluster identity. (**B**) The same Monocle trajectory colored by pseudotime, indicating the inferred progression from primitive HSCs toward differentiated progenitors. (**C**) UMAP feature plots displaying expression levels of representative lineage signature genes, highlighting lineage-specific transcriptional patterns. (**D**) Violin plots showing the expression distribution of SELP (top) and HLF (bottom) across defined HSPC and progenitor clusters, illustrating differential expression among cell states. The number of cells (n) analyzed for each cluster is as follows: Baso/Mast-Prog, $n = 391$; E-Prog-1, $n = 1365$; E-Prog-2, $n = 132$; E-Prog-3, $n = 934$; G-Prog-1, $n = 1285$; G-Prog-2, $n = 976$; G-Prog-3, $n = 970$; G/Mono-Prog, $n = 1207$; HSC, $n = 3553$; Ly-Prog-1, $n = 932$; Ly-Prog-2, $n = 677$; Ly-Prog-3, $n = 395$; Ly-Prog-4, $n = 906$; Ly-Prog-5, $n = 881$; Mk-Prog, $n = 239$; Mk/E-MPP, $n = 855$; Mono/cDC-Prog, $n = 184$; MPP-1, $n = 1745$; MPP-2, $n = 1086$; MPP-3, $n = 171$; My-MPP, $n = 728$; pDC-Prog, $n = 903$. (**E**) Dot plot of the top KEGG pathways enriched in old versus young HSCs. Dot size represents the number of genes contributing to each pathway, the x axis indicates the gene ratio, and color intensity reflects the adjusted P value. Pathway enrichment significance was assessed using a hypergeometric test, and P values were adjusted for multiple comparisons using the Benjamini–Hochberg method.

© The Author(s)

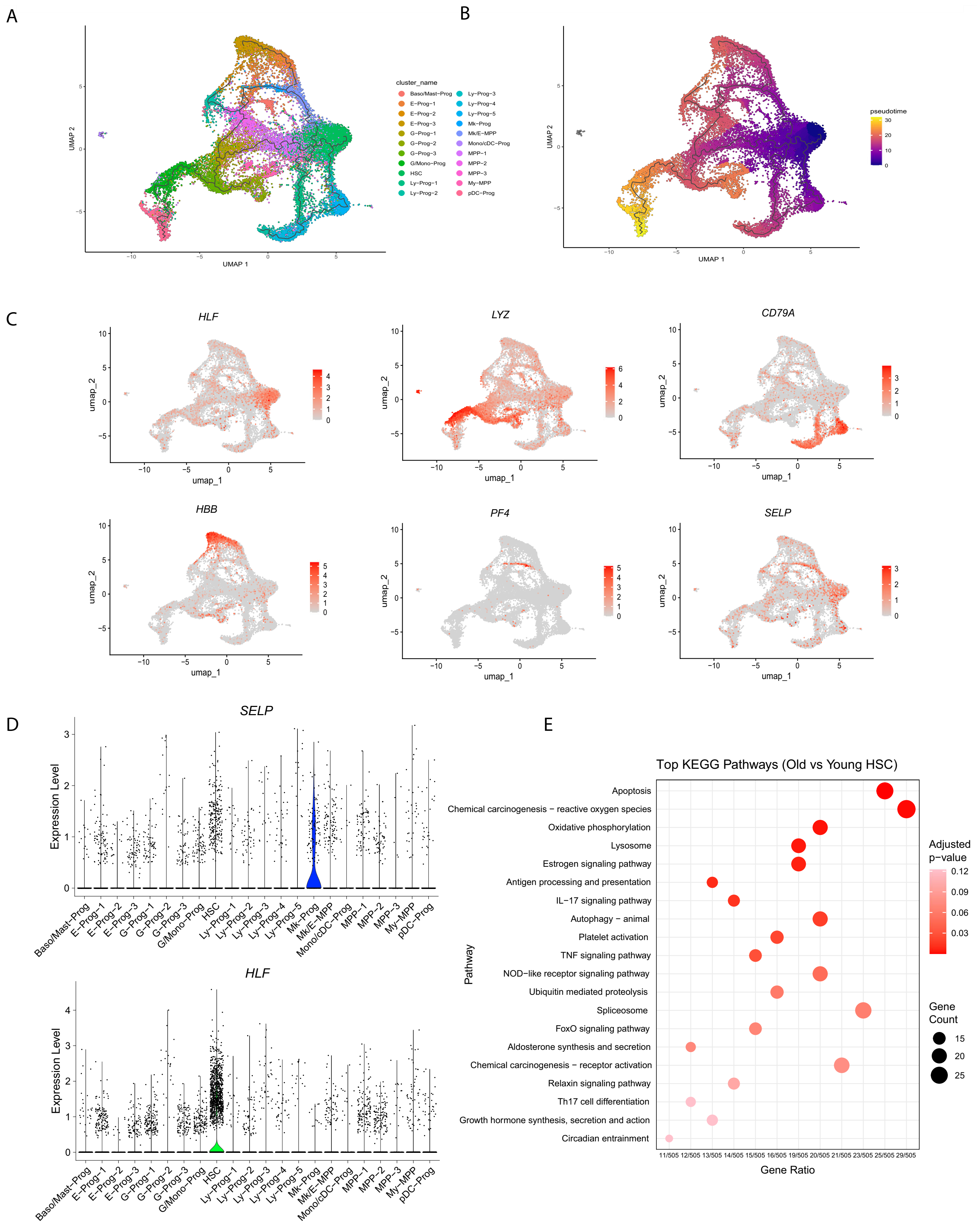
A
cluster_name
Baso/Mast−Prog
E−Prog−1
E−Prog−2
E−Prog−3
G−Prog−1
G−Prog−2
G−Prog−3
G/Mono−Prog
HSC
Ly−Prog−1
Ly−Prog−2
Ly−Prog−3
Ly−Prog−4
Ly−Prog−5
Mk−Prog
Mk/E−MPP
Mono/cDC−Prog
MPP−1
MPP−2
MPP−3
My−MPP
pDC−Prog
UMAP 1
UMAP 2
B
pseudotime
UMAP 1
UMAP 2
C
HLF
LYZ
CD79A
HBB
PF4
SELP
umap_1
umap_2
D
SELP
HLF
Expression Level
E
Top KEGG Pathways (Old vs Young HSC)
Apoptosis
Chemical carcinogenesis − reactive oxygen species
Oxidative phosphorylation
Lysosome
Estrogen signaling pathway
Antigen processing and presentation
IL−17 signaling pathway
Autophagy − animal
Platelet activation
TNF signaling pathway
NOD−like receptor signaling pathway
Ubiquitin mediated proteolysis
Spliceosome
FoxO signaling pathway
Aldosterone synthesis and secretion
Chemical carcinogenesis − receptor activation
Relaxin signaling pathway
Th17 cell differentiation
Growth hormone synthesis, secretion and action
Circadian entrainment
Pathway
Gene Ratio
11/505 12/505 13/505 14/505 15/505 16/505 19/505 20/505 21/505 23/505 25/505 29/505
Adjusted p−value
0.12
0.09
0.06
0.03
Gene Count
15
20
25

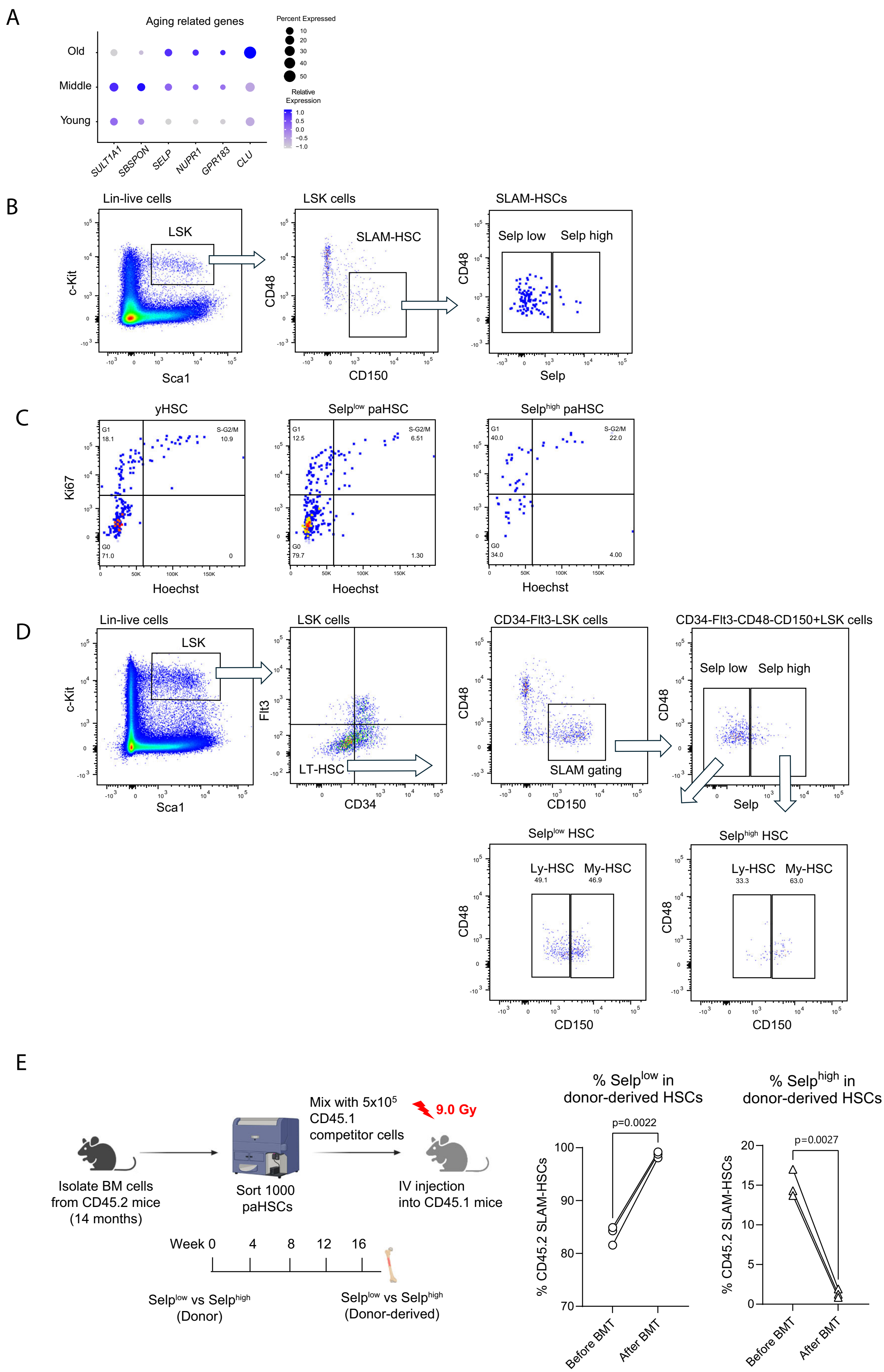
A
Aging related genes
Old
Middle
Young
SULT1A1
SBSPON
SELP
NUPR1
GPR183
CLU
Percent Expressed
Relative Expression
B
Lin-live cells
LSK
c-Kit
Sca1
LSK cells
SLAM-HSC
CD48
CD150
SLAM-HSCs
Selp low
Selp high
Selp
C
yHSC
Selp^low paHSC
Selp^high paHSC
Ki67
Hoechst
D
Lin-live cells
LSK cells
Flt3
LT-HSC
CD34
CD34-Flt3-LSK cells
SLAM gating
CD34-Flt3-CD48-CD150+LSK cells
Selp^low HSC
Selp^high HSC
Ly-HSC
My-HSC
E
Isolate BM cells from CD45.2 mice (14 months)
Sort 1000 paHSCs
Mix with 5x10^5 CD45.1 competitor cells
9.0 Gy
IV injection into CD45.1 mice
Week 0
4
8
12
16
Selp^low vs Selp^high (Donor)
Selp^low vs Selp^high (Donor-derived)
% Selp^low in donor-derived HSCs
% Selp^high in donor-derived HSCs
p=0.0022
p=0.0027
% CD45.2 SLAM-HSCs
Before BMT
After BMT

 © The Author(s)

Figure EV2. Flow cytometric identification and characterization of Selp^{low} and Selp^{high} pre-aged hematopoietic stem cells (paHSCs).

(**A**) Dot plot showing the expression of aging-related genes across young, middle-aged, and old human HSCs. Dot size indicates the percentage of cells expressing each gene, and color intensity represents the average scaled expression level. (**B**) Flow cytometry gating strategy for the identification of Selp-defined HSC subsets. Lineage-negative (Lin$^-$) live cells were gated to identify LSK cells (Lin$^-$Sca1$^+$c-Kit$^+$), followed by SLAM-HSCs (CD48$^-$CD150$^+$). SLAM-HSCs were further subdivided into Selp^{low} and Selp^{high} populations. (**C**) Cell-cycle analysis of young HSCs (yHSC), Selp^{low} paHSCs, and Selp^{high} paHSCs based on Ki-67 and Hoechst staining, showing the distribution of cells across G_0/G_1 and $S/G_2/M$ phases. (**D**) Extended flow cytometry gating strategy to resolve lineage-biased HSC subsets. Lin$^-$ live cells were gated to identify LSK cells, followed by selection of long-term HSCs (LT-HSCs; CD34$^-$Flt3$^-$ LSK). Within the LT-HSC compartment, HSCs were defined as CD48$^-$CD150$^+$ cells and further subdivided into Selp^{low} and Selp^{high} fractions. Each fraction was subsequently resolved into lymphoid-biased HSCs (Ly-HSCs) and myeloid-biased HSCs (My-HSCs) based on differential CD150 expression. (**E**) The left panel depicts the experimental schematic of the competitive bone marrow transplantation (BMT) assay. Right panels describe the comparison of the frequency of Selp^{low} and Selp^{high} paHSCs within the donor-derived SLAM-HSC pool before BMT and 16 weeks after BMT. Data points represent individual recipient mice, and lines connect donor frequencies to their respective post-transplant outcomes. Statistical significance was determined by a two-tailed Student's *t* test, and exact *P* values are shown.

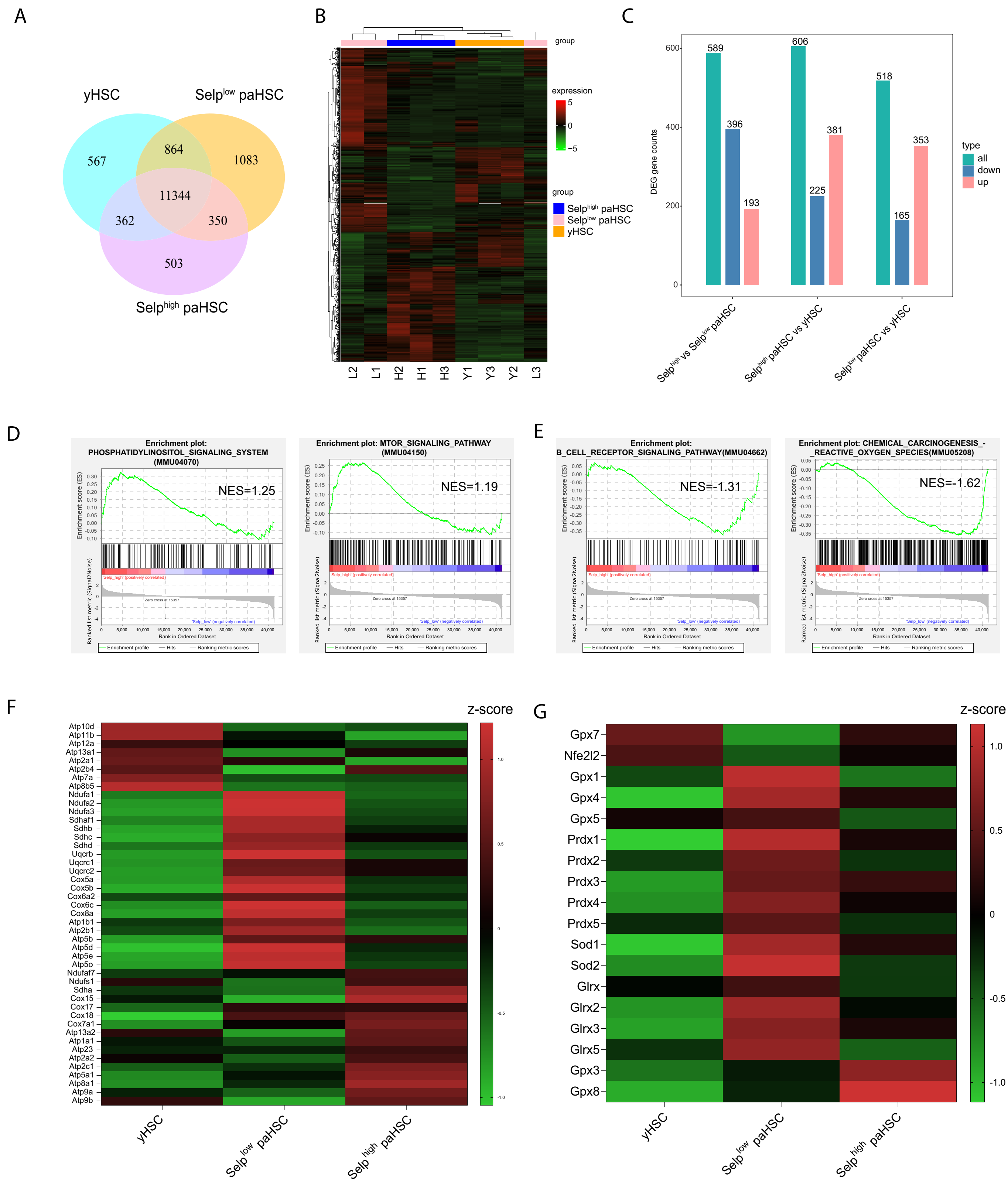
A
yHSC
Selplow paHSC
Selphigh paHSC
567
864
1083
11344
362
350
503
B
group
expression
5
0
−5
group
Selphigh paHSC
Selplow paHSC
yHSC
L2
L1
H2
H1
H3
Y1
Y3
Y2
L3
C
DEG gene counts
589
396
193
606
225
381
518
165
353
type
all
down
up
Selphigh vs Selplow paHSC
Selphigh paHSC vs yHSC
Selplow paHSC vs yHSC
D
Enrichment plot: PHOSPHATIDYLINOSITOL_SIGNALING_SYSTEM (MMU04070)
NES=1.25
Enrichment plot: MTOR_SIGNALING_PATHWAY (MMU04150)
NES=1.19
E
Enrichment plot: B_CELL_RECEPTOR_SIGNALING_PATHWAY(MMU04662)
NES=-1.31
Enrichment plot: CHEMICAL_CARCINOGENESIS_-_REACTIVE_OXYGEN_SPECIES(MMU05208)
NES=-1.62
Enrichment score (ES)
Ranked list metric (Signal2Noise)
Rank in Ordered Dataset
Enrichment profile
Hits
Ranking metric scores
F
z-score
Atp10d
Atp11b
Atp12a
Atp13a1
Atp2a1
Atp2b4
Atp7a
Atp8b5
Ndufa1
Ndufa2
Ndufa3
Sdhaf1
Sdhb
Sdhc
Sdhd
Uqcrb
Uqcrc1
Uqcrc2
Cox5a
Cox5b
Cox6a2
Cox6c
Cox8a
Atp1b1
Atp2b1
Atp5b
Atp5d
Atp5e
Atp5o
Ndufaf7
Ndufs1
Sdha
Cox15
Cox17
Cox18
Cox7a1
Atp13a2
Atp1a1
Atp23
Atp2a2
Atp2c1
Atp5a1
Atp8a1
Atp9a
Atp9b
yHSC
Selp low paHSC
Selp high paHSC
G
z-score
Gpx7
Nfe2l2
Gpx1
Gpx4
Gpx5
Prdx1
Prdx2
Prdx3
Prdx4
Prdx5
Sod1
Sod2
Glrx
Glrx2
Glrx3
Glrx5
Gpx3
Gpx8
1.0
0.5
0
-0.5
-1.0
yHSC
Selp low paHSC
Selp high paHSC

 © The Author(s)

Figure EV3. Transcriptional differences between young HSCs and Selp-defined pre-aged HSC subsets.

(**A**) Venn diagram showing uniquely expressed genes in yHSC, Selp^{low} paHSC, and Selp^{high} paHSC groups. Overlapping regions indicate genes co-expressed in two or more groups. (**B**) Heatmap showing differentially expressed genes (DEGs) across yHSCs, Selp^{low} paHSCs, and Selp^{high} paHSCs. Row-normalized expression levels (Z-score) are shown for biological repeats (Y1-3, yHSCs; L1-3, Selp^{low} paHSCs; H1-3, Selp^{high} paHSCs). (**C**) Bar plot summarizing the number of DEGs between yHSC, Selp^{low} paHSC, and Selp^{high} paHSC groups. Bars indicate the total number of DEGs (all), as well as upregulated (up) and downregulated (down) genes. (**D**, **E**) GSEA plots highlighting KEGG pathways enriched in Selp^{high} (**D**) and Selp^{low} (**E**) paHSCs. (**F**, **G**) Heatmaps showing the scaled expression of mitochondrial metabolism-related genes (**F**) and antioxidant/redox-related genes (**G**) across yHSCs, Selp^{low} paHSC, and Selp^{high} paHSC. Gene expression values are Z-score normalized.

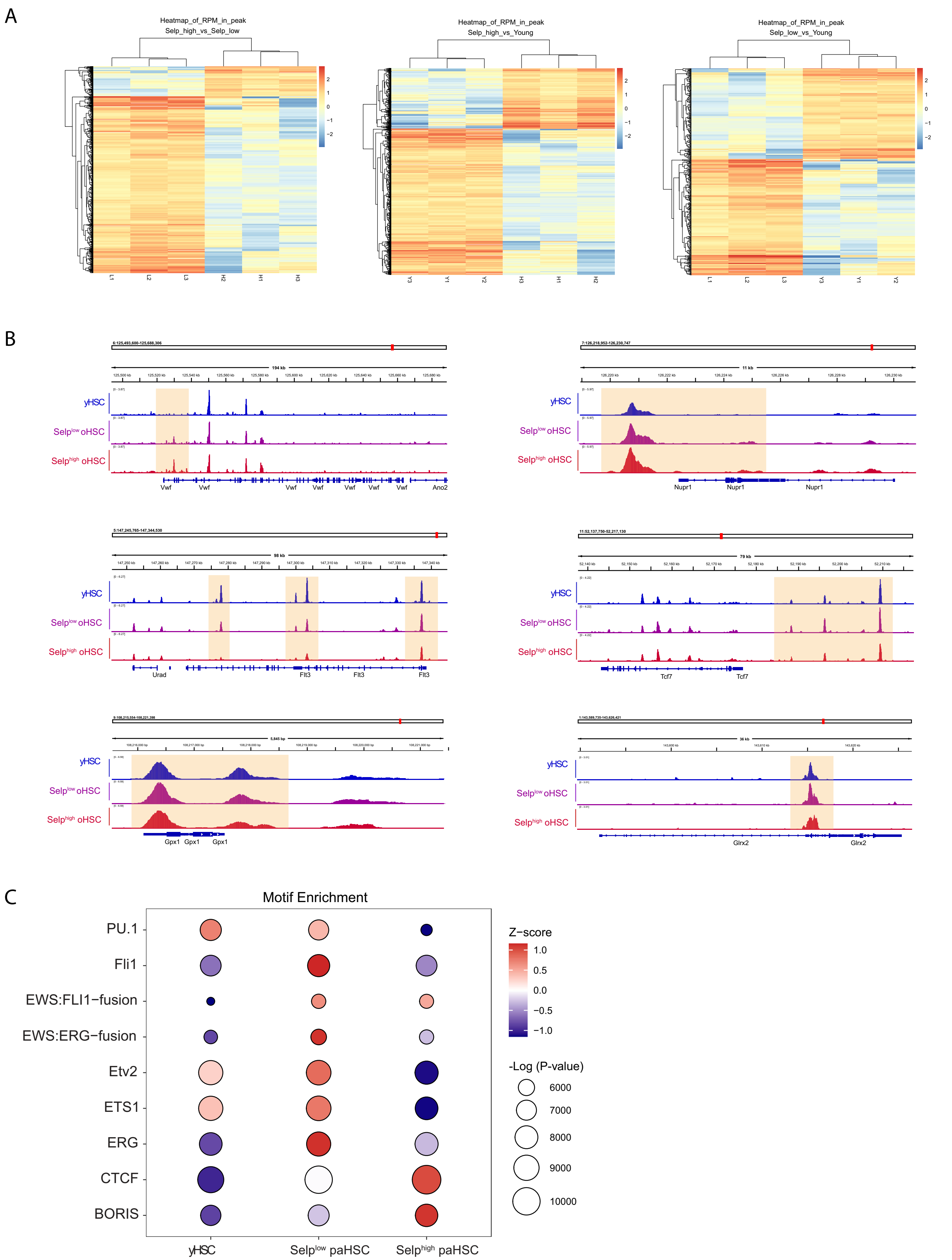
A
Heatmap_of_RPM_in_peak
Selp_high_vs_Selp_low
Heatmap_of_RPM_in_peak
Selp_high_vs_Young
Heatmap_of_RPM_in_peak
Selp_low_vs_Young
B
yHSC
Selp^low oHSC
Selp^high oHSC
Vwf
Ano2
Nupr1
Urad
Flt3
Tcf7
Gpx1
Glrx2
C
Motif Enrichment
PU.1
Fli1
EWS:FLI1−fusion
EWS:ERG−fusion
Etv2
ETS1
ERG
CTCF
BORIS
yHSC
Selp^low paHSC
Selp^high paHSC
Z−score
1.0
0.5
0.0
−0.5
−1.0
-Log (P-value)
6000
7000
8000
9000
10000

 © The Author(s)

Figure EV4. Chromatin accessibility differences and transcription factor motif enrichment in Selp-defined HSC subsets.

(**A**) Heatmaps showing normalized chromatin accessibility across differentially accessible regions comparing Selp^{high} paHSC versus Selp^{low} paHSCs (left), Selp^{high} paHSC HSCs versus yHSCs (middle), and Selp^{low} paHSCs versus yHSCs (right). Rows represent ATAC-seq peaks and columns represent biological repeats; hierarchical clustering was applied to both peaks and samples (Y1-3, yHSCs; L1-3, Selp^{low} paHSCs; H1-3, Selp^{high} paHSCs). (**B**) Representative genome browser tracks showing chromatin accessibility at selected loci in yHSCs, Selp^{low} paHSCs, and Selp^{high} paHSCs. (**C**) Transcription factor motif enrichment analysis based on differentially accessible regions in yHSCs, Selp^{low} paHSCs, and Selp^{high} paHSCs. Dot color indicates motif enrichment Z-score, and dot size reflects −log10(*P* value). Motif enrichment was analyzed using HOMER. *P* values represent hypergeometric tests comparing motif frequencies in target peaks versus GC-matched background sequences.

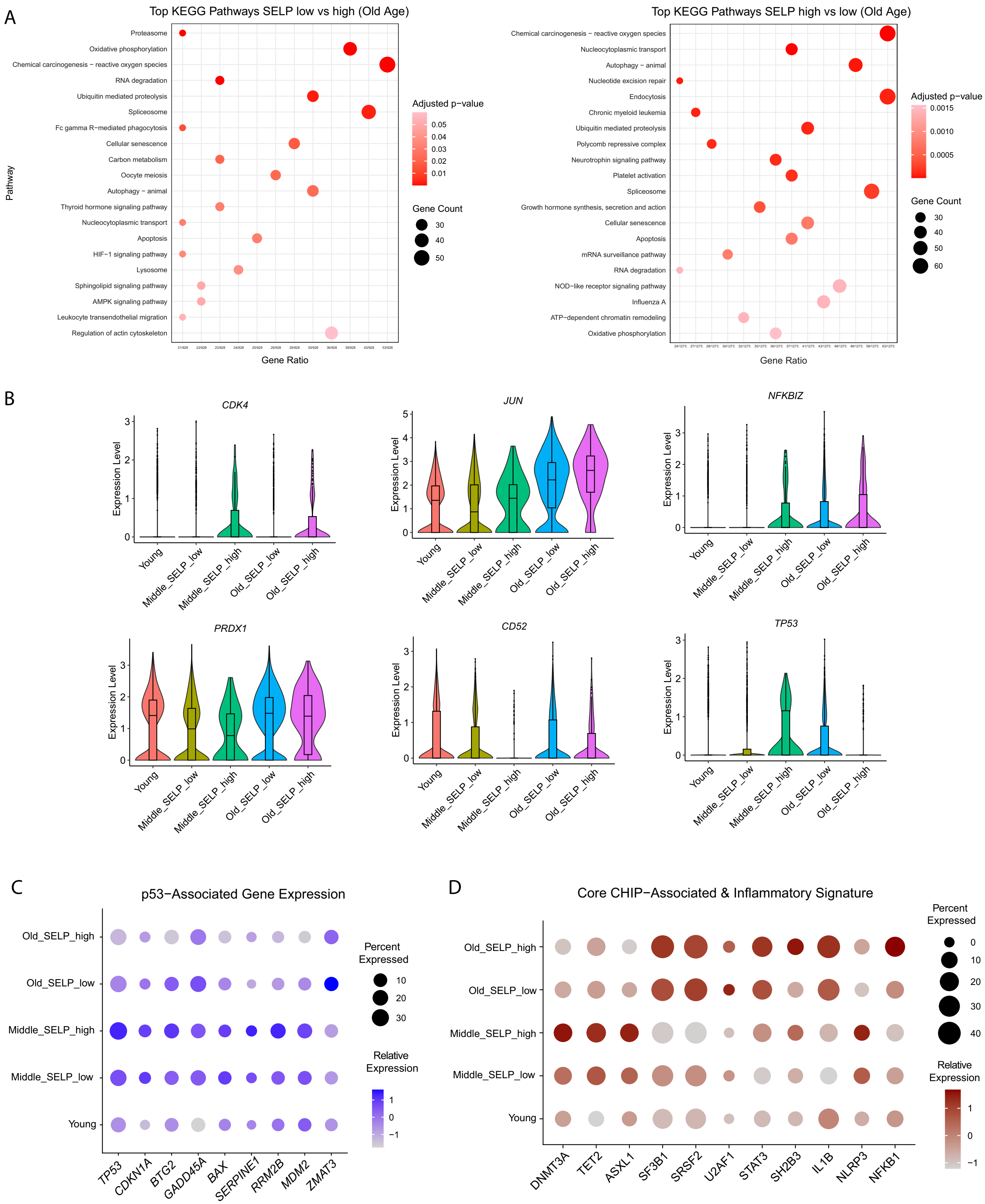
A
Top KEGG Pathways SELP low vs high (Old Age)
Pathway
Proteasome
Oxidative phosphorylation
Chemical carcinogenesis − reactive oxygen species
RNA degradation
Ubiquitin mediated proteolysis
Spliceosome
Fc gamma R−mediated phagocytosis
Cellular senescence
Carbon metabolism
Oocyte meiosis
Autophagy − animal
Thyroid hormone signaling pathway
Nucleocytoplasmic transport
Apoptosis
HIF−1 signaling pathway
Lysosome
Sphingolipid signaling pathway
AMPK signaling pathway
Leukocyte transendothelial migration
Regulation of actin cytoskeleton
Gene Ratio
Adjusted p−value
Gene Count
Top KEGG Pathways SELP high vs low (Old Age)
Chemical carcinogenesis − reactive oxygen species
Nucleocytoplasmic transport
Autophagy − animal
Nucleotide excision repair
Endocytosis
Chronic myeloid leukemia
Ubiquitin mediated proteolysis
Polycomb repressive complex
Neurotrophin signaling pathway
Platelet activation
Spliceosome
Growth hormone synthesis, secretion and action
Cellular senescence
Apoptosis
mRNA surveillance pathway
RNA degradation
NOD−like receptor signaling pathway
Influenza A
ATP−dependent chromatin remodeling
Oxidative phosphorylation
Gene Ratio
Adjusted p−value
Gene Count
B
CDK4
JUN
NFKBIZ
PRDX1
CD52
TP53
Expression Level
Young
Middle_SELP_low
Middle_SELP_high
Old_SELP_low
Old_SELP_high
C
p53−Associated Gene Expression
Old_SELP_high
Old_SELP_low
Middle_SELP_high
Middle_SELP_low
Young
TP53
CDKN1A
BTG2
GADD45A
BAX
SERPINE1
RRM2B
MDM2
ZMAT3
Percent Expressed
Relative Expression
D
Core CHIP−Associated & Inflammatory Signature
Old_SELP_high
Old_SELP_low
Middle_SELP_high
Middle_SELP_low
Young
DNMT3A
TET2
ASXL1
SF3B1
SRSF2
U2AF1
STAT3
SH2B3
IL1B
NLRP3
NFKB1
Percent Expressed
Relative Expression

 © The Author(s)

Figure EV5. SELP stratification reveals distinct stress-response programs in human HSCs upon aging.

(**A**) KEGG pathway enrichment analysis of differentially expressed genes between *SELP*_low and *SELP*_high Old HSCs in human groups. Statistical significance of pathway enrichment was assessed using a hypergeometric test, and *P* values were adjusted for multiple comparisons using the Benjamini–Hochberg method. (**B**) Violin plots showing the expression of representative genes across young, middle-aged, and old HSCs stratified by SELP expression. In all violin plots, the overlaid box plot represents the median (center line), the 25th and 75th percentiles (box bounds), and whiskers extending to the most extreme data points within 1.5 times the interquartile range from the box bound. The number of cells (*n*) analyzed for each group is: Young, $n = 1381$; Middle_SELP_low, $n = 836$; Middle_SELP_high, $n = 73$; Old_SELP_low, $n = 1137$; Old_SELP_high, $n = 126$. (**C**, **D**) Dot plot summarizing the expression of p53-associated genes (**C**) and Clonal Hematopoiesis of Indeterminate Potential (CHIP) and inflammation-associated genes. (**D**) across young, middle-aged, and old human HSCs, further subdivided into *SELP*_low and *SELP*_high fractions. Dot size indicates the percentage of cells expressing each gene, and color intensity represents the average scaled expression level.

© The Author(s)
